# Quantifying environmental co-benefits of nitrogen-based crop restructuring and its implications on India’s interstate trade network

**DOI:** 10.1038/s41467-026-75905-w

**Published:** 2026-08-04

**Authors:** Shekhar Sharan Goyal, Rohini Kumar, Udit Bhatia

**Affiliations:** 1 Department of Earth Sciences, Indian Institute of Technology Gandhinagar, Palaj, Gandhinagar, Gujarat India; 2 Computational Hydrosystems, Helmholtz Center for Environmental Research, UFZ, Leipzig, Germany; 3 Department of Civil Engineering, Indian Institute of Technology Gandhinagar, Palaj, Gandhinagar, Gujarat India; 4 Department of Computer Science & Engineering, Indian Institute of Technology Gandhinagar, Palaj, Gandhinagar, Gujarat India

**Keywords:** Environmental impact, Environmental impact, Agriculture

## Abstract

Addressing sustainability within food systems is challenging due to their multifaceted nature. Here, we evaluate crop-restructuring pathways for Indian agricultural system by integrating nutrient, water, greenhouse gas, economic, and interstate trade-network accounting. We show that the nitrogen-focused crop restructuring strategy reduces nitrogen surplus by 13.4% and water use by 18.6%. This approach maintains baseline calorie production, narrows the benchmarked cereal net-return deficit, and mitigates greenhouse gas emissions by 8.7%. Relative to water-focused restructuring, the nitrogen-focused strategy delivers larger reciprocal co-benefits by about 4.6-fold, resulting in a decrease in socio-environmental costs associated with nitrogen pollution of around $1.19 billion USD. The resulting agricultural restructuring substantially expands alternative-cereal trade in the interstate trade network. Overall, our analysis shows that integrating nutrient management into crop-restructuring strategies can support food-system sustainability in India and other resource-constrained agricultural systems.

## Abstract (web summary)

India’s food system must balance crop production with nitrogen pollution and water scarcity. This study shows that nitrogen focused crop restructuring can reduce pollution, water use and emissions while sustaining calories and reshaping cereal trade.

## Introduction

The challenge of maintaining food security for a rapidly increasing global population is intricately linked to the management of the availability of water and nutrients^1^. With projections that the global population will reach 10 billion by 2050, the increasing demand for food poses major sustainability challenges^2–4^. Current agricultural practices, characterized by overexploitation of freshwater resources^5^, rampant fertilizer nutrient pollution^6^, and increased Agricultural Greenhouse Gas (AGHG) emissions^7,8^, undermine the environmental sustainability of the food system^9^. The emphasis of the green revolution on high-yielding crop cultivars and resource-intensive methods has indeed improved productivity, but at the cost of the environment and ecological balance, highlighting the need for a comprehensive evaluation of environmental consequences and the development of locally adaptable and sustainable agricultural strategies^5,10^. India’s agricultural system offers a critical case for sustainable agriculture, as it supports 17% of the world population^11^ with just 10.2% of its arable land^12^, highlighting the need for sustainable practices. Hence, India provides a unique testbed for examining restructuring strategies because of its dual role as both a major food producer and one of the largest contributors to global nitrogen surpluses and groundwater depletion. Insights from India are therefore globally relevant, both for quantifying environmental co-benefits and for designing transferable frameworks that link nutrient management to trade and food availability.

In the last 50 years, India has managed to triple its food production mainly by intensifying cereal production^4^. However, this achievement has been associated with pronounced environmental repercussions, mainly excessive application of N-fertilizers leading to pollution^3,13,14^, ecological damage^15–17^ and overuse of water resources^18–20^. This situation highlights the pressing need to investigate agricultural practices that improve sustainability on various fronts^21,22^. Recent global analyses highlight the promise of targeted crop switching and redistribution to mitigate irrigation demand^22–28^, conserve fertilizer^29,30^, and enhance biodiversity^31^ while maintaining or increasing production. Thus, while global-scale analyses have quantified trade-offs in crop redistribution^32^, they often remain disconnected from national policy levers and trade dynamics. Our work bridges this gap by focusing on India as a high-impact case study and by developing a framework that can be adapted to other regions to evaluate cross-objective co-benefits and socio-environmental trade-offs. With its growing population, India has been at the forefront of efforts to restructure its Green Revolution era agricultural system; however, studies in the Indian context have primarily focused on minimizing water consumption and alleviating groundwater stress^33–37^, while the equally critical challenge of reducing agricultural nitrogen pollution remains comparatively underexplored.

For informed policymaking, it is essential to analyze synergies and cross-benefits between agronomic efficiency, environmental sustainability, economic viability, and social acceptability, addressing the main challenges of sustainable agriculture^32^. While mineral fertilizers play a key role in ensuring food availability and contribute to achieving Sustainable Development Goal (SDG) 2 (‘Zero Hunger’), they also pose sustainability challenges due to nutrient surpluses^38^. Although synergetic reductions in fertilizer application have been recognized while performing water conservation-based restructuring^28,35^, nitrogen fertilizer application is a poor proxy for environmental footprint calculation^39^. Considering nitrogen surplus, which is the difference between total N input from all sources and N output in crop production, serves as a primary indicator of potential reactive nitrogen (Nr) losses to the environment^13,39–41^. Excess N surplus promotes atmospheric pollution, contravening SDG 3 (‘Good health and well-being’), adversely affecting biodiversity and vegetation (SDG 15, ‘Life on land’), in addition to contributing to climate change through nitrous oxide emissions (SDG 13, ‘Climate action’). Furthermore, they affect water quality, undermining freshwater and marine ecosystems (SDG 6, ‘Clean water and sanitation’ and SDG 14, ‘Life below water’). This oversight highlights a critical gap in identifying strategies that optimally balance N pollution reduction with improved water efficiency.

The importance of considering the surplus of nitrogen to assess agricultural pollution has been recognized beyond simple metrics of fertilizer application^39^. It is argued that focusing on the surplus nitrogen (N) offers more precise insights than considering the application of nitrogen alone, due to its intrinsic link to environmental processes and biological^39,42^. Unlike N inputs, which are variable and subject to human management, the surplus of Nr is directly related to the environmental results, making it a critical factor for investigation^39,43^. Although the recognized role of nutrients in future food supply and their implications for food security have been established, discussions on the management of nutrients at the regional and global levels have been limited^44,45^. Despite progress in agricultural trade-off analysis, its impact on policymaking is hampered by overlooked social and cultural aspects and insufficient multiscale evaluations^32^. For example, optimization models might suggest completely replacing staples such as rice and wheat with alternate cereal crops, which might not be feasible or acceptable. Therefore, it is essential to explore ‘What if’ scenarios that consider the full spectrum of primary and secondary co-benefits from partial substitution of crops. Furthermore, although researchers have extensively quantified virtual water^46,47^ and nutrient footprints^13,48,49^ in within-country and cross-border trade flows, few studies have evaluated how agricultural restructuring (e.g., crop reallocation) would reconfigure trade systems^50,51^. This oversight is critical because changes in agricultural practices are likely to reshape trade networks, affecting the distribution and exchange of agricultural products between regions. Understanding these potential shifts in trade landscapes is essential to designing policies that support sustainable agriculture while maintaining economic viability and food security^35,52,53^.

Against this background, our study investigates the potential for crop redistribution in India, focusing on agricultural restructuring using single- and multi-objective optimization models^35,36^ centered on the minimization of nitrogen surplus and water demand. We quantify cross-benefits in terms of reduced water and nutrient consumption, surplus generation, agricultural greenhouse-gas (AGHG) emissions, and benchmarked cereal net return. We analyze how optimized crop distribution patterns could transform the interstate cereal crop trade network, offering the potential to replace staple crops (rice and wheat) with alternative cereals (sorghum, maize, pearl millet, and finger millet) aligned with environmental and socioeconomic objectives. The analysis is based on an extensive dataset collected from various sources for the period 1997 to 2020 (see “Methods”). It encompasses district-level, crop-specific harvest areas and yields, synthetic fertilizer and manure application rates per hectare, nitrogen atmospheric deposition and biological fixation rates, state-wise cultivation costs, official state-year cereal price benchmarks derived from crop value and production statistics, national crop-level realized-price fills for the few unmatched state-crop combinations, and calorific values of crops (see “Methods”). We focus on six primary cereal crops, rice, maize, wheat, jowar (sorghum), bajra (pearl millet) and ragi (finger millet), which are central to India’s cereal production^34^. By integrating data on nutrient inputs, water requirements, AGHG, and economic parameters, including benchmarked cereal net return and production costs, our methodology aims to delineate sustainable agricultural practices that simultaneously minimize water use and nitrogen surplus (Supplementary Fig. S1). The optimization incorporates agricultural constraints that keep the restructuring environmentally and economically feasible while allowing assessment of interstate cereal transfers. Although demonstrated on India, the methodological design, centered on coupled nitrogen and water optimization, offers a transferable framework that can be adapted to other agrarian economies facing similar pressures.

By considering the pan-India scale, we seek to answer the following key questions: What synergies between nitrogen savings and water conservation can be leveraged while preserving key production and livelihood constraints in India? How does incorporating the cultural significance of maintaining key cereal crops (rice and wheat) influence restructuring outcomes? Furthermore, what are the socio-environmental consequences of the N-centric crop restructuring scheme on the Indian interstate trading system, and the associated N pollution mitigation costs? In addressing these questions, our work contributes in two ways: first, by providing a unified nitrogen and water optimization framework that quantifies synergies and trade-offs across multiple sustainability objectives; and second, by explicitly linking restructuring outcomes to interstate trade networks, thereby showing how shifts in cropping patterns cascade into changes in food flows, redistribution, and the distribution of socio-environmental costs. Using India as a critical case, this study contributes to a deeper understanding of the complexities involved in achieving sustainable agriculture, offering insights that can inform policy, guide future research, and inspire sustainable practices within and beyond India.

## Results

### Analysis of nutritional and nutrient requirements

**Fig. 1:**
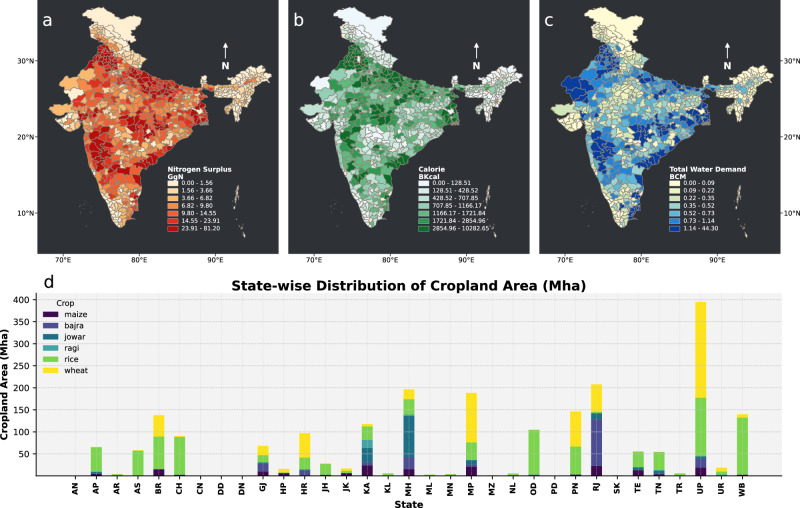
Contemporary dynamics of cereal agriculture in India. **a** District-level nitrogen surplus (Gg N). **b** District-level cereal calorie production (kcal). **c** District-level total water consumption (BCM). **d** State-level spatial distribution of cereal crop varieties, indicating agricultural diversification. District and state boundary geometries used for map visualization were obtained from the *indian-district-boundaries* GitHub repository, released under the MIT License; Copyright (c) 2020 Guneet Narula^91^.

The baseline maps highlight marked spatial variation in nitrogen surplus, calorie production, and crop water demand across districts (Fig. 1a–c). Rice and wheat account for the largest shares of cereal calorie production and crop water demand, with water-demand pressures particularly evident in arid western zones and major rice-producing regions. In examining the crop makeup during the rabi and kharif seasons (November–March and June–October, respectively), rice and wheat emerge as major contributors to India’s calorie production, accounting for 44.2% and 37.6%, respectively, in the selected baseline year 2017 (“Methods”).

Figure 1 d further reveals that these cereals considerably dominate the cropland, covering around 74% of the total area, thus marking their extensive impact on agricultural land usage. From 1960 to 2017, cereal crop production experienced a 344% increase, predominantly driven by rice and wheat^54^. Their proportion of the total cereal production escalated from 64% to 84%, highlighting their increasing dominance in cereal agriculture. This expansion has been supported by enhanced water and nutrient availability, facilitated by groundwater extraction and synthetic fertilizer use, with the north-western states like Punjab and Haryana exhibiting the highest consumptive demands for these resources^34^. Rice stands out as the crop with the highest resource intensity, consuming nearly 1490 m^3^ of water per ton and necessitating 177 kg ha^−1^ of nutrients^13,34^. Diversifying toward cereals such as sorghum (jowar), maize, pearl millet, and finger millet can improve nitrogen and water *use efficiency* in suitable agro-climatic zones. Realized yields and input efficiencies vary with cultivar, management, and local conditions, so the optimization evaluates district-specific per-unit-input metrics while maintaining calorie production and a benchmarked cereal net-return constraint. This spatial variability motivates the district-level restructuring framework.

### Pareto-front analysis and co-benefit

**Fig. 2:**
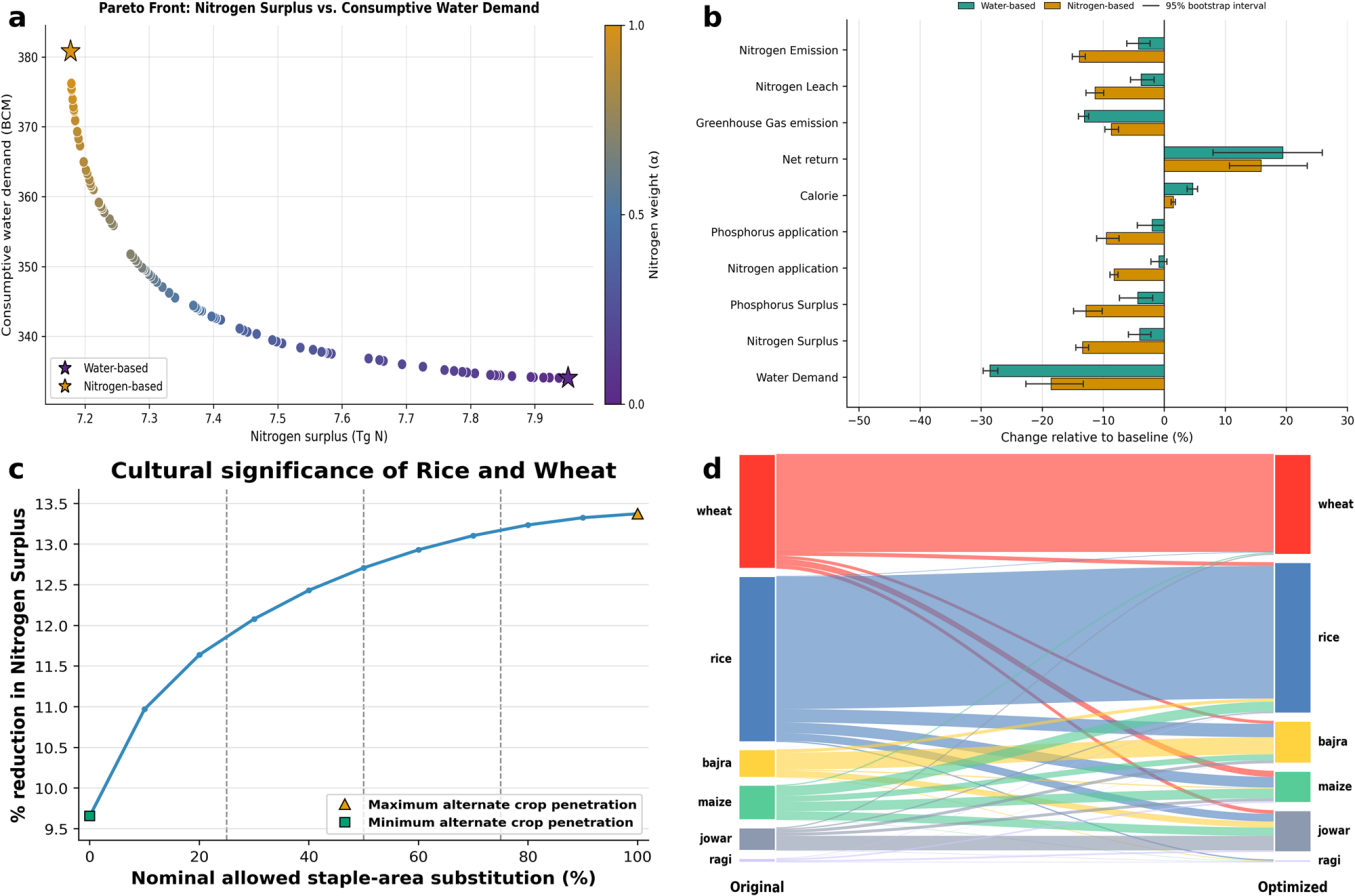
Evaluation of agricultural restructuring strategies in India. **a** Pareto-front analysis detailing the trade-off between nitrogen surplus and total water demand for the combined rabi and kharif system. Each point represents an optimal solution obtained by balancing different levels of water- and nitrogen-minimization objectives. **b** Percentage changes in socio-environmental objectives under water-based and nitrogen-based restructuring relative to the 2017--18 baseline; horizontal whiskers show 95% bootstrap intervals (97.5-2.5 percentiles) derived from *n *= 500 resampled district-level coefficient fields for consumptive water demand and net nutrient application rates. **c** Sensitivity of nitrogen-surplus reduction to relaxed rice and wheat retention constraints. **d** The national level cropland allocation before and after optimization under the nitrogen-surplus minimization strategy.

In addressing sustainability challenges within agriculture, we analyze multiple district-level scenarios to replace rice and wheat, with the aim of balancing the consumptive demands for water and nutrients against the need to preserve benchmarked cereal net return, calorie production, and cultivated area. This initiative aligns with strategic policy objectives geared towards sustainable agricultural practices^34^. Using a multiobjective optimization framework that jointly minimizes water demand and nitrogen surplus, we derive Pareto fronts for rabi and kharif seasons (Supplementary Fig. S2) and an all-season combined frontier (Fig. 2a; see “Methods”). In Supplementary Fig. S2, each point shows the optimal restructured solution for the separate seasons, while Fig. 2a shows the Pareto front for the combined rabi and kharif seasons. Across this combined frontier, nitrogen surplus ranges from 7.177 to 7.952 Tg N, while consumptive water demand ranges from 334.053 to 380.792 BCM. The water-based endpoint defines the lowest-water extreme of this trade-off, whereas the nitrogen-based endpoint defines the lowest-nitrogen extreme. Together, these combined-system results clarify the benefits and trade-offs of restructuring efforts centered on water versus nitrogen-surplus reduction. Additional seasonal endpoint summaries, retained-staple sensitivity, the baseline trade-network template, uncertainty diagnostics, AGHG component attribution, and spatial nitrogen-input diagnostics are provided in Supplementary Figs. S3–S14.

While selecting alternative cereals with the lowest footprints of consumptive water and nitrogen surplus for each district, we also ensure replacement crops sustain the benchmarked cereal net return and maintain baseline calorie adequacy through explicit net-return and calorie-production constraints in our optimization model (see Methods Section 1). At the combined annual scale (Fig. 2b), the water-focused endpoint reduces water demand by 28.6% and nitrogen surplus by 4.0%, equivalent to 133.5 BCM of water savings and 0.33 Tg N less nitrogen surplus relative to baseline. The nitrogen-focused endpoint reduces nitrogen surplus by 13.4% and water demand by 18.6%, corresponding to 1.11 Tg N less nitrogen surplus and 86.8 BCM less water demand. Seasonal endpoint summaries help explain this aggregate pattern (Supplementary Fig. S3). In kharif, the water-focused endpoint reduces water demand by 32.9% while still lowering nitrogen surplus by 7.5%, whereas the nitrogen-focused endpoint reduces nitrogen surplus by 15.5% with a 23.3% water co-benefit. In rabi, the trade-off is narrower: the nitrogen-focused endpoint reduces nitrogen surplus by 9.6% with a 2.6% water co-benefit, while the water-focused endpoint reduces water demand by 14.0% but increases nitrogen surplus by 2.2%. The whiskers in Fig. 2b show 95% bootstrap intervals from propagated district-input uncertainty for all displayed socio-environmental metrics and preserve the qualitative contrast between the two endpoint strategies. Notably, the water co-benefit delivered by nitrogen-focused restructuring (18.6%) remains much larger than the reciprocal nitrogen-surplus co-benefit delivered by water-focused restructuring (4.0%). This seasonal contrast shows that the stronger dual gains in the combined system are driven primarily by kharif rice reallocation, while the combined nitrogen-focused strategy still delivers substantial water savings^1^.

### Impacts on calorie production and net return

Figure 2b illustrates the impact of optimizing water and nutrient use across several sustainability dimensions, including calorie production, fertilizer application, phosphorus surplus, AGHG emissions, nitrogen leaching, nitrogen emissions, and benchmarked cereal net return. At the annual scale, both endpoint strategies preserve and modestly increase aggregate calorie production relative to baseline: by 4.7% under the water-focused endpoint and by 1.5% under the nitrogen-focused endpoint. The larger annual increase under the water-focused endpoint reflects calorie gains in both seasons, while the nitrogen-focused endpoint remains closer to the baseline energy floor by design (Supplementary Fig. S3). Because coarse cereals can expand only within districts where they are already historically cultivated, these gains arise through targeted reallocation rather than unrestricted crop substitution, while rice and wheat still provide the majority of cereal calories.

Under the primary 2017–18 realized-price benchmark, the modeled six-cereal system has a negative aggregate baseline net return. We therefore interpret the net-return metric in Fig. 2b as the change in the benchmarked cereal net-return deficit rather than as conventional profit growth. The water-focused endpoint narrows the aggregate deficit from 161.1 to 129.8 billion rupees, whereas the nitrogen-focused endpoint narrows it to 135.5 billion rupees. The nitrogen-focused improvement is driven mainly by kharif adjustments, while the water-focused improvement is concentrated in rabi (Supplementary Fig. S3).

### Environmental benefits and constraints

Agricultural greenhouse-gas (AGHG) emissions decline under both endpoint strategies, by 13.1% for the water-focused endpoint and by 8.7% for the nitrogen-focused endpoint. Water-focused optimization produces the larger AGHG reduction mainly because stronger rice-area contraction suppresses methane emissions more aggressively (Supplementary Figs. S12–S13). Using the Integrated Model to Assess the Global Environment-Global Nutrient Model (IMAGE-GNM) analysis (Supplementary Fig. S15)^55^, we further translate nitrogen surplus into nitrogen leaching and nitrogen emissions and find that the nitrogen-focused endpoint reduces nitrogen leaching by 11.3% and nitrogen emissions by 13.9% nationwide. Together, these results highlight that water-focused restructuring maximizes AGHG reduction, whereas nitrogen-focused restructuring provides stronger direct control over reactive nitrogen losses while still yielding substantial climate co-benefits.

### Cultural and practical considerations

Rice and wheat hold a deep cultural significance in India, weaving into the fabric of daily life and tradition. Therefore, while mathematical models may propose post-optimal restructuring scenarios leading to the complete replacement of these crops, such outcomes are not practically feasible from an adoption perspective. To account for this, we evaluate a range of retention scenarios that require a specified fraction of state-level rice and wheat area to remain in place, including representative 25, 50, and 75% retention levels across the rabi and kharif seasons. Figure 2c demonstrates that the reduction of nitrogen surplus exhibits a non-linear response to this retained-area constraint, underscoring the cultural importance of these staple crops. Across the combined rabi and kharif system, the attainable reduction in nitrogen surplus ranges from about 9.7% under full state-level retention to about 13.4% when that retained-area floor is fully relaxed. The kharif response remains stronger than the rabi response, reflecting the dominant role of rice in the monsoon season. Overall, Fig. 2d summarises the transition of rice and wheat crop areas to alternative cereals at the national level. Aggregating district-level reallocations nationally, the nitrogen-focused configuration reduces baseline rice area by 8.8% and wheat area by 12.1%, while expanding the combined share of maize, jowar, bajra, and ragi from 24.5% to 32.2% of cereal cropland. Additional sensitivity analyses around the optimization inputs indicate that the qualitative pattern of reallocation remains stable, with widespread replacement of rice and wheat by millets, sorghum, and maize retained across the tested perturbations.

### Monetary gains from nitrogen mitigation

Exploring a restructuring scenario aimed at mitigating the nitrogen surplus during the combined rabi and kharif seasons, we translated the achieved reductions in nitrogen leaching ($${{{{\rm{NO}}}}}_{3}^{-}$$) and nitrous oxide emissions (N_2_O) into equivalent monetary value using data from Sutton et al.^56^ based on 2015 gross national income for India (see “Methods”). Our analysis reveals substantial economic advantages: a yearly savings of $0.08 billion USD in health costs associated with nitrogen leach loss and a $0.98 billion USD annual reduction in losses related to ecosystem degradation. Furthermore, national savings from decreased N_2_O emissions include $24.0 million USD per year in health costs and an additional $0.11 billion USD per year in avoided climate-related damage. When combined, these savings contribute to a potential total of $1.19 billion USD towards mitigating the societal costs of nitrogen pollution. This evidence strongly supports the economic benefits of adopting sustainable agricultural practices to manage nitrogen surplus and highlights the economic rationale behind targeted nitrogen-management strategies^56^, together with the multidimensional value of sustainable agricultural practices for environmental well-being while mitigating financial losses associated with climate and health.

### Trade network restructuring

Trade networks play an important role in supporting national cereal availability and calorie adequacy in India, given the country’s diverse agroclimatic conditions^57^. The disparity in land suitability makes it impractical for individual states to meet cereal demand independently. To recognize this essential disparity, we assess the potential consequences of nutrient-focused restructuring on the trade network. The reconstructed network represents how modeled staple-calorie shortfalls on trade links can be compensated through expanded alternative-cereal flows, while the optimization constraints maintain post-optimization calorie production in each state at least at the baseline level of 2017 (see “Methods”).

**Fig. 3:**
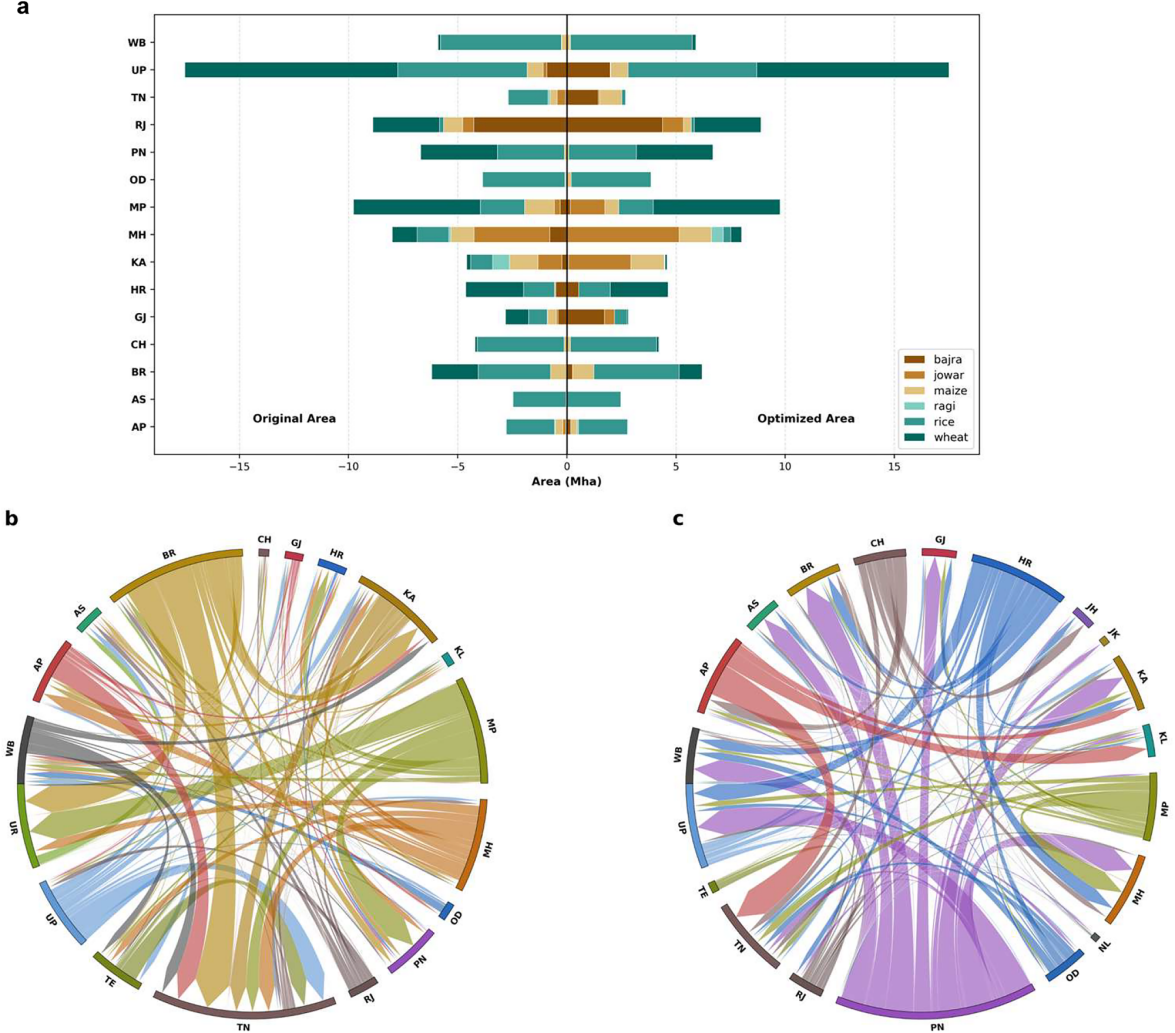
Trade dynamics under nitrogen-surplus minimization. **a** Comparison of original and optimized cropland areas at the state level under the nitrogen-surplus minimization strategy. The left side shows the original distribution of cropland area for each crop by state, while the right side shows the optimized crop area. **b** Restructured interstate trade of combined alternative cereals, including maize, ragi, bajra, and jowar, expressed in kilocalories (kcal). **c** Restructured interstate trade of combined rice and wheat, expressed in kcal, after cropland restructuring for nitrogen-surplus minimization. Arrows indicate flow direction from exporting to importing states; link width is proportional to trade volume; link color denotes exporting state.

Figure 3 a illustrates the state-wise distribution of cropland areas before and after optimization, highlighting adjustments in agricultural land use to reduce nitrogen surplus. These alterations have direct implications for crop production potential and consequently for total caloric production within each state and their trade dynamics. Given that the yield per hectare for each crop remains fixed at baseline-year levels, the area allocated to each crop strongly influences state-level caloric output. Restructuring commonly results in the substitution of wheat and rice with alternative crops, which affects the availability of these staples for interstate trade (Fig. 3c) and expands the role of alternative-cereal exchange (Fig. 3b). The resulting network reorganization remains heterogeneous across states because optimized trade volumes reflect both source-specific production changes and link-level compensation of staple-calorie shortfalls.

By updating the capacities of traditional rice and wheat trade links and compensating positive staple-calorie deficits through alternative-cereal links, including newly introduced connections where needed, we represent modeled trade adjustment under the post-optimization production pattern (see “Methods”). This restructuring increases the modeled volume and relative importance of alternative-cereal exchange (Fig. 3b). Our analysis shows a substantial increase in modeled alternative-cereal trade volume across the network, further highlighting the potential of agricultural restructuring to improve the capacity of India’s interstate trade system while preserving calorie adequacy across states.

## Discussion

The legacy of the Green Revolution in Indian agriculture serves as a poignant backdrop for our study. Initiated to achieve India’s food self-sufficiency, the revolution emphasized the intensive cultivation of wheat and rice. Although it achieved its primary objectives, this transformation has had unintended consequences, including excess nitrogen surpluses and unsustainable water withdrawals, which now stress natural resources and compromise environmental quality. The sustained focus on wheat and rice, driven by historical success and profitability, has not only entrenched these crops’ cultural importance^58^ but also illustrates the complexities involved in moving away from traditional agricultural paradigms. Moreover, the Indian fertilizer subsidy, designed to encourage fertilizer use, has resulted in over-application, leading to soil degradation and water pollution^59^. The impact of these subsidies varies across regions, with many areas facing substantial sustainability challenges due to these enduring practices. The regional variability in these impacts underscores the urgent need for restructuring agricultural practices.

As we advocate for such restructuring with nutrient surplus reduction strategies, the importance of pragmatic and context-aware approaches cannot be overstated. Legacy crops have substantial economic, nutritional and cultural value; therefore, restructuring must support livelihoods, nutritional needs, and cultural preferences. While previous studies have focused on the redistribution of the Indian agricultural system with an emphasis on water-saving approaches^36^, our findings suggest that nitrogen surplus reduction not only reduces nutrient pollution but also unlocks substantial water savings and co-benefits. Recognizing the synergy between water and nutrient management, our optimization schemes aim to identify sustainable practices that respect legacy crops while maximizing the benefits of nitrogen surplus reduction.

Using extensive datasets from government-led farmers’ surveys in India, we demonstrated that an approach focused on reducing nitrogen surplus could also lead to substantial water savings. This provides a clear path for sustainable agricultural restructuring. These models balance several cross-benefit goals of nutrient management and water conservation, suggesting that prioritizing nitrogen-surplus reduction can effectively address broader environmental sustainability goals (for example, water savings and AGHG reduction) while preserving benchmarked cereal net return under the selected price-and-cost environment. This approach not only mitigates the environmental impacts associated with nutrient losses and water scarcity but also presents a holistic strategy to improve agricultural sustainability, including enhanced trade volumes of alternative crops such as maize, ragi, bajra, and jowar. Coupling our optimized agricultural production with the interstate trade network reveals system-wide propagation of crop restructuring that extends beyond regional outcomes, yielding national-scale socio-economic and socio-cultural co-benefits through direct and indirect reductions in the nation’s water and nutrient footprints.

Several limitations qualify the analysis. The calorie constraint preserves baseline state-level caloric output and serves as an energy-availability floor within the optimization. Nutritional quality is not optimized directly; instead, it is discussed using the differing protein and micronutrient profiles of the selected cereals, particularly the higher iron and calcium content of coarse cereals relative to polished rice^60,61^.

Crop-group scope is another limitation. The present analysis focuses on cereal-to-cereal restructuring within the major traded cereal system, where district-season allocation and interstate trade can be parameterized consistently. Pulses and other legumes remain important for nitrogen management and dietary diversification^61–63^. Extending the framework to mixed cereal-pulse systems will require harmonized district-season yield, input, revenue, nutrient-composition, and trade data for additional crop groups.

More broadly, while our nutrient-centric approach is grounded in the best available national-scale data, greater spatial and temporal disaggregation of fertilizer, manure, and socio-economic information would allow more targeted interventions. The transition towards nutrient-focused restructuring must also consider local food demand, the structure of farm holdings and cooperatives, procurement policies, rationing systems, and public price support, all of which influence adoption feasibility. Because the optimization covers only the six modeled cereals, the economic term should be interpreted as benchmarked net return for this cereal portfolio rather than total farm-household income or realized crop-by-crop farm-gate margins. We use the 2017–18 realized-price benchmark as the primary revenue input because it incorporates state-level crop-price heterogeneity observed in official value-of-output and production statistics, and is therefore closer to the realized market setting than a uniform national MSP. This choice reduces the risk that crop-specific differences in actual price realization are obscured by a common national support-price schedule. At the same time, these prices are state-year averages, not district farm-gate transaction prices, and cultivation costs are state-crop C2 benchmarks applied uniformly within states. The resulting margins should therefore be read as a consistent accounting layer for maintaining the economic floor in the optimization, rather than as local profit estimates. Supplementary Fig. S16 documents the realized-price departures from MSP and the corresponding terms-of-trade structure around the primary revenue benchmark. MSP remains policy-relevant as an administratively defined support-price benchmark, especially for evaluating procurement design and incentives for coarse cereals; for this reason, we retain the district-MSP comparison in Supplementary Figs. S17, S18. The MSP-based results should therefore be read as a policy-support benchmark conditional on support-price realization, whereas the realized-price results provide the primary benchmark for market-facing interpretation. The preservation of endpoint ordering across the realized-price and MSP benchmark analyses strengthens the main biophysical conclusion that nitrogen-centered restructuring yields meaningful water co-benefits while directly reducing nitrogen surplus. Supplementary Fig. S19 and Supplementary Methods 1 report the fixed-allocation bootstrap envelope around the main Pareto frontier, and Supplementary Fig. S20 together with Supplementary Discussion 1 reports the seasonally disaggregated reallocation summary underlying the annual panel. The treatment of the state-level C2 production-cost benchmark and the annual cropland-level BNF input term is clarified in Supplementary Methods 2, 3, respectively. Several parameters used to identify optimal strategies remain uncertain due to climatic variability and regional differences in agricultural practices and policies. The endpoint bootstrap intervals in Fig. 2b and the sensitivity analyses described in the Methods quantify part of this uncertainty, although more detailed datasets would strengthen parameter robustness. In addition, our analysis does not differentiate crop genetic varieties, which can vary in water- and nutrient-use efficiency and thereby affect projections of productivity and environmental impacts. Moreover, the nitrogen leaching and emission coefficients derived from the global IMAGE-GNM model are constrained by coarse spatial resolution and generic parameterization, which may not fully capture the heterogeneity of Indian agroecosystems. Finally, practical implementation requires on-the-ground validation, incorporating farmer perspectives, behavioral responses, and socio-economic incentives into the modeling framework.

Future research could extend our framework by integrating state-specific procurement incentives, emerging subsidies, and dynamic pricing, particularly for millets, in domestic and international markets; assembling longer historical datasets beyond the 2017 baseline to enable comparative assessments of structural change; and adopting probabilistic optimization to represent interannual variability in yields, cultivated area, and revenues under climatic and market uncertainty. As data become available, explicit varietal response functions and trait information could be incorporated to refine estimates of productivity and environmental outcomes. Collectively, these advances would further test and generalize the unified nitrogen and water framework proposed here.

In conclusion, centering agricultural restructuring on nitrogen surplus provides a single, tractable lever that unifies nutrient and water objectives within one optimization framework, yielding concurrent gains in water conservation and pollution mitigation while preserving benchmarked cereal net return and respecting cultural constraints on staple crops. This integrated lens translates directly into policy instruments, including procurement and MSP design, targeted incentives for coarse cereals, and state-level planning of trade flows, offering a coherent path from modeling evidence to implementation at the national scale. Although developed for India, the framework is generalizable to other regions facing coupled nutrient and water pressures. Embedding the approach in participatory, on-farm validation and iterative policy design can accelerate an equitable transition away from resource-intensive production systems. Taken together, our results provide a foundation for aligning calorie adequacy, broader nutritional security, and environmental sustainability while advancing multiple SDGs in tandem.

## Methods

Our study uses a linear multi-objective optimization model to assess cereal-crop restructuring in India. The model reallocates existing district-season cereal area among six focal cereals while minimizing nitrogen surplus and consumptive water demand, preserving state-level calorie production as an energy-availability safeguard, and maintaining benchmarked cereal net return. The workflow integrates district-level crop area and yield data for 1997–2020, crop-specific synthetic fertilizer and manure application rates^13,64^, atmospheric nitrogen deposition, cropland-level biological nitrogen fixation input terms, state-level crop-specific C2 production-cost benchmarks^64^, official state-year cereal price benchmarks derived from value-of-output and production statistics, national crop-level realized-price fills for the few unmatched state-crop combinations, and crop calorie values (Supplementary Fig. S1 and Supplementary Tables 1–12). The six modeled cereals are rice (Oryza sativa), maize (Zea mays), wheat (Triticum aestivum), jowar (Sorghum vulgare), bajra (pearl millet), and ragi (finger millet), which together represent most cereal production in India. The different components of our methodology are detailed below.

### Resource calculation for baseline scenario

To establish a baseline for our study, we selected 2017 as the reference year^65^, against which all changes in water, nutrients, benchmarked cereal net return, calories and agricultural greenhouse gas emissions (AGHG) were measured. The choice of the baseline year is mainly determined by the availability and quality of the underlying data. We obtained crop yield and harvested area data for the rabi (non-monsoon) and kharif (monsoon) seasons for the six focal cereals from the Directorate of Economics and Statistics crop-reporting portal^66^. The dataset includes 664 districts, covering approximately 87% of India’s land area^66^. The source data report five planting seasons: winter, autumn, kharif, rabi, and summer. For this analysis, winter was grouped with rabi, and summer and autumn were grouped with kharif^66^. The resulting two-season classification follows the major Indian cropping seasons: kharif (June–October; monsoon) and rabi (November–March; winter). We calculated nitrogen (N) and phosphorus (P) input rates from synthetic fertilizers, manure, the cropland-level BNF input term, and atmospheric deposition. Crop-specific plot-level N and P fertilization rates, together with manure application rates, were obtained from the Government of India’s Cost of Cultivation survey. We adapted the aggregation approach of Davis et al.^35^ to derive district-level crop-specific fertilizer and manure input rates in kg ha^−1^. Where district observations were unavailable, state-level crop averages were used; where state-level observations were unavailable, national crop averages were used. The district-level input for district *i* in state *j*, crop *c*, and year *t* was calculated as:

1 $${Z}_{i,j,c,t}=\frac{{\sum }_{v}\left({K}_{v,i,j,c,t}\cdot {Z}_{v,i,j,c,t}\right)}{{\sum }_{v}{K}_{v,i,j,c,t}},$$

where *Z*_*v*,*i*,*j*,*c*,*t*_ is the input amount reported for plot *v*, and *K*_*v*,*i*,*j*,*c*,*t*_ is the corresponding cluster weight in the Cost of Cultivation dataset^67,68^. Manure application rates from the plot data were converted to nitrogen and phosphorus equivalents using uniform nutrient-content factors of 0.5% N and 0.3% P^69^.

Biological Nitrogen Fixation (BNF) refers to the microbial conversion of atmospheric nitrogen into reactive nitrogen inputs to cropland^70^. The BNF term used here is the annual cropland nutrient-budget input reported in the FAOSTAT Cropland Nutrient Budget dataset^4^, harmonized in kg N ha^−1^ yr^−1^ and merged by year into the district-crop database. Because the focal crops are cereals, this term represents an exogenous cropland nutrient-budget input, not crop-specific symbiotic fixation by cereal plants. The 2017 baseline optimization applies the same annual FAOSTAT BNF value across the six focal cereals; the adopted value is reported in Supplementary Table 12 and clarified further in Supplementary Methods 3.

The quantification of atmospheric nitrogen (N) deposition, encompassing both dry and wet deposition of NHx and NOy, was derived from atmospheric chemical transport models. We utilized data from the National Center for Atmospheric Research (NCAR), specifically from the Chemistry-Climate Model Initiative (CCMI), which forms part of the input datasets for Model Intercomparison Projects (input4MIPS)^71^. This dataset provides monthly data from 1850 to 2014, with a spatial resolution of 1.9^∘^ latitude by 2.5^∘^ longitude. The data were interpolated to the required district resolution using nearest-neighbor interpolation and aggregated to an annual time step.

Crop nutrient uptake was defined as the nutrient embedded in harvested grain^45^. We used crop nutrient-content values from Supplementary Table 2^72^ to estimate harvested nutrient output:

2 $$N{u}_{output(i,j,c,t)}=P{r}_{i,j,c,t}*N{u}_{content(i,j,c,t)}$$

where *Nu*_*output*(*i*, *j*, *c*, *t*)_ represents the nitrogen and phosphorus embedded in the produced crop. *Pr*_*i*,*j*,*c*,*t*_ is the quantity of crop produced in kg, and *Nu*_*content*(*i*, *j*, *c*, *t*)_(kg/kg) represents the nutrient content. *Nu*_*output*_ is only considered for the part of the produced crop (grain).

District-level N surplus (NS; kg N ha^−1^ yr^−1^) and P surplus (PS; kg P ha^−1^ yr^−1^) were calculated as applied nutrient inputs minus harvested grain nutrient output^72^:

3a $$\begin{array}{l}{{{{\rm{NS}}}}}_{(i,j,c,t)}={N}_{{{{{\rm{DEP}}}}}_{(i,j,t)}}+{N}_{{{{{\rm{FERT}}}}}_{(i,j,c,t)}}+{N}_{{{{{\rm{BNF}}}}}_{t}}\\+{N}_{{{{{\rm{MAN}}}}}_{(i,j,c,t)}}-{N}_{{{{{\rm{output}}}}}_{(i,j,c,t)}}\end{array}$$

3b $${{{{\rm{PS}}}}}_{(i,j,c,t)}={P}_{{{{{\rm{FERT}}}}}_{(i,j,c,t)}}+{P}_{{{{{\rm{MAN}}}}}_{(i,j,c,t)}}-{P}_{{{{{\rm{output}}}}}_{(i,j,c,t)}}$$

where *NS*_(*i*, *j*, *c*, *t*)_ is the N surplus for crop *c* in district *i* and state *j* in year *t*; *N*_DEP_ is atmospheric nitrogen deposition; *N*_FERT_ and *P*_FERT_ are synthetic fertilizer inputs; *N*_BNF_ is the annual cropland-level BNF input term from FAOSTAT; *N*_MAN_ and *P*_MAN_ are manure-derived N and P inputs; and *N*_output_ and *P*_output_ are harvested grain nutrient outputs. All terms are expressed per hectare per year in the corresponding nutrient units.

Long-term averages were utilized to fill in missing values for district fertilizer and manure application rates. Leveraging the principle of nutrient mass balance, we estimated the nutrient surplus (*Nu*_*sur*_) within the soil, representing the disparity between *Nu*_*input*_ and *Nu*_*output*_. This surplus encapsulates nutrients persisting in the soil post-crop production. While a fraction of *Nu*_*sur*_ is recycled within the soil, a substantial portion is prone to environmental loss, potentially manifesting as nitrogen and phosphorus losses in various forms, such as nitrous oxide emissions into the atmosphere and nitrate and phosphorus leaching into surface and groundwater sources^13,38,73^. Recognized as an environmental pollution indicator, nutrient surplus also functions as a proxy for virtual nutrient pollution, a measure encompassing the nutrient quantity lost to the environment throughout the entire production cycle of a specific crop commodity^39,74^. The method for surplus calculation is known to introduce substantial uncertainties globally, owing to variations in estimates of crop nitrogen content, biological nitrogen fixation (BNF), and manure management practices. These factors are documented at various resolutions and detailed in the literature^13,75^.

We calculated crop-specific total crop water demand (CWD) for 2017 by combining blue and green water components from Kampman et al.^76^. Following Dalin et al.^77^, state-level CWD coefficients were adjusted to district-year yield conditions:

4 $$CW{D}_{i,j,c,s,t}=CW{D}_{j,c,s,2000}\cdot \frac{{Y}_{j,c,s,2000}}{{Y}_{i,j,c,s,t}}$$

where *i*, *j*, *c*, *s*, and *t* denote district, state, crop, season, and year, respectively; *CWD*_*i*,*j*,*c*,*s*,*t*_ is district-level crop water demand; *Y* is crop yield; and *CWD*_*j*,*c*,*s*,2000_ is the Kampman et al.^76^ state-season crop water-demand coefficient for 2000.

We estimated the benchmarked district cereal net return as sales revenue minus production cost. Production costs use crop-specific state-level C2 cost-of-production benchmarks from the Government of India *Agricultural Statistics at a Glance 2017*^64^. These values are reported in Rupee quintal^−1^ and were merged to district observations by state and crop. Specifically, the benchmark uses the source-table cost-of-production entry reported on the C2 basis in Rupee quintal^−1^, rather than the separate per-hectare cultivation-cost entries. Because no district-level production-cost series is available, the same state-crop C2 benchmark was applied to all districts within a state. The DES C2 concept includes both paid-out costs and imputed owned-resource costs; the relevant cost concepts and source-table fields are summarized in Supplementary Tables 10, 11^68^, and the operational interpretation is detailed in Supplementary Methods 2. Total district production cost was calculated as:

5 $$C{p}_{i,j}^{{{{\rm{total}}}}}={\sum}_{c}\left(10\times {\Pr }_{i,j,c}\times C{p}_{j,c}^{{{{\rm{prod}}}}}\right)$$

where $C{p}_{i,j}^{{{{\rm{total}}}}}$ is total production cost (Rupee), Pr_*i*,*j*,*c*_ is crop production in tonnes, $C{p}_{j,c}^{{{{\rm{prod}}}}}$ is the state-crop C2 production-cost benchmark in Rupee quintal^−1^, and the factor of 10 converts tonnes to quintals.

Sales revenue used the 2017–18 benchmark selling price $S{P}_{j,c}^{{{{\rm{bench}}}}}$ (Rupee quintal^−1^). For each state-crop combination with a usable official realized price, $S{P}_{j,c}^{{{{\rm{bench}}}}}$ was calculated as the state-year crop value of output divided by state-year crop production. Where a direct state-crop price was unavailable or unusable, we applied the corresponding all-India crop-level realized price for 2017–18 so that the benchmark retained complete cereal coverage without introducing district-level price imputation. Using realized unit prices reduces the risk that crop-specific differences in actual price realization are obscured by a common national support-price schedule. MSP values are therefore retained as a separate policy-support benchmark, whereas the realized-price series defines the primary revenue input. Supplementary Table 3 reports the MSP reference values, Supplementary Fig. S16 documents realized-price departures from MSP and indicative terms of trade, and Supplementary Figs. S17, S18 provide the district-MSP comparison benchmark. Sales revenue was then calculated as:

6 $$S{R}_{i,j}={\sum}_{c}\left(10\times S{P}_{j,c}^{{{{\rm{bench}}}}}\times P{r}_{i,j,c}\right)$$

where *SR*_*i*,*j*_ is district sales revenue (Rupee). The benchmark price is applied uniformly to districts within each state-crop pair because realized prices are available at state-year, not district, resolution.

Benchmarked district cereal net return was calculated as sales revenue minus total production cost^28,36^:

7 $${B}_{i,j}=S{R}_{i,j}-C{p}_{i,j}^{{{{\rm{total}}}}}$$

where *B*_*i*,*j*_ is benchmarked cereal net return for district *i* in state *j*. For the optimization constraint, the corresponding state-crop net-return coefficient is:

$${\pi }_{j,c}=10\left(S{P}_{j,c}^{{{{\rm{bench}}}}}-C{p}_{j,c}^{{{{\rm{prod}}}}}\right),$$

where *π*_*j*,*c*_ is expressed in Rupee tonne^−1^.

District-level total calorie production (kcal) was computed as:

8 $${K}_{{{{\rm{cal}}}},i}={\sum}_{c=1}^{n}{\Pr }_{i,c}\times {{{{\rm{kc}}}}}_{c}$$

Here, Pr_*i*,*c*_ is crop production in tonnes, and kc_*c*_ is the calorie density (kcal tonne^−1^) for crop *c* (Supplementary Table 4). Within the optimization framework, this calorie term defines a state-level energy-availability floor; broader dietary quality and nutritional security require additional nutrient-specific targets.

### Setting up the optimization algorithm

We formulated the crop-restructuring problem as a set of linear optimization models. The decision variable *x*_*i*,*j*,*c*,*s*_ denotes the cultivated area allocated to crop *c* in district *i*, state *j*, and season *s*. Let *D*_*j*_ be the set of districts in state *j*, *C* = {rice, wheat, maize, bajra, jowar, ragi}, and *S* = {rabi, kharif}. The model reallocates the existing district-season cereal area among the six focal cereals, while holding total district-season cereal area fixed and allowing only cereals with historical presence in the district-season crop set. Crop yields, nitrogen-surplus coefficients, water-demand coefficients, calorie densities, and net-return coefficients are treated as fixed 2017 baseline coefficients. The constraints preserve state-season calorie production as an energy-availability floor and maintain benchmarked cereal net return under the 2017–18 revenue benchmark described above. Broader dietary quality and nutritional security are not optimization objectives. For combined annual summaries, optimized Rabi and Kharif outputs are summed after solving the seasonal allocation problem.

We solved two single-objective models, one minimizing nitrogen surplus and one minimizing consumptive water demand, and a multi-objective weighted-sum model that balances the two objectives. For the multi-objective frontier, nitrogen surplus and water demand were normalized by their 2017 baseline totals inside the weighted objective so the two quantities could be combined despite their different units. We report the resulting Pareto frontier in absolute units (Tg N and BCM) and use percentage changes relative to the 2017 baseline for endpoint co-benefit summaries.

A single-objective optimization model was developed to minimize total nitrogen surplus (*F*_*N*_), with cultivated area as the decision variable^33^. This was achieved while maintaining or enhancing state-level calorie production and benchmarked cereal net return. The nitrogen-surplus objective is:

Objective function:

9 $$\min \quad {F}_{N}(x)={\sum}_{s\in S}{\sum}_{j}{\sum}_{i\in {D}_{j}}{\sum}_{c\in C}{x}_{i,j,c,s}\,N{S}_{i,j,c,s}$$

Here, *NS*_*i*,*j*,*c*,*s*_ is the per-hectare nitrogen-surplus coefficient (kg N ha^−1^) for crop *c* in district *i*, state *j*, and season *s*, calculated from the nitrogen input and harvested-output terms described above.

Constraints:

1. The total cereal area in each district-season must remain unchanged. This constraint ensures that the optimization reallocates existing cereal cropland rather than expanding or contracting the cultivated area, as shown in equation (10):
  10 $${\sum}_{c\in C}{x}_{i,j,c,s}={\sum}_{c\in C}{A}_{i,j,c,s}^{{{{\rm{cu}}}}}\quad \forall i\in {D}_{j},\,\forall j,\,\forall s\in S$$
2. Crop substitution is limited to cereals historically cultivated within a given district, preserving local agronomic plausibility. Feasible crops are defined by the historical-presence indicator:
  11 $${x}_{i,j,c,s}=0\quad {{{\rm{if}}}}\quad {A}_{i,j,c,s}^{{{{\rm{hs}}}}}=0\quad \forall i\in {D}_{j},\,\forall j,\,\forall c\in C,\,\forall s\in S$$
  No crop-specific historical maximum area constraint is imposed beyond the historical-presence rule, because historical crop area reflects past adoption and procurement conditions rather than a strict future ceiling.
3. Maintaining total calorie production. Total calorie production within each state-season must not fall below the 2017 baseline value, as shown in equation (12). This preserves baseline state-level caloric output as an energy-availability floor during crop restructuring. A nutrient-aware formulation would require additional nutrient-specific targets.
  12 $${\sum}_{i\in {D}_{j}}{\sum}_{c\in C}{{{{\rm{kc}}}}}_{c}\,{x}_{i,j,c,s}\,{Y}_{i,j,c,s}\ge {\sum}_{i\in {D}_{j}}{\sum}_{c\in C}{{{{\rm{kc}}}}}_{c}\,{A}_{i,j,c,s}^{{{{\rm{cu}}}}}\,{Y}_{i,j,c,s}^{{{{\rm{cu}}}}}\quad \forall j,\,\forall s\in S$$
  Here, kc_*c*_ is the crop-specific calorie density from Supplementary Table 4.
4. Benchmarked cereal-net-return stability. State-season net return, evaluated with the net-return coefficient *π*_*j*,*c*_ defined above, must not fall below the 2017 baseline. This preserves a net-return floor under the benchmarked price environment:
  13 $${\sum}_{i\in {D}_{j}}{\sum}_{c\in C}{\pi }_{j,c}\,{x}_{i,j,c,s}\,{Y}_{i,j,c,s}\ge {\sum}_{i\in {D}_{j}}{\sum}_{c\in C}{\pi }_{j,c}\,{A}_{i,j,c,s}^{{{{\rm{cu}}}}}\,{Y}_{i,j,c,s}^{{{{\rm{cu}}}}}\quad \forall j,\,\forall s\in S$$
  Here, *π*_*j*,*c*_ is the state-crop net-return coefficient defined above.

This optimization model, with its specified objective function and constraints, provides a structured methodology for analyzing the impact of crop restructuring on nutrient surplus while adhering to land availability, calorie adequacy, and net-return-preservation requirements.

We also set up a single-objective water-demand minimization model using the same constraints. The objective function is:

14 $$\min \quad {F}_{W}(x)={\sum}_{s\in S}{\sum}_{j}{\sum}_{i\in {D}_{j}}{\sum}_{c\in C}CW{D}_{i,j,c,s}\,{x}_{i,j,c,s}$$

Here, *CWD*_*i*,*j*,*c*,*s*_ denotes the consumptive crop water demand per unit cultivated area. This function minimizes total cereal water demand under the same land, historical-presence, calorie-production, and income-preservation constraints.

The multi-objective optimization framework addresses nitrogen surplus and water demand simultaneously using a weighted objective^78,79^. The combined objective is:

15 $$\min \quad \alpha \frac{{F}_{N}(x)}{{F}_{N}^{2017}}+\left(1-\alpha \right)\frac{{F}_{W}(x)}{{F}_{W}^{2017}}$$

$$\alpha \in [0,1]$$

In this formulation, *α* is a weighting factor ranging from zero to one in 0.01 increments and controls the relative emphasis on minimizing nitrogen surplus versus water demand. ${F}_{N}^{2017}$ and ${F}_{W}^{2017}$ are the corresponding 2017 baseline totals for nitrogen surplus and consumptive water demand, respectively, and are used to normalize the two objective terms before they are combined. A value of *α* closer to one places greater emphasis on nitrogen-surplus reduction, whereas a value closer to zero prioritizes water-demand reduction. For each *α* value, we determined an optimal district-level cropping pattern and calculated national totals of nitrogen surplus and water demand. Plotting these totals gives the Pareto frontier for the Indian cereal system.

The constraints applied to this multi-objective optimization model remain consistent with those detailed above. We also calculate endpoint co-benefits for the two single-objective strategies. To compare nitrogen surplus (kg) with consumptive water demand (m^3^), we use percentage changes relative to the 2017 baseline. For nitrogen-surplus-focused restructuring, the water co-benefit is the percentage reduction in water demand achieved at the nitrogen-focused endpoint. For water-focused restructuring, the nitrogen co-benefit is the percentage reduction in nitrogen surplus achieved at the water-focused endpoint.

Recognizing the indispensable cultural and dietary roles of rice and wheat as staple cereals in India, our optimization model introduces constraints to reflect their importance. These constraints ensure that adjustments to farming patterns aimed at enhancing water efficiency and reducing nitrogen surplus do not disproportionately affect the cultivation of rice and wheat, acknowledging their fundamental place in Indian agriculture and cuisine.

As in the primary optimization framework, these scenarios reallocate crop composition within the baseline district-level cropped area and therefore do not expand or contract total agricultural area. The retained staple-area floor is imposed at the state level, allowing the preserved rice or wheat area to be redistributed among districts within the same state.

We applied this cultural constraint to rice only for the kharif season and to wheat for the rabi season, as these are their primary growing periods in India. The details are as follows:

For rice (r), the constraint ensures that the total rice area retained within each state does not fall below a specified fraction of its current (A^cu^) state total, represented in equation (16):

16 $${\sum}_{i\in {D}_{j}}{x}_{i,j,r,{{{\rm{kharif}}}}}\ge \tau {\sum}_{i\in {D}_{j}}{A}_{i,j,r,{{{\rm{kharif}}}}}^{{{{\rm{cu}}}}}\quad \forall j$$

Similarly equation (17), for wheat (w):

17 $${\sum}_{i\in {D}_{j}}{x}_{i,j,w,{{{\rm{rabi}}}}}\ge \tau {\sum}_{i\in {D}_{j}}{A}_{i,j,w,{{{\rm{rabi}}}}}^{{{{\rm{cu}}}}}\quad \forall j$$

Here, *τ* is a retention parameter ranging from 0 to 1, where *τ* = 1 preserves the baseline state-level staple crop area and smaller values progressively relax that retained-area requirement while allowing within-state redistribution across districts.

18 $$\tau \in [0,1]$$

By incorporating these constraints, the model pragmatically balances sustainability objectives with the cultural imperatives of rice and wheat cultivation in India. This approach underscores the complexity of agricultural optimization in contexts where cultural preferences play an important role in shaping dietary patterns.

### Implications of optimization on various environmental factors

We evaluated the impact of cropland restructuring across various critical dimensions. First, in the environmental dimension, we compared nitrogen surplus and water minimization strategies, examining greenhouse gas (GHG) emissions, nitrogen loss through leaching/runoff and emissions, phosphorus application, and surplus generation. Second, in the social dimension, we analyzed how the trade network would evolve and be impacted by cropland restructuring aimed at reducing nitrogen surplus. Finally, we examined the economic dimension by calculating the social cost of mitigating nitrogen pollution if nitrogen surplus-based restructuring were implemented in India, highlighting the potential monetary gains at the national level.

We evaluate the percentage change in total greenhouse gas (GHG) emissions relative to the baseline value. GHG emissions are calculated under two scenarios: one where only nitrogen surplus minimization is focused (*α* = 1) and another where only water consumption minimization is focused (*α* = 0).

District- and crop-specific agricultural greenhouse gas (AGHG) emissions were quantified by integrating three key sources: (i) methane (CH_4_) emissions from rice cultivation under distinct water management regimes, (ii) emissions from open-field burning of crop residues, and (iii) nitrous oxide (N_2_O) emissions derived from nitrogen surplus in croplands.

For each crop *c* in district *i*, the total AGHG emissions were expressed as:

19 $${T}_{{{{{\rm{AGHG}}}}}_{i,c}}={E}_{i,c}^{{{{{\rm{CH}}}}}_{4}}+{E}_{i,c}^{{{{\rm{Burning}}}}}+{E}_{i,c}^{{{{{\rm{N}}}}}_{2}{{{\rm{O}}}}-{{{\rm{surplus}}}}}$$

where each term is expressed in CO_2_-equivalent units (CO_2_eq) per unit cultivated area.

State-level CH_4_ emission factors for rice were estimated by weighting regime-specific emission coefficients (*EF*_*r*_, kg CH_4_ ha^-1^) by the proportion of rice area (*p*_*s*,*r*_) under each water management regime in state *s*:

20 $${E}_{s,{{{\rm{rice}}}}}^{{{{{\rm{CH}}}}}_{4}}={\sum}_{r}\left({p}_{s,r}\times E{F}_{r}\right)$$

Regimes considered included continuous flooding, single aeration, multiple aeration, upland, deepwater, drought-prone, and flood-prone categories. Area shares were obtained from regional literature^80^, and regime-wise emission coefficients were sourced from India’s BUR III report and are summarized in Supplementary Table 8^8^. The resulting CH_4_ emissions were converted to CO_2_eq using the 100-year global warming potential for CH_4_ ($GW{P}_{{{{{\rm{CH}}}}}_{4}}=28$):

21 $${E}_{s,{{{\rm{rice}}}}}^{{{{{\rm{CH}}}}}_{4}-{{{{\rm{CO}}}}}_{2}{{{\rm{eq}}}}}={E}_{s,{{{\rm{rice}}}}}^{{{{{\rm{CH}}}}}_{4}}\times 28$$

The state-level coefficient was then assigned to all rice-producing districts within that state.

Open-field burning emissions for rice, wheat, maize, and millets were estimated following IPCC inventory guidelines and India-specific residue-burning coefficients^81,82^. The calculation incorporated crop-specific residue-to-crop ratios (*RCR*_*c*_), dry matter fractions (*DMF*_*c*_), state-specific fractions of residue burned (*FB*_*s*,*c*_), and the fraction actually oxidized (*FAO* = 0.93). The state-specific *FB*_*s*,*c*_ overrides listed in Supplementary Table 6 follow Jain et al.^82^, while all remaining cereal-state combinations were assigned the default value of 0.10 used in that inventory. Broader residue and GWP constants are summarized in Supplementary Table 7. Methane and nitrous oxide emission factors ($E{F}_{{{{{\rm{CH}}}}}_{4}}=2.7$ kg t^-1^; $E{F}_{{{{{\rm{CO}}}}}_{2}}=1515$ kg t^-1^; $E{F}_{{{{{\rm{N}}}}}_{2}{{{\rm{O}}}}}=0.07$ kg t^−1^) were obtained^81^ and are summarized in Supplementary Table 9, while GWPs were taken as $GW{P}_{{{{{\rm{CO}}}}}_{2}}=1$, $GW{P}_{{{{{\rm{CH}}}}}_{4}}=28$ and $GW{P}_{{{{{\rm{N}}}}}_{2}{{{\rm{O}}}}}=265$^83^. The per-unit production emission factor was calculated as:

22 $$\begin{array}{l}E{F}_{s,c}^{{{{\rm{burning}}}}}=RC{R}_{c}\times DM{F}_{c}\times F{B}_{s,c}\times FAO\\ \times \left(E{F}_{{{{{\rm{CH}}}}}_{4}}\times GW{P}_{{{{{\rm{CH}}}}}_{4}}+E{F}_{{{{{\rm{CO}}}}}_{2}}\times GW{P}_{{{{{\rm{CO}}}}}_{2}}+E{F}_{{{{{\rm{N}}}}}_{2}{{{\rm{O}}}}}\times GW{P}_{{{{{\rm{N}}}}}_{2}{{{\rm{O}}}}}\right)\end{array}$$

Multiplication of $E{F}_{s,c}^{{{{\rm{burning}}}}}$ by district-level production (Area_*i*,*c*_ × Yield_*i*,*c*_) yielded total district-crop residue-burning emissions; dividing by cultivated area gave the corresponding per-hectare intensity where needed.

Nitrogen surplus for each district and crop was computed as the sum of all nitrogen inputs (synthetic fertilizer, manure, biological fixation, atmospheric deposition) minus nitrogen removal via harvested biomass. State-specific emission fractions (${f}_{{{{{\rm{N}}}}}_{2}{{{\rm{O}}}},s}$, %) were derived from the IMAGE-GNM global nutrient model^55^. The resulting N_2_O emissions (kg ha^−1^) were calculated as:

23 $${E}_{i,c}^{{{{{\rm{N}}}}}_{2}{{{\rm{O}}}}-{{{\rm{surplus}}}}}={N}_{{{{{\rm{surplus}}}}}_{i,c}}\times \frac{{f}_{{{{{\rm{N}}}}}_{2}{{{\rm{O}}}},s}}{100}$$

These were converted to CO_2_eq using $GW{P}_{{{{{\rm{N}}}}}_{2}{{{\rm{O}}}}}=265$.

The total AGHG intensity for each district-crop combination was calculated as:

24 $$\begin{array}{l}GH{G}_{i,c}^{{{{{\rm{CO}}}}}_{2}{{{\rm{eq}}}}}={E}_{i,c}^{{{{{\rm{CH}}}}}_{4}-{{{{\rm{CO}}}}}_{2}{{{\rm{eq}}}}}+{E}_{i,c}^{{{{\rm{Burning}}}}-{{{{\rm{CO}}}}}_{2}{{{\rm{eq}}}}}\\+{E}_{i,c}^{{{{{\rm{N}}}}}_{2}{{{\rm{O}}}}-{{{\rm{surplus}}}}-{{{{\rm{CO}}}}}_{2}{{{\rm{eq}}}}}\end{array}$$

This framework was applied annually over the study period using year-specific data for crop areas, yields, nitrogen surplus, and water management regime shares where available. The 2017 district-level, crop-specific AGHG intensities (Mg CO_2_eq ha^−1^) were subsequently used for the baseline optimization and scenario comparisons.

We calculated the percentage change in nitrogen lost to the environment through leaching/runoff losses (${{{{\rm{NO}}}}}_{3}^{-}$) and nitrous oxide loss (N_2_O-N) relative to baseline values. This nitrogen loss to the environment was calculated under two endpoint scenarios: nitrogen-surplus minimization (*α* = 1) and water-demand minimization (*α* = 0). Nitrogen loss was estimated as a fixed fraction of nitrogen surplus (NS), following methods from previous literature^38,84^. The loss fractions were derived using the dynamic IMAGE-GNM model^55^ (Supplementary Fig. S15). Nitrous oxide loss was quantified using equation (25):

25 $${N}_{{{{\rm{N}}}}}2{{{\rm{O}}}}-{{{\rm{N}}}}={\sum}_{j}N{S}_{j}\,E{F}_{j}$$

Here, *N*_N_2O − N represents total national N_2_O-N loss from surplus nitrogen in managed soils (kg). The emission factor for state *j* (*EF*_*j*_) was calculated using IMAGE-GNM model data^13^. Aquatic nitrogen loss through leaching and runoff was calculated as:

26 $$\begin{array}{r}{N}_{{{{\rm{leach}}}}}={\sum}_{j}N{S}_{j}\,L{F}_{j}\end{array}$$

In this equation, *N*_leach_ represents total nitrogen lost to aquatic systems through leaching and runoff. The leaching fraction for state *j* (*LF*_*j*_) represents the fraction of nitrogen surplus in managed soils that is lost by leaching and runoff. These coefficients were derived using spatially explicit data from the IMAGE-GNM model^13^.

We investigated the consequences of nitrogen-surplus-reduction-based crop restructuring on Indian interstate agricultural trade networks at the state level^85^. District-level optimized crop production was first aggregated to states and then linked to the interstate trade network. We used trade-network data from Kulkarni et al.^57^ and Goyal et al.^13^, focusing on the three-year average trade volumes around the 2017 baseline. These data include crop-specific volumes in tonnes for rice and wheat, and aggregate volumes for maize, bajra, jowar, and ragi, which we collectively refer to as alternative cereals. States are represented as nodes, direct exchanges as edges, and edge weights as crop or crop-group trade volumes.

The restructured trade network was calculated from an exporter-centric perspective. For each staple crop *k* ∈ {rice, wheat}, the baseline export ratio for exporting state *i* was calculated as *ρ*_*i*,*k*_ = *W*_*i*,*k*,2017_/*P*_*i*,*k*,2017_, where *W*_*i*,*k*,2017_ is total baseline exports and *P*_*i*,*k*,2017_ is baseline production. If optimization changed production to *P*_*i*,*k*,opt_, the optimized export volume was *W*_*i*,*k*,opt_ = *ρ*_*i*,*k*_*P*_*i*,*k*,opt_, with link-level exports scaled by the same exporter-specific ratio. When reduced rice and wheat exports created a calorie deficit on a trade link from exporter *i* to importer *j*, the staple-calorie deficit was calculated as:

27 $$\begin{array}{l}{D}_{ij}^{{{{\rm{staple}}}}}=\left({W}_{ij,{{{\rm{rice}}}},2017}-{W}_{ij,{{{\rm{rice}}}},{{{\rm{opt}}}}}\right){{{{\rm{kc}}}}}_{{{{\rm{rice}}}}}\\+\left({W}_{ij,{{{\rm{wheat}}}},2017}-{W}_{ij,{{{\rm{wheat}}}},{{{\rm{opt}}}}}\right){{{{\rm{kc}}}}}_{{{{\rm{wheat}}}}}\end{array}$$

where *i* is the exporting state, *j* is the importing state, *W* is expressed in tonnes, and kc is the crop-specific calorie density.

For links where optimized staple trade exceeded baseline, ${D}_{ij}^{{{{\rm{staple}}}}}$ was set to zero for the alternative-cereal reconstruction. To compensate for positive staple-calorie deficits, alternative cereals (maize, bajra, jowar, and ragi) were allocated on the union of baseline alternative-cereal links and exporter-importer pairs with positive ${D}_{ij}^{{{{\rm{staple}}}}}$, expressed in kcal. Link strengths for alternative cereals were updated as:

28 $${W}_{ij,{{{\rm{alt}}}},{{{\rm{opt}}}}}^{{{{\rm{kcal}}}}}={W}_{ij,{{{\rm{alt}}}},2017}^{{{{\rm{kcal}}}}}+{D}_{ij}^{{{{\rm{staple}}}}}$$

subject to the exporter production-capacity constraint:

29 $${\sum}_{j=1}^{n}{W}_{ij,{{{\rm{alt}}}},{{{\rm{opt}}}}}^{{{{\rm{kcal}}}}}\le {P}_{i,{{{\rm{alt}}}},{{{\rm{opt}}}}}^{{{{\rm{kcal}}}}}$$

where ${W}_{ij,{{{\rm{alt}}}},2017}^{{{{\rm{kcal}}}}}$ is the baseline alternative-cereal trade volume converted to kcal and ${P}_{i,{{{\rm{alt}}}},{{{\rm{opt}}}}}^{{{{\rm{kcal}}}}}$ is optimized alternative-cereal production in exporting state *i*, also expressed in kcal. If a positive staple-deficit link had no baseline alternative-cereal flow, Eq. (28) introduced a new alternative-cereal link on that exporter-importer pair.

This ensures that lost staple calories are compensated on the modeled trade links, while the optimization itself separately enforces state-level calorie and benchmarked-income constraints. The trade-network reconstruction is formulated as a calorie-preserving redistribution exercise; it is not an independent market-equilibrium model for realized export revenue.

We translated the observed reductions in N leaching (${{{{\rm{NO}}}}}_{3}^{-}$) and nitrous oxide (N_2_O) emissions (see eqs. (26), (25)) relative to the baseline year into monetary terms by applying per-kilogram damage costs (USD kg^−1^ N) for nitrogen losses to water and air, capturing health, ecosystem, and climate damages. All monetary values are reported in constant 2015 USD. Unit costs were taken from Sutton et al.^56^ (Supplementary Table 5), converted from INR to USD using the World Bank average official exchange rate for 2015^86^, and, where necessary, adjusted to 2015 prices using the World Bank GDP deflator. Costs were scaled to India’s 2015 gross national income^87^.

### Sensitivity and uncertainty analysis

We quantified uncertainty in nutrient surplus and loss estimates derived from fertilizer and manure application data from the Cost of Cultivation survey (2009–2019) for six major crops across all districts of India^64^. To propagate uncertainty from farmer-reported input use, we implemented nonparametric bootstrapping with 1000 iterations, resampling district-level observations by crop and year. The resulting empirical confidence intervals were propagated to subsequent socio-environmental outcome assessments, including benchmarked cereal net return, agricultural greenhouse-gas (AGHG) emissions, water use, and nitrogen balances, thereby explicitly incorporating input variability into modeled outcomes. For the combined endpoint comparison shown in Fig. 2b, we additionally propagated district-input uncertainty through 500 bootstrap realizations of water-demand and net-nitrogen coefficients, re-solved the two endpoint strategies, and recalculated all ten displayed socio-environmental metrics. Figure 2b reports the national endpoint value together with the 2.5th–97.5th percentile interval of this bootstrap distribution for each displayed metric; the fixed-allocation frontier bootstrap design used for Supplementary Fig. S19 is described in Supplementary Methods 1. Where data availability was limited, or input parameters originated from heterogeneous sources, we performed one-at-a-time sensitivity analyses by perturbing baseline values by  ± 10% for benchmarked cereal net return, AGHG emissions, consumptive water use, and nitrogen surplus. Because nitrogen surplus comprises multiple components, including biological nitrogen fixation, atmospheric deposition, manure application, and synthetic fertilizer use, we also varied each component independently by  ± 10% to assess their individual influence under data constraints and to identify the relative contribution of each pathway to overall surplus variability. Sensitivity and uncertainty results are reported alongside baseline estimates in Fig. 2b and Supplementary Figs. S7–S11, S19.

### Data management and analysis

Data assembly and preprocessing were conducted in Excel 2016 (Microsoft) and Python. Linear programs were solved with the PuLP Python library. Figures and post-optimization summaries were generated in Python and Origin.

### Reporting summary

Further information on research design is available in the Nature Portfolio Reporting Summary linked to this article.

## Supplementary information

Supplementary Information

Reporting Summary

Transparent Peer Review file

## Data Availability

The minimum dataset required to interpret, verify, and extend the findings of this study is described in Supplementary Table 1 and comprises the public input datasets, the accompanying Source Data workbook, and the processed optimization-output tables generated during the analysis. Public input datasets were obtained from the Directorate of Economics and Statistics, Department of Agriculture and Farmers Welfare, Government of India, including district-level crop yields and harvested area from the Crops APY Report Web^66^, available at https://data.desagri.gov.in/website/crops-apy-report-web; plot-wise Cost of Cultivation survey summaries^67^, available at https://desagri.gov.in/document-report-category/plot-wise-summary-data/; and the Manual on Cost of Cultivation Surveys^68^, available at https://desagri.gov.in/wp-content/uploads/2021/06/manual_cost_cultivation_surveys_23july08_0.pdf. Administrative boundary geometries used for map visualization were obtained from Guneet Narula’s *indian-district-boundaries* repository (https://github.com/guneetnarula/indian-district-boundaries), which provides Government of India 2019 district boundaries for visualization and is released under the MIT License. Interstate agricultural trade statistics were obtained from the Directorate General of Commercial Intelligence and Statistics, Ministry of Commerce and Industry, Government of India^85^, available at http://www.dgciskol.gov.in/. Calorie and nutrient composition data were taken from the Indian Food Composition Tables, National Institute of Nutrition, Indian Council of Medical Research^88^, available at https://www.nin.res.in/ebooks/IFCT2017.pdf. State-level crop cost-of-production benchmarks and minimum support price reference tables were obtained from *Agricultural Statistics at a Glance 2017*^64^, available at https://desagri.gov.in/wp-content/uploads/2021/04/Agricultural-Statistics-at-a-Glance-2017.pdf. State-year crop value of output data used to derive the primary 2017–18 realized-price benchmark and supporting diagnostics were obtained from the Ministry of Statistics and Program Implementation, Government of India^89^, available at https://www.mospi.gov.in/publications-reports/innerpage/431. State-year and state-year-season cereal production data used to derive the primary revenue benchmark and seasonal summaries were obtained through the DES APY/UPAg public report route, including the Unified Portal for Agricultural Statistics, Government of India^66,90^, available at https://upag.gov.in/. Source Data for Figs. 1–3, Supplementary Figs. S2–S5 and S16–S20 are provided with this paper as a separate workbook. Shareable processed analysis-ready tables, source-data exports, and the reproducibility workflow are archived at Zenodo (10.5281/zenodo.19684339) and mirrored at https://github.com/udit1408/india-cereal-restructuring-reproducibility.

## Code availability

The code used for data processing, optimization, uncertainty analyses, and figure generation is archived at Zenodo (10.5281/zenodo.19684339) and mirrored at https://github.com/udit1408/india-cereal-restructuring-reproducibility.
