# Supplementary material for: Quantifying environmental co-benefits of nitrogen-based crop restructuring and its implications on India’s interstate trade network
Source: Nat Commun. 2026 Aug 4;17:7778. doi: 10.1038/s41467-026-75905-w (PMC13438110; doi:10.1038/s41467-026-75905-w)
Supplement: Supplementary file 1 [file 41467_2026_75905_MOESM1_ESM.pdf]

Supplementary information

Supplementary Information for: Quantifying Environmental Co-Benefits of Nitrogen-Based Crop Restructuring and Its Implications on India's Interstate Trade Network.

Shekhar Sharan Goyal[1], *Rohini Kumar*[2*], *Udit Bhatia*[3,4*]

[1]Department of Earth Sciences, Indian Institute of Technology Gandhinagar, Palaj, Gandhinagar, 382055, Gujarat, India
[3]Department of Civil Engineering, Indian Institute of Technology Gandhinagar, Palaj, Gandhinagar, 382055, Gujarat, India
[4]Department of Computer Science Engineering, Indian Institute of Technology Gandhinagar, Palaj, Gandhinagar, 382055, Gujarat, India
[2]Computational Hydrosystems, Helmholtz Center for Environmental Research, UFZ, Leipzig, Germany

*Correspondence to: rohini.kumar@ufz.de, bhatia.u@iitgn.ac.in

This file includes:

Supplementary Methods 1 to 3

Supplementary Discussion 1

Supplementary Figures S1 to S20

Supplementary Tables 1 to 12

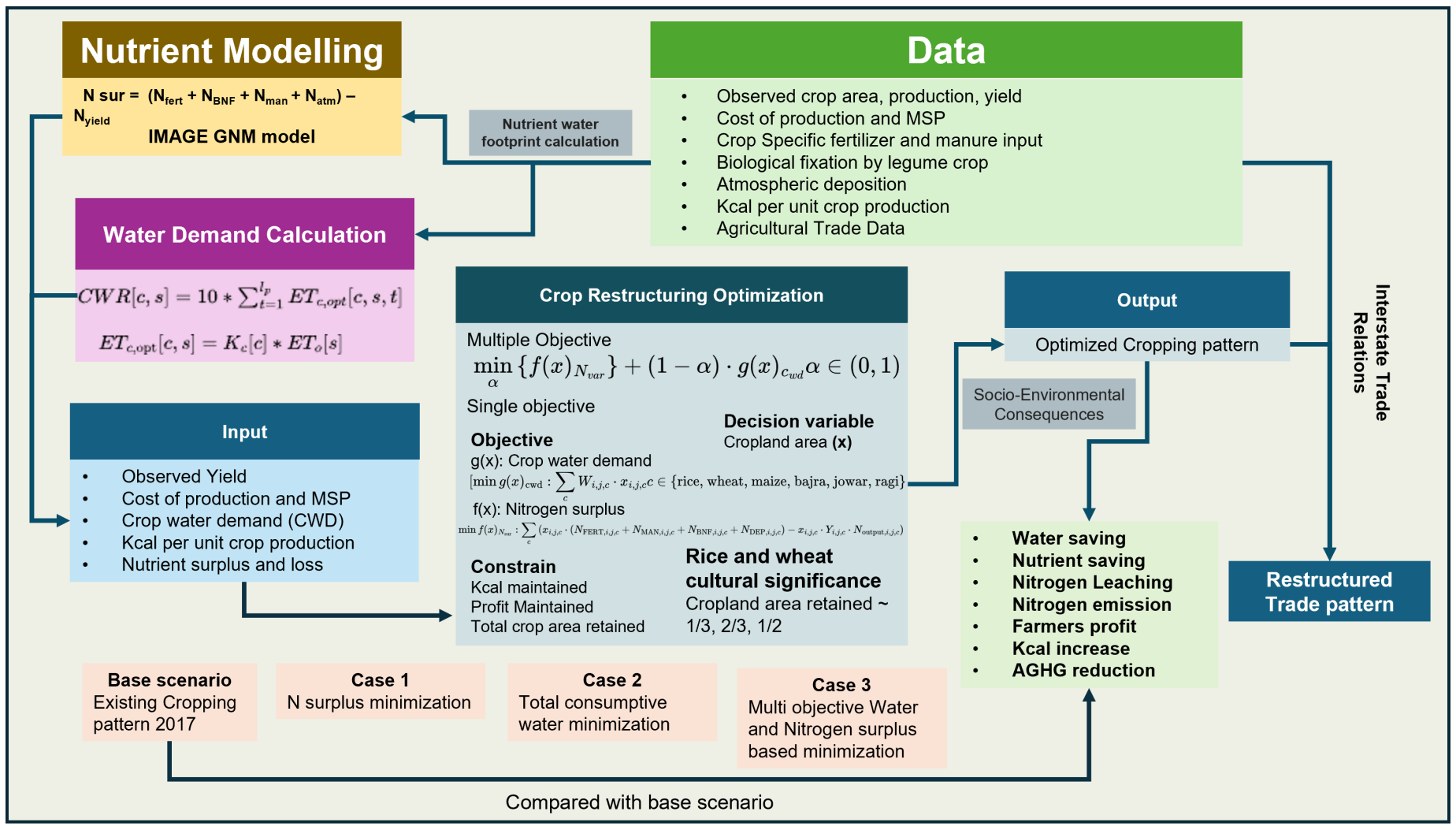

Supplementary Figure 1 – Methodological framework. The figure illustrates the overall workflow used in this study, linking data inputs, optimization procedures, and output indicators. It summarizes the sequence of analytical steps used to evaluate agricultural restructuring scenarios and their associated environmental and economic outcomes.

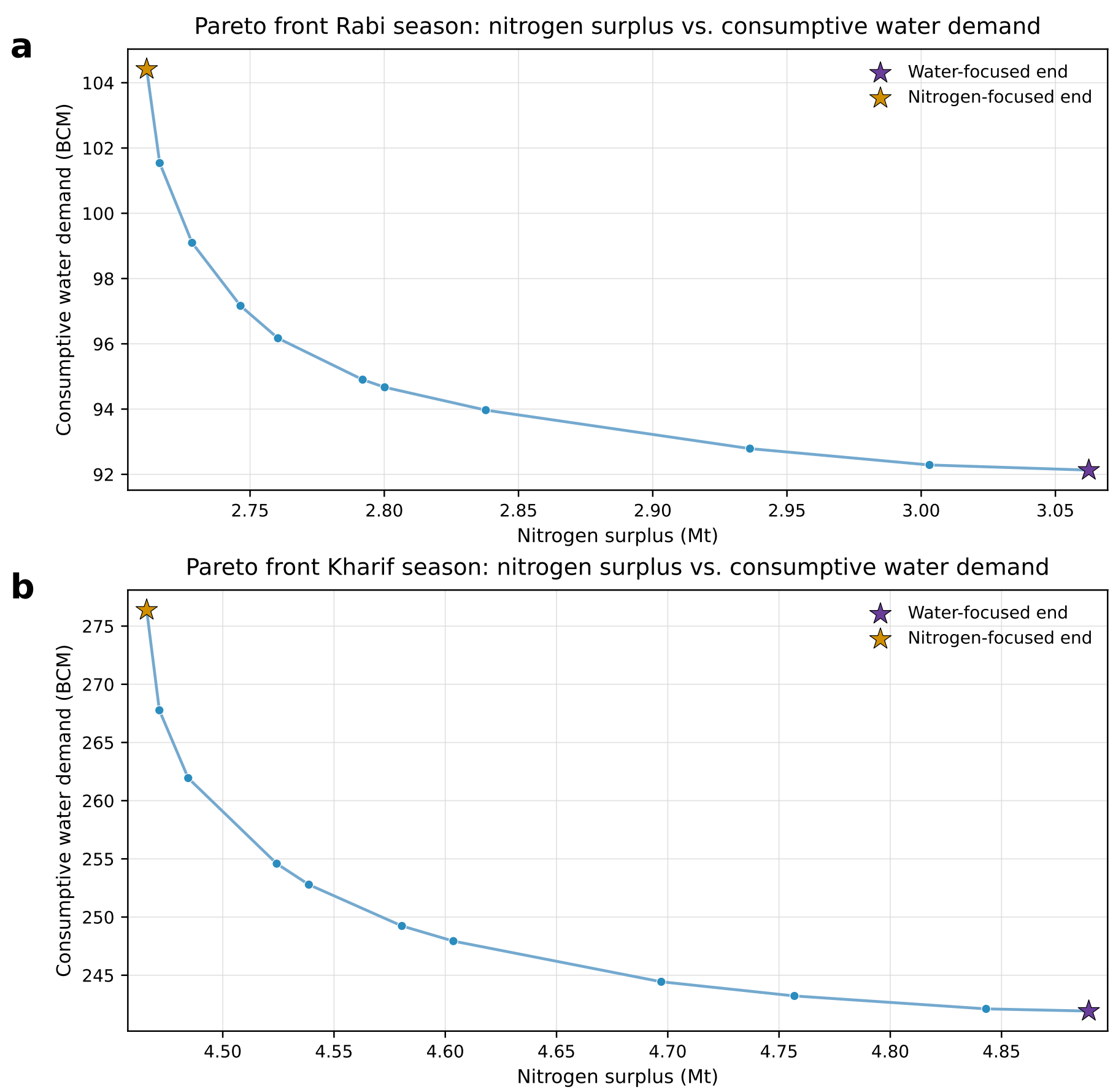

Supplementary Figure 2 – Comparing Water and Nitrogen Pareto Curves in Rabi and Kharif Seasons:(a) Rabi Season (b) Kharif Season. Results in Supplementary Figs. S2–S4 use the primary 2017–18 revenue benchmark used in the main text; district-MSP comparison figures are provided later in the Supplementary Information.

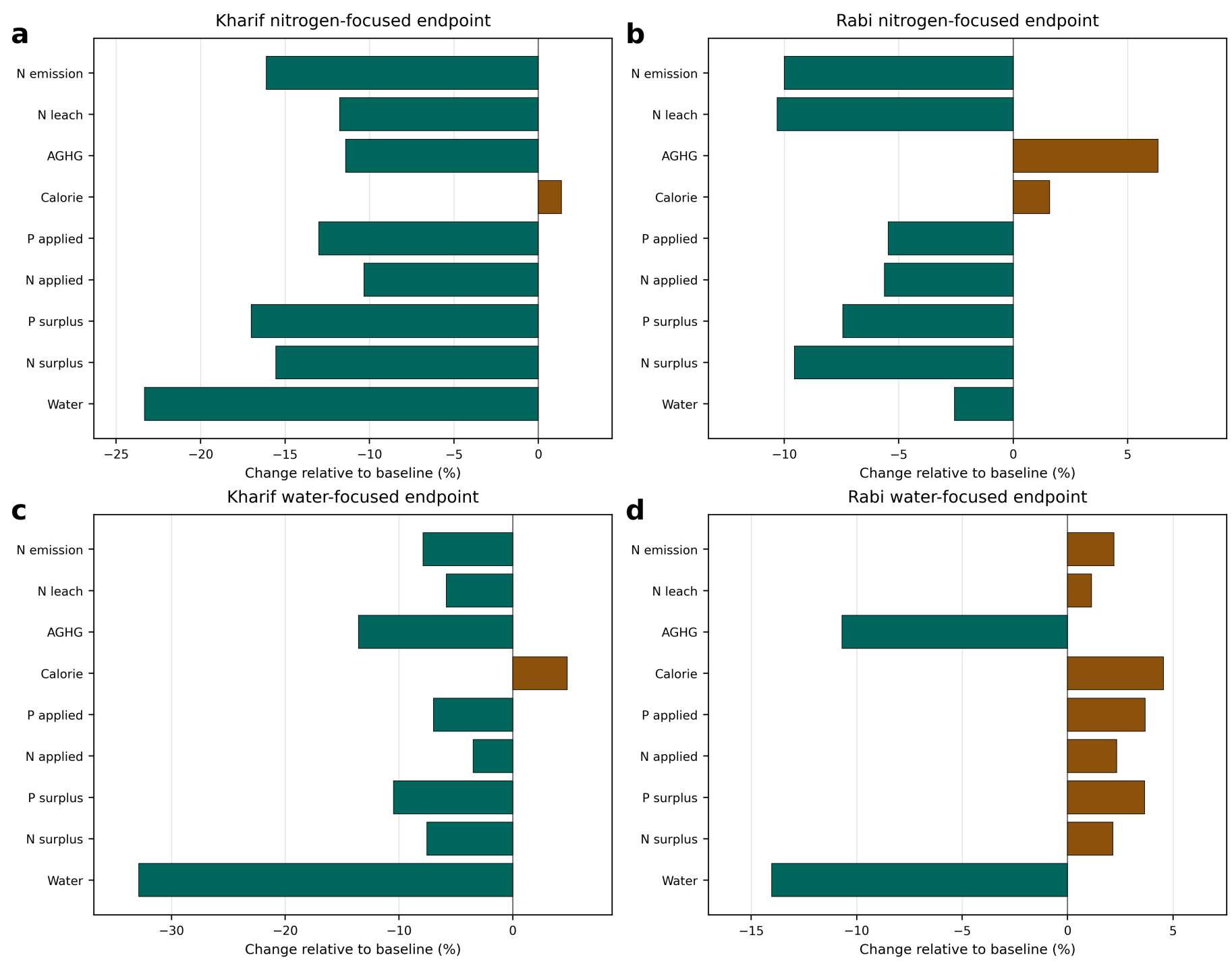

Supplementary Figure 3 – Analyzing Percentage Change Metrics for Socio-Environmental Tradeoffs: (a) and (c) depict nitrogen-focused and water-focused endpoints in the kharif season, while (b) and (d) show the corresponding rabi endpoints. Results use the primary 2017–18 revenue benchmark used in the main text. The seasonal percentage panels omit the benchmarked economic term because kharif starts from a positive benchmarked net return whereas rabi starts from a benchmarked deficit, making direct percentage comparison across seasons visually misleading. Under the nitrogen-focused endpoint, kharif benchmarked net return rises from 8.4 to 29.7 billion rupees, while the rabi deficit narrows from 169.5 to 165.3 billion rupees; under the water-focused endpoint, the kharif benchmark is essentially unchanged and the rabi deficit narrows to 138.2 billion rupees.

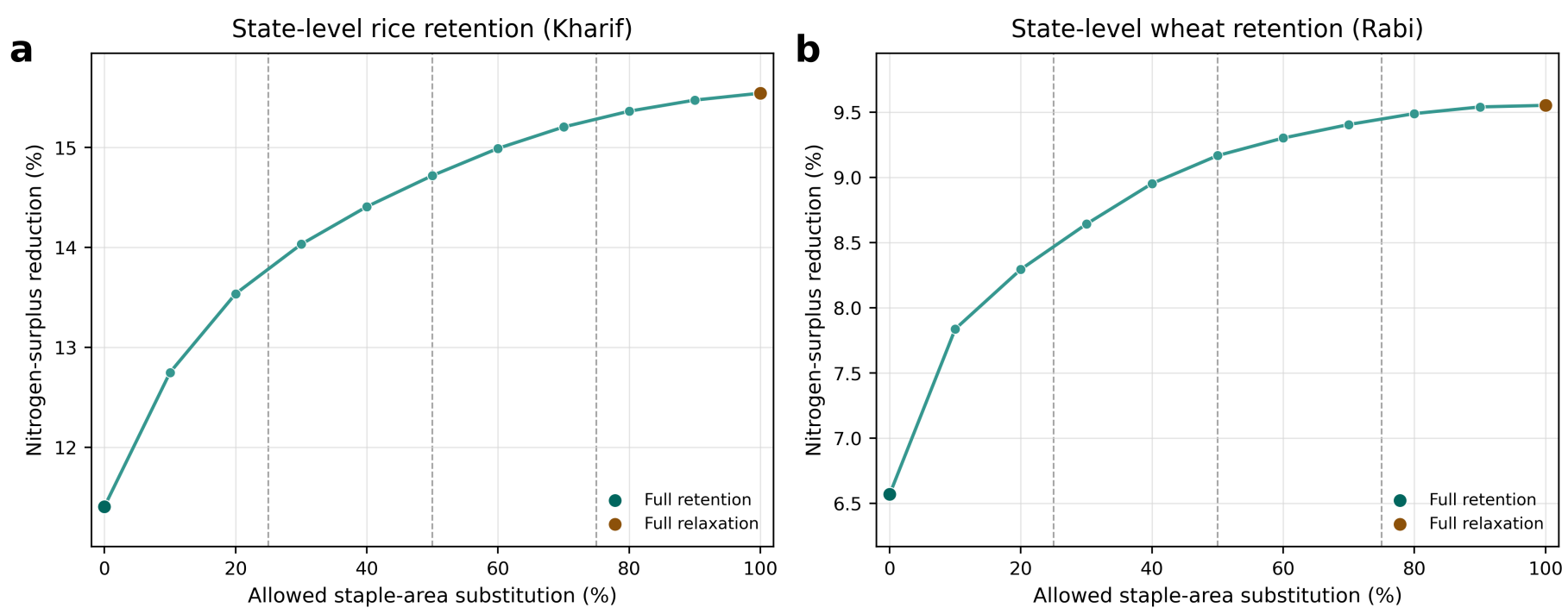

Supplementary Figure 4 – Exploring the Cultural Significance of Staple Crops in Kharif and Rabi Seasons on Nitrogen Surplus Reduction Potential: Seasonal responses of nitrogen-surplus reduction to progressively relaxed state-level retention of rice in the Kharif season (a) and wheat in the Rabi season (b). Results use the primary 2017–18 revenue benchmark used in the main text.

Supplementary Table 1 – Datasets used in this study. Exact landing links to the original public sources are embedded in the Source column wherever available.

Data type	Source	Resolution	Ref
**Yield, harvested area, and derived crop production (optimization baseline)**	Department of Agriculture and Farmers Welfare DES crop statistics (DES APY portal)	District-level	[2]
**Crop cost of production**	Agricultural Statistics at a Glance 2017 (official PDF)	State-level	[3]
**MSP**	Government of India Farmers Portal (MSP statements)	India-level	[4]
**Calorie and nutritional content**	National Institute of Nutrition (IFCT 2017; RDA page)	India-level	[5, 6]
**Fertilizer and manure application**	DES plot summaries (official landing page)	Plot-level	[7]
**N atmospheric deposition**	input4MIPS (ESGF search portal)	Gridded	[8]
**Biological nitrogen fixation benchmark**	FAOSTAT (FAOSTAT data portal)	India-level benchmark	[9]
**N and P Content**	FAOSTAT (FAOSTAT data portal)	Global	[9]
**Agricultural greenhouse-gas benchmark**	BUR-3 Tier III dataset (methodology note)	District-level	[10]
**Crop water demand**	Kampman et al. (original report landing page)	State-level	[11]
**Agricultural trade**	Ministry of Commerce and Industry DGCIS (public trade dashboard)	State-level	[12]
**N loss**	IMAGE-GNM (model paper and links)	$0.5° \times 0.5°$	[13]
**State-year crop value of output (primary revenue benchmark)**	MoSPI Statistical Report on Value of Output from Agriculture and Allied Sectors (publication page)	State-year	[14]
**State-year and state-year-season cereal production (primary benchmark and seasonal disaggregation)**	DES APY public report route (UPAg portal; DES APY portal)	State-year; state-year-season	[2, 15]
**Derived realized-price benchmark tables**	Constructed in this study from MoSPI value-of-output and DES APY production data	State-crop-year; crop-year	This study

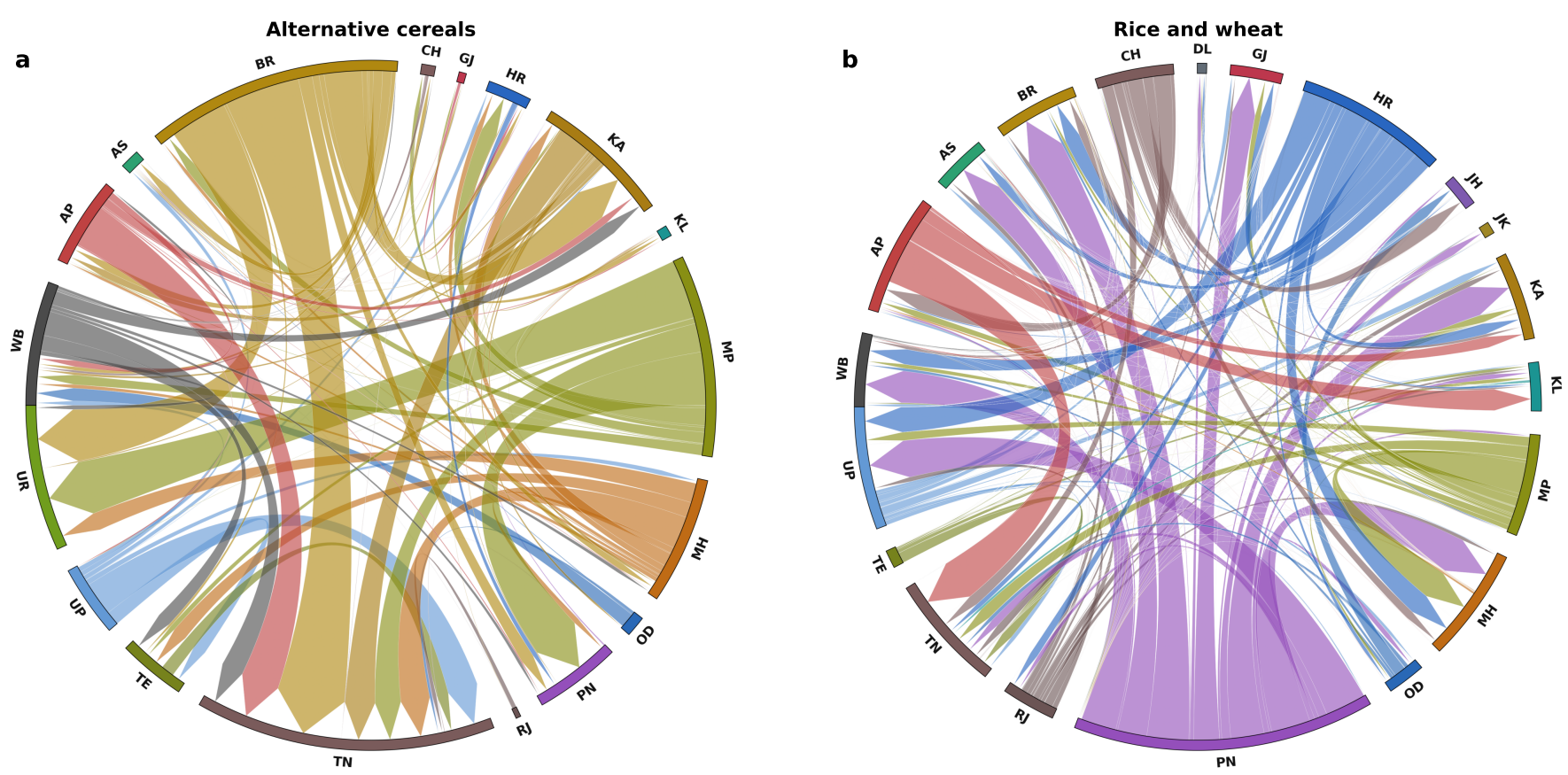

Supplementary Figure 5 – Observed interstate cereal trade network, 2016–2018. **(a)** Average interstate trade of combined alternative cereals, including maize, ragi, jowar, and bajra, expressed in kilocalories. **(b)** Average interstate trade of combined rice and wheat, expressed in kilocalories. In both panels, arrows indicate flow from exporting to importing states, link colour denotes the exporting state, and link width is proportional to average trade volume. State abbreviations are shown for the states with the largest total incoming and outgoing trade volumes.

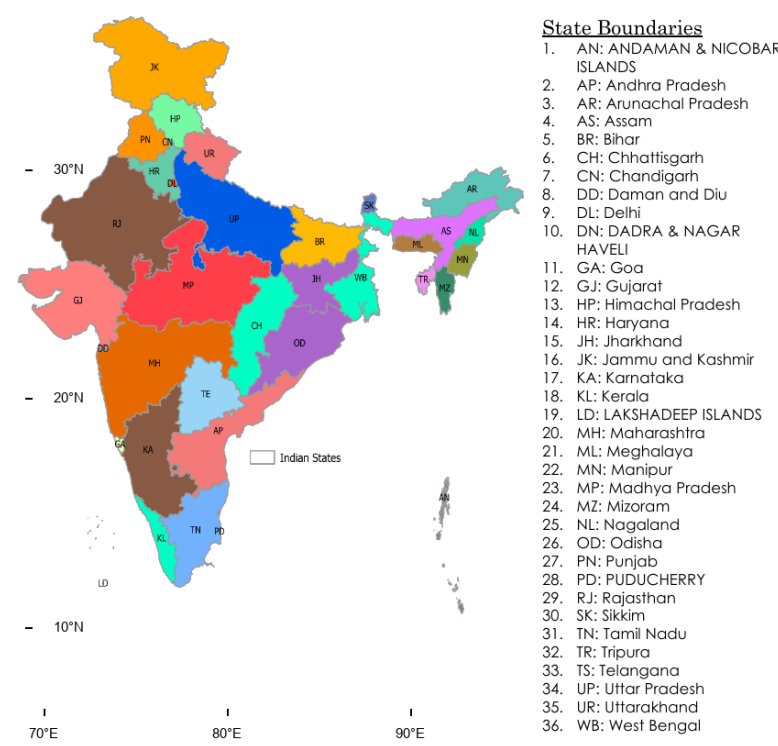

Supplementary Figure 6 – Map of Indian state boundaries used for spatial summaries. The figure shows the state boundaries used throughout the analysis for aggregating and reporting spatially explicit results. These administrative boundaries provide the geographic framework for state-level summaries of crop production, trade flows, and socio-environmental indicators. State boundary geometries used for map visualization were obtained from the *indian-district-boundaries* GitHub repository, released under the MIT License; Copyright (c) 2020 Guneet Narula [1].

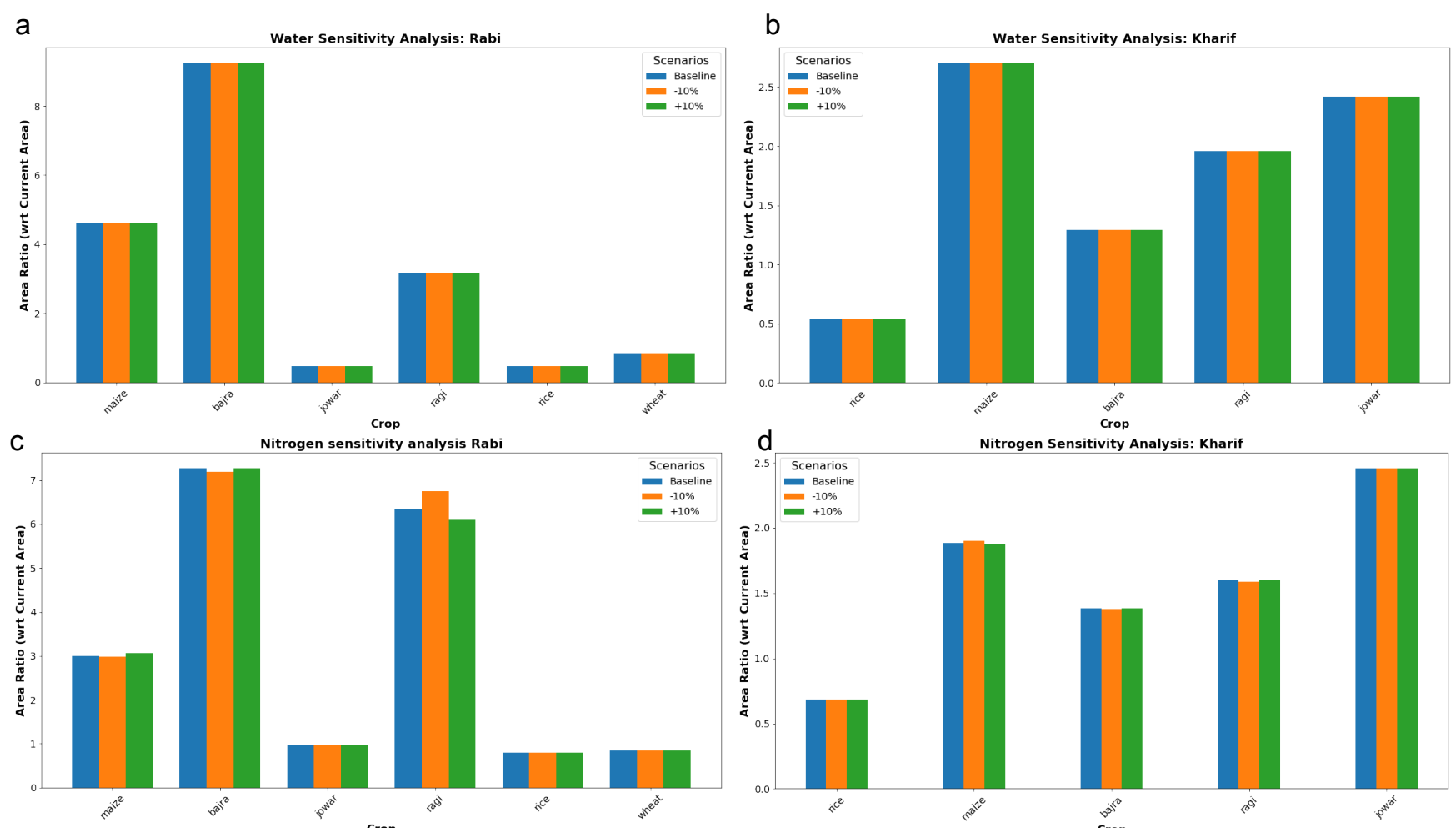

Supplementary Figure 7 – Post-optimization sensitivity analysis of crop-area fractions. Panels show optimum crop-area fractions relative to the original cropping area under ±10% perturbations of key inputs: **(a)** water-demand perturbation in rabi, **(b)** water-demand perturbation in kharif, **(c)** nitrogen-input perturbation in rabi, and **(d)** nitrogen-input perturbation in kharif.

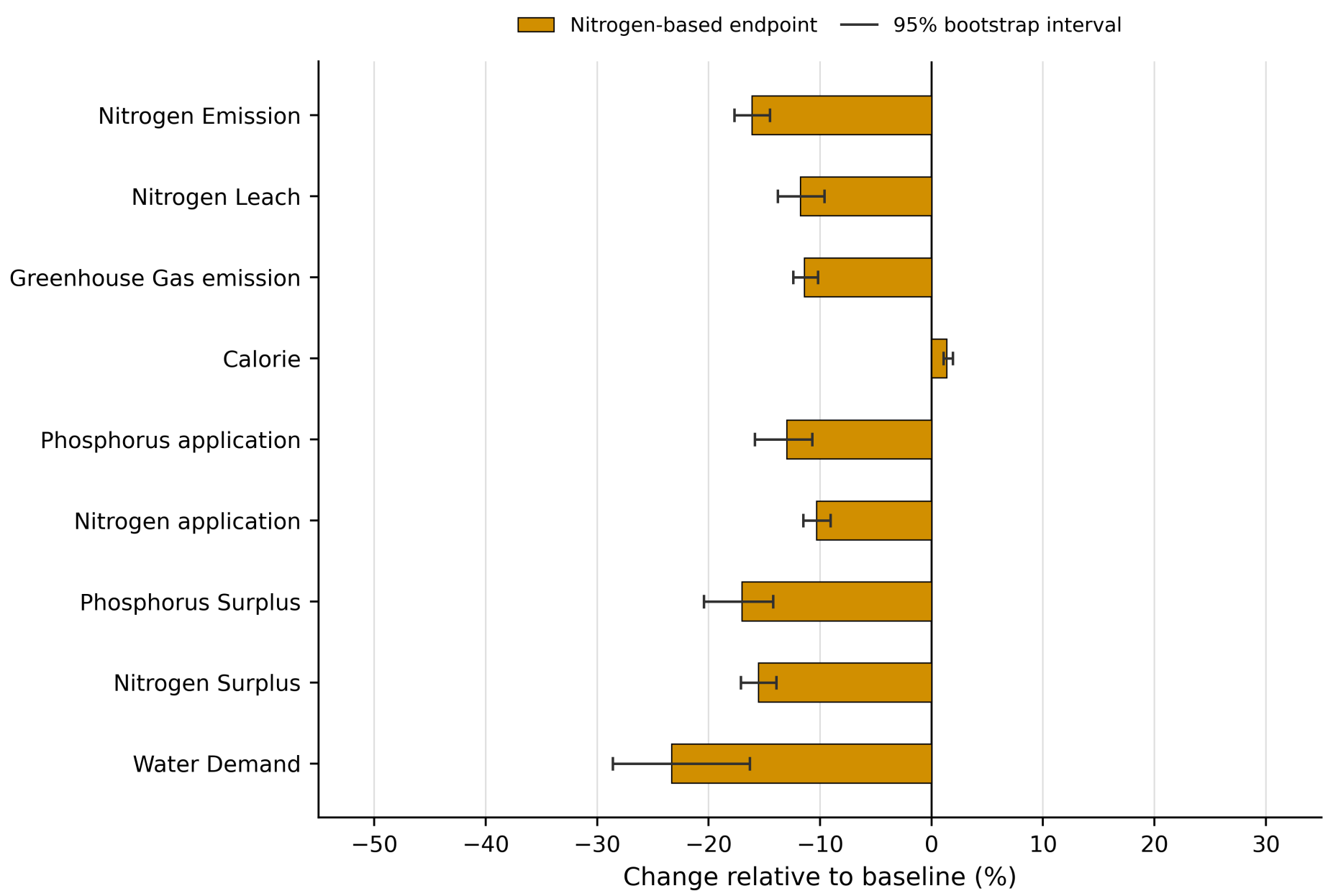

Supplementary Figure 8 – Seasonal uncertainty around the kharif nitrogen-focused endpoint. Bars report deterministic percentage changes relative to the kharif baseline under the primary 2017–18 realized-price and C2 cost benchmark. The benchmarked economic term is omitted from this seasonal percentage panel because kharif starts from a positive benchmarked net return, whereas the paired rabi analysis starts from a benchmarked deficit, so a common percentage scale would be misleading. Horizontal whiskers show 95% bootstrap intervals (97.5–2.5 percentiles) derived from n=500 resampled district-level coefficient fields for consumptive water demand and net nutrient application rates.

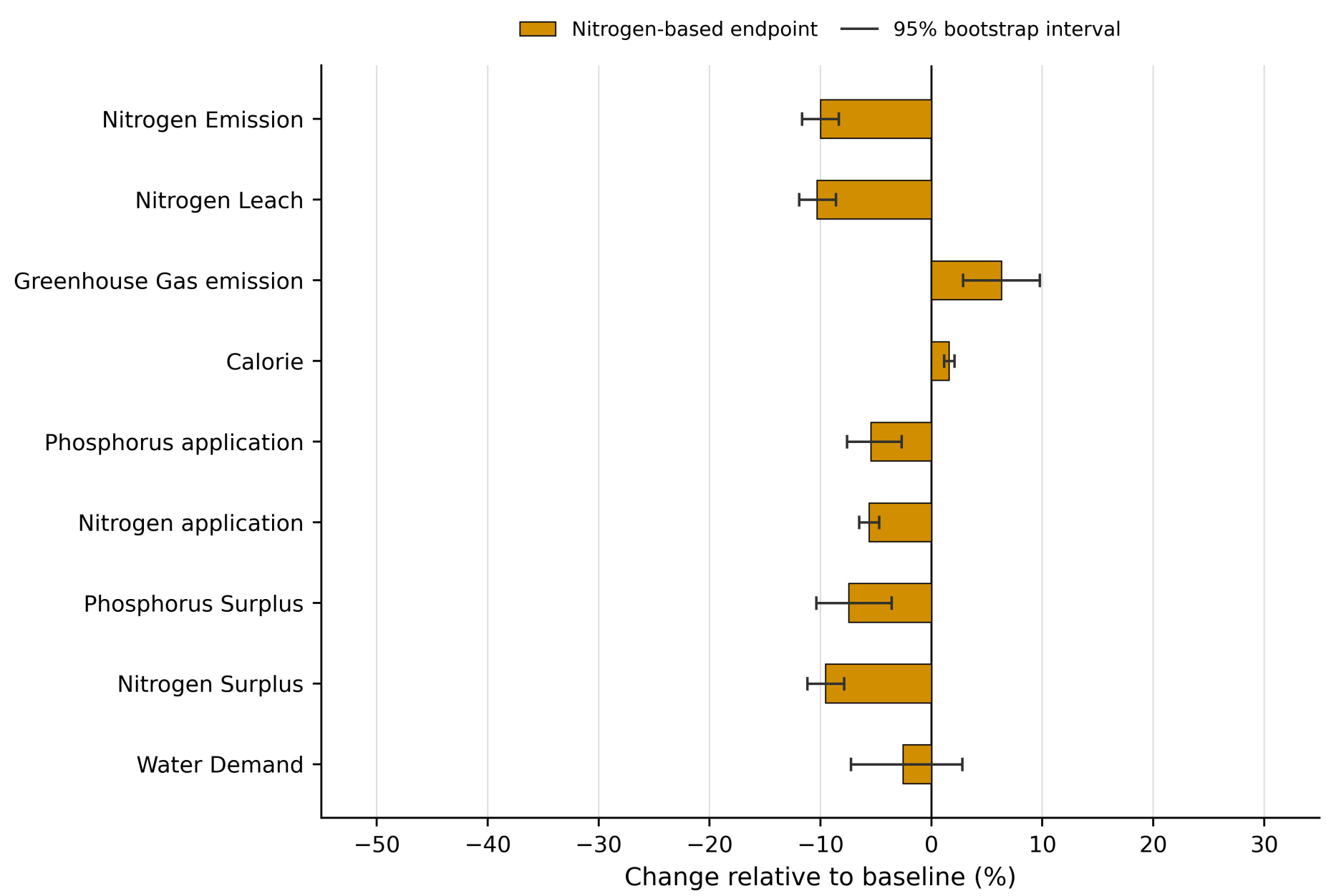

Supplementary Figure 9 – Seasonal uncertainty around the rabi nitrogen-focused endpoint. Bars report deterministic percentage changes relative to the rabi baseline under the primary 2017–18 realized-price and C2 cost benchmark. The benchmarked economic term is omitted from this seasonal percentage panel because rabi starts from a benchmarked deficit, whereas the paired kharif analysis starts from a positive benchmarked net return, so a common percentage scale would be misleading. Horizontal whiskers show 95% bootstrap intervals (97.5–2.5 percentiles) derived from n=500 resampled district-level coefficient fields for consumptive water demand and net nutrient application rates.

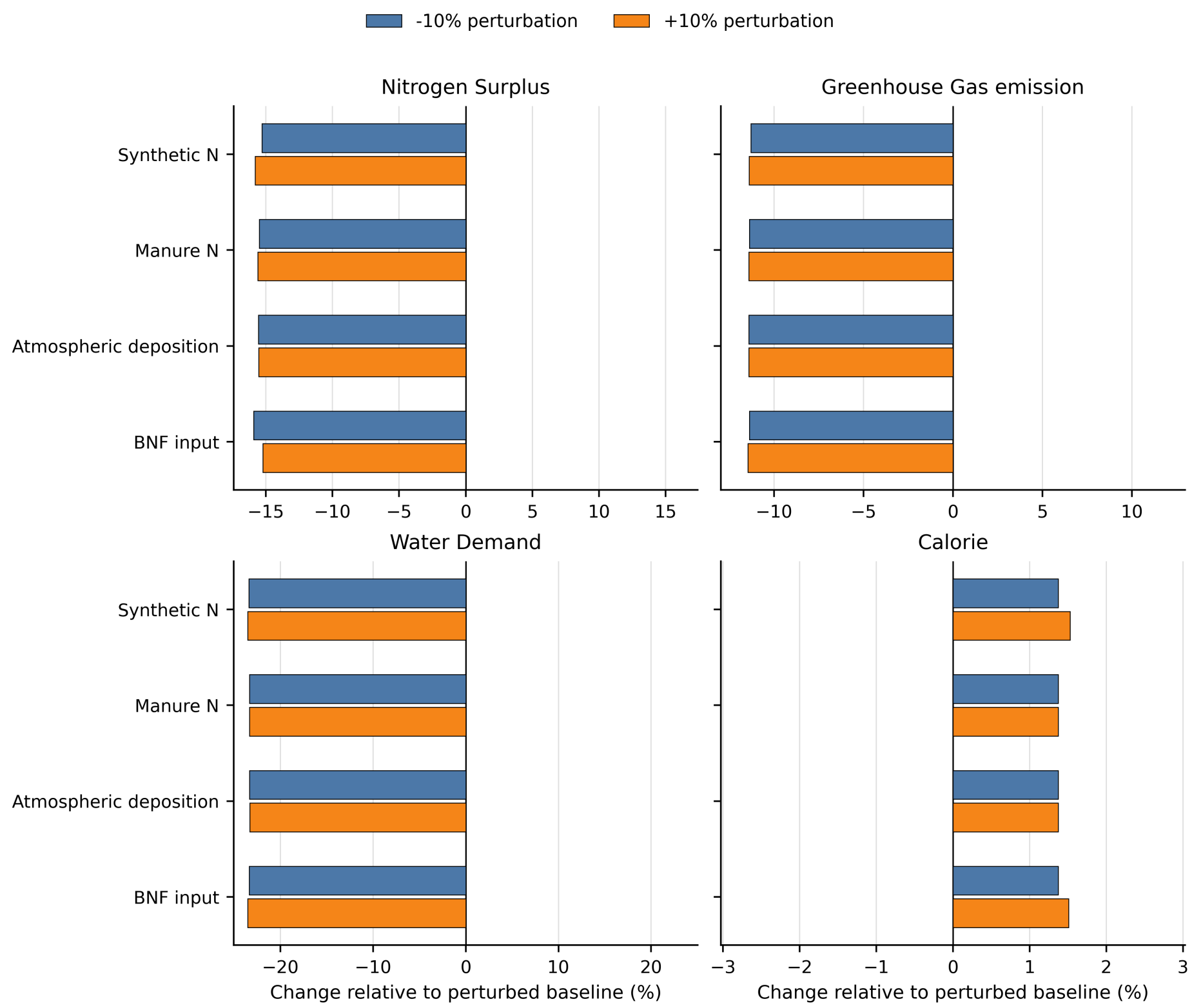

Supplementary Figure 10 – One-at-a-time nitrogen-component sensitivity of the kharif nitrogen-focused endpoint. Panels report percentage changes relative to the corresponding perturbed kharif baseline under the primary 2017–18 realized-price and C2 cost benchmark. The four perturbation groups are synthetic fertilizer nitrogen application, manure nitrogen, atmospheric nitrogen deposition, and the exogenous BNF input term used in the nitrogen balance; each is varied independently by ±10% while all other coefficients are held fixed. The displayed outcomes are nitrogen surplus, agricultural greenhouse gas emissions, water demand, and calorie output. The seasonal economic term is omitted because kharif starts from a positive benchmarked net return whereas rabi starts from a benchmarked deficit, so a common seasonal percentage scale is not directly comparable.

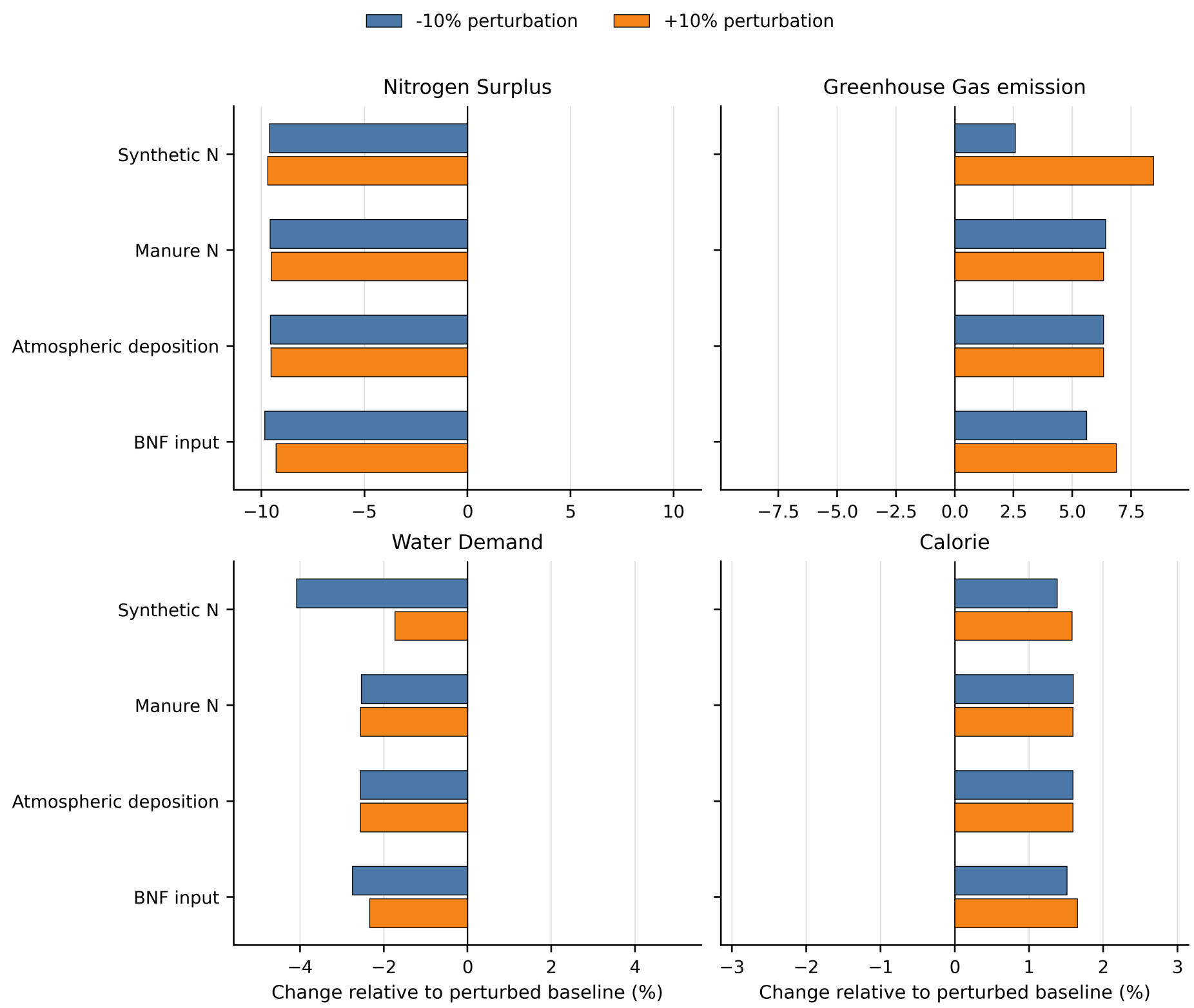

Supplementary Figure 11 – One-at-a-time nitrogen-component sensitivity of the rabi nitrogen-focused endpoint. Panels report percentage changes relative to the corresponding perturbed rabi baseline under the primary 2017–18 realized-price and C2 cost benchmark. The four perturbation groups are synthetic fertilizer nitrogen application, manure nitrogen, atmospheric nitrogen deposition, and the exogenous BNF input term used in the nitrogen balance; each is varied independently by ±10% while all other coefficients are held fixed. The displayed outcomes are nitrogen surplus, agricultural greenhouse gas emissions, water demand, and calorie output. The seasonal economic term is omitted because rabi starts from a benchmarked deficit whereas kharif starts from a positive benchmarked net return, so a common seasonal percentage scale is not directly comparable.

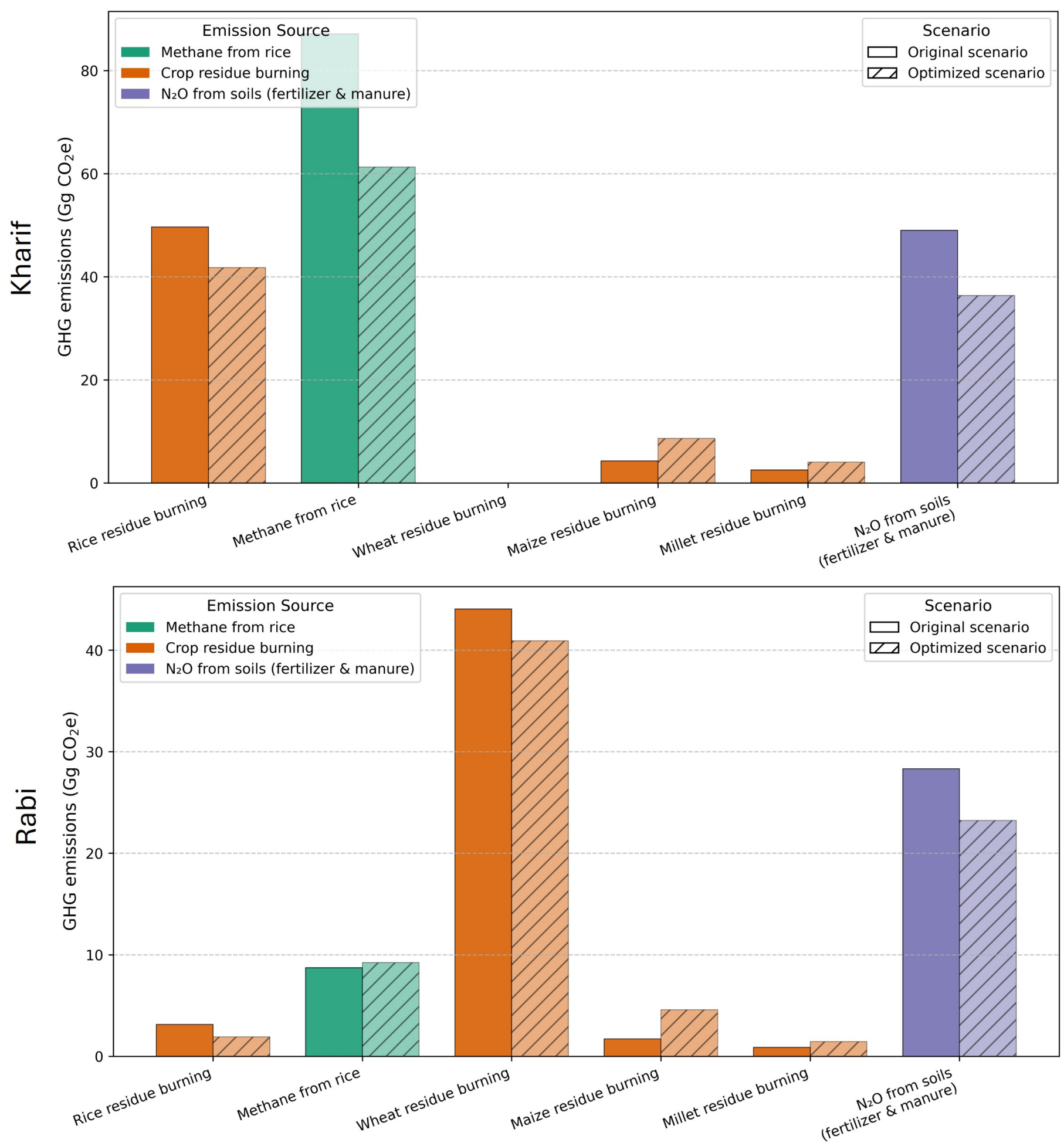

Supplementary Figure 12 – Agricultural greenhouse gas (AGHG) emissions by source for different emission components under nitrogen surplus reduction–based restructuring strategies for the Kharif (top) and Rabi (bottom) seasons. Results are shown for the baseline (original) allocation in 2017 and the optimized scenario, with emissions disaggregated into methane from rice cultivation, crop residue burning, and N_2O emissions from soils (fertilizer and manure).

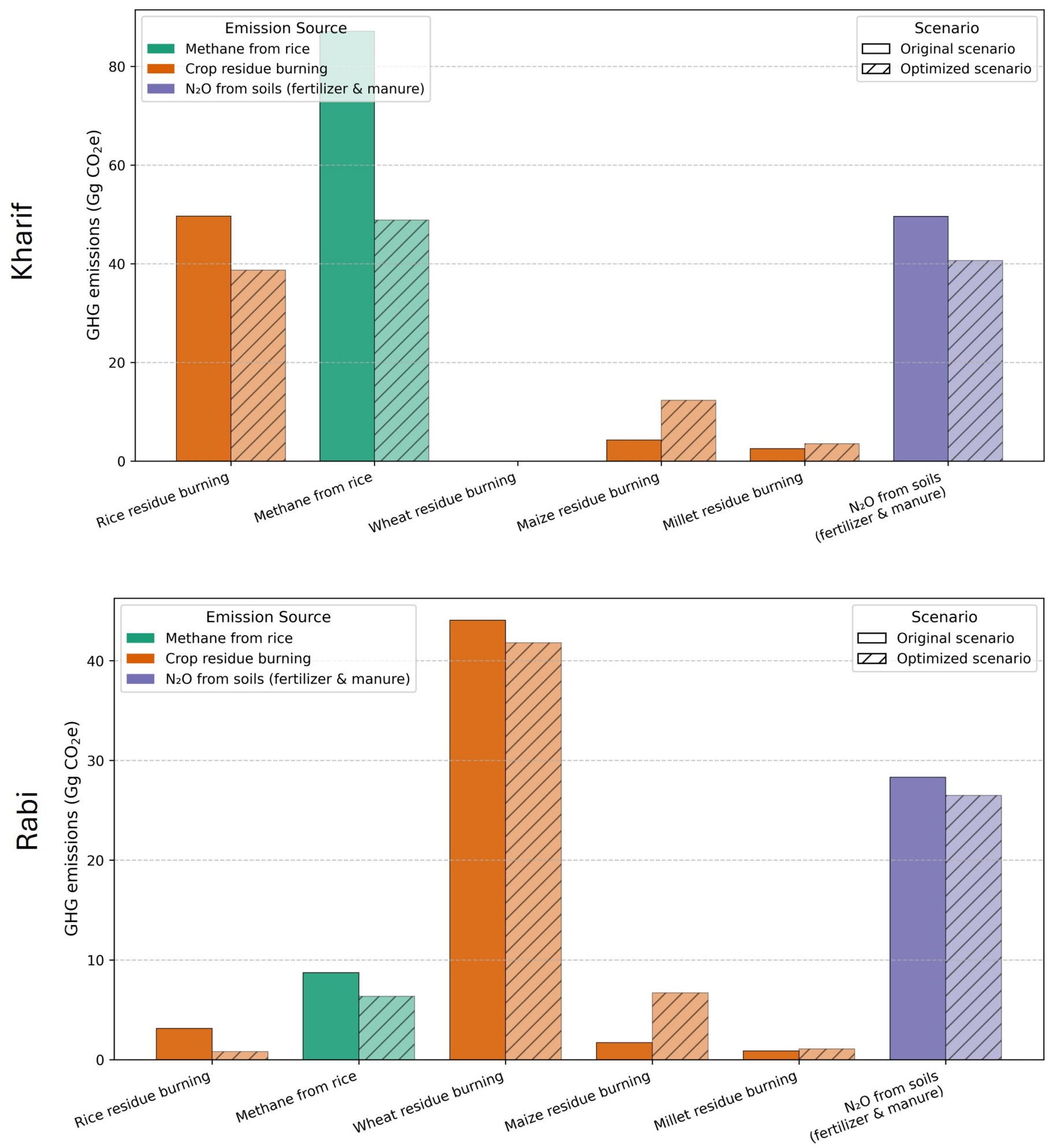

Supplementary Figure 13 – Agricultural greenhouse gas (AGHG) emissions by source for different emission components under water demand reduction–based restructuring strategies for the Kharif (top) and Rabi (bottom) seasons. Results are shown for the baseline (original) allocation in 2017 and the optimized scenario, with emissions disaggregated into methane from rice cultivation, crop residue burning, and N_2O emissions from soils (fertilizer and manure).

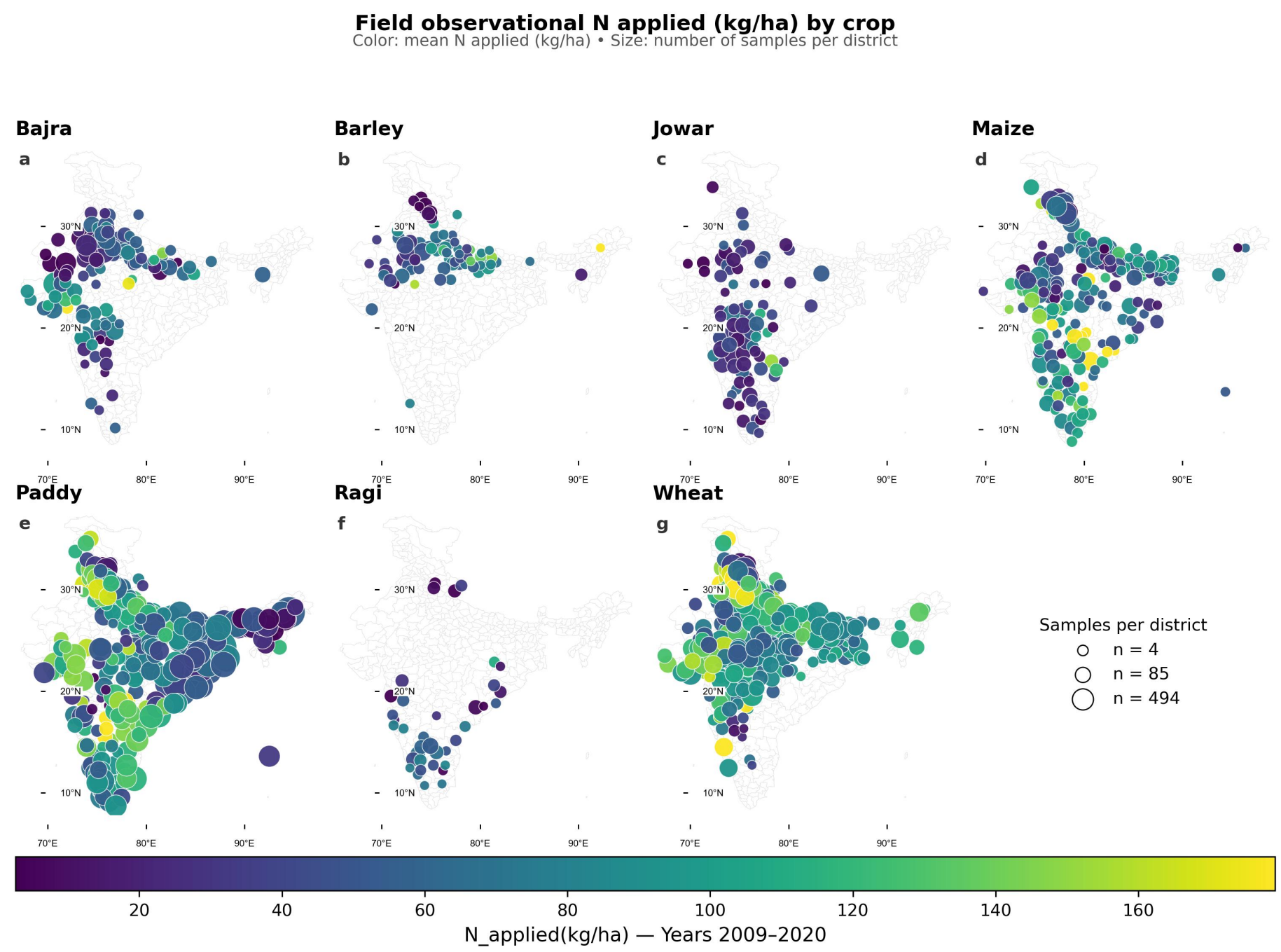

Supplementary Figure 14 – Spatial distribution of field-observed synthetic nitrogen application rates for major cereals in India. District-level mean nitrogen application rates (kg N ha^{-1}; colour scale) are shown for seven major cereal crops — **a** Bajra, **b** Barley, **c** Jowar, **d** Maize, **e** Paddy, **f** Ragi, and **g** Wheat — based on field survey observations collected between 2009 and 2020. Circle sizes indicate the number of observations per district, illustrating heterogeneity in sample density. District boundaries are outlined in grey. District boundary geometries used for map visualization were obtained from the *indian-district-boundaries* GitHub repository, released under the MIT License; Copyright (c) 2020 Guneet Narula [1].

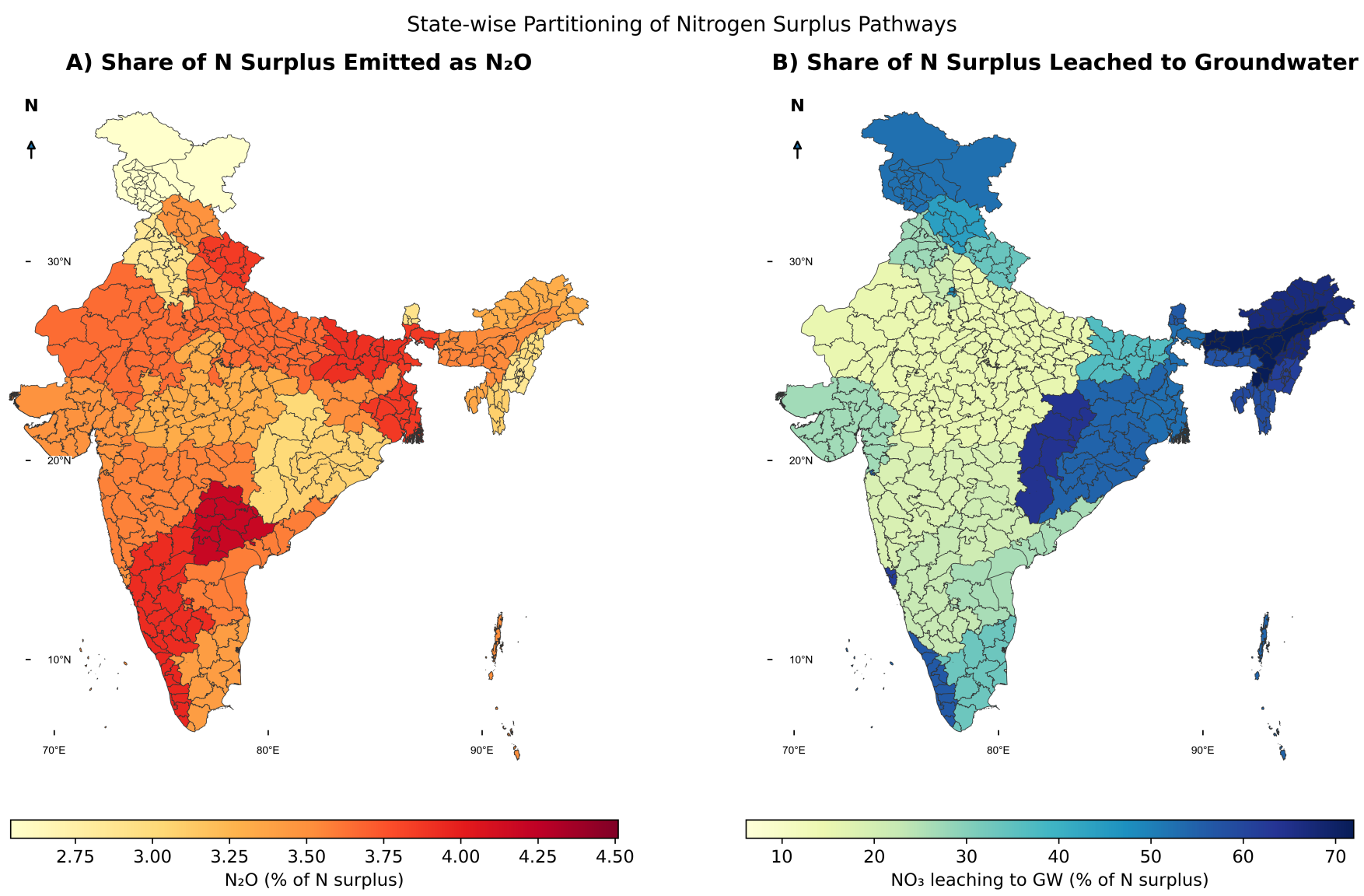

Supplementary Figure 15 – Partitioning of agricultural nitrogen surplus across India. **(a)** Share of N surplus emitted to the atmosphere as nitrous oxide (N_2O, %). **(b)** Share of N surplus leached to groundwater as nitrate (NO_3^-, %). Estimates are from the *IMAGE* Global Nutrient Model (IMAGE–GNM). Values are expressed as the percentage of each state's N surplus; larger values (warmer colours in a, darker blues in b) indicate greater contributions of the respective pathways. District and state boundary geometries used for map visualization were obtained from the *indian-district-boundaries* GitHub repository, released under the MIT License; Copyright (c) 2020 Guneet Narula [1].

Supplementary Table 2 – Nitrogen and phosphorus content of crops used in nutrient-uptake calculations (kg nutrient kg^{-1} grain) [16].

Crop	Nitrogen Content	Phosphorous Content
Rice	0.0129	0.0028
Sorghum (Jowar)	0.0146	0.0045
Bajra	0.0204	0.0042
Maize	0.0124	0.0034
Ragi	0.0204	0.0042

Supplementary Table 3 – Minimum Support Price (MSP) for the focal cereals in 2017.

Crop	MSP (Rs. per Quintal)
Wheat	1735
Paddy (Rice)	1570
Jowar	1712.5
Bajra	1425
Maize	1425
Ragi	1900

Supplementary Table 4 – Calorie values used in the optimization model and selected nutritional indicators of the focal cereals. The calorie constraint is interpreted as a conservative energy-availability floor rather than a full proxy for food security or nutritional security. Protein, calcium, and iron values are reported to contextualize nutritional differences among the focal cereals; these nutrient indicators do not enter the optimization model [6, 17, 18].

**Crop**	**Calories (kcal per 100 g)**	**Protein (g per 100 g)**	**Calcium (mg per 100 g)**	**Iron (mg per 100 g)**
Ragi	321	7.16	364.00	4.62
Bajra	348	10.96	27.35	6.42
Rice	356	7.94	7.49	0.65
Sorghum (Jowar)	334	9.97	27.60	3.95
Wheat	322	10.59	39.36	3.97
Maize	342	8.80	8.91	2.49

Supplementary Table 5 – **Societal and environmental cost categories associated with reactive nitrogen losses.** The table summarizes the main categories of societal and environmental impacts associated with reactive nitrogen losses, including effects on human health, ecosystems, climate, and water quality, following previous nitrogen-assessment literature [19].

Year	Health	Ecosystem	Climate
Nr to water	0.3	3.6	0.0
NH_3 to air	3.6	0.6	0.0
NO_x to air	5.4	0.6	0.0
N_2O to air	0.6	0.0	2.7

Supplementary Table 6 – Fraction of residue burned (FB). State-specific rice and wheat overrides follow Jain et al. [20]; all other cereal-state combinations use the default FB of 0.10 adopted in that inventory for combinations without a reported state-specific override.

State	FB (Rice)	FB (Wheat)
punjab	0.80	0.23
haryana	0.80	0.23
himachal pradesh	0.80	0.23
uttar pradesh	0.25	0.23
karnataka	0.50	0.10
All other states: FB (Rice)=0.10, FB (Wheat)=0.10		

Supplementary Table 7 – Constants and crop parameters used in AGHG calculations.

Parameter	Definition	Value (units)
$GWP_{\mathrm{CH_4}}$	100-yr GWP for CH_4	28 (-)
$GWP_{\mathrm{N_2O}}$	100-yr GWP for N_2O	265 (-)
FAO	Fraction Actually Oxidized	0.93 (-)
$EF_{\mathrm{CH_4}}$	CH_4 EF for residue burning	2.7 (kg t^{-1} residue)
$EF_{\mathrm{N_2O}}$	N_2O EF for residue burning	0.07 (kg t^{-1} residue)
$\mathrm{RCR_{rice}}$	Residue-to-crop ratio (rice)	1.50 (-)
$\mathrm{RCR_{wheat}}$	Residue-to-crop ratio (wheat)	1.70 (-)
$\mathrm{RCR_{maize}}$	Residue-to-crop ratio (maize)	1.50 (-)
$\mathrm{RCR_{millets}}$	Residue-to-crop ratio (millets)	1.50 (-)
$\mathrm{DMF_{rice}}$	Dry matter fraction (rice)	0.86 (-)
$\mathrm{DMF_{wheat}}$	Dry matter fraction (wheat)	0.88 (-)
$\mathrm{DMF_{maize}}$	Dry matter fraction (maize)	0.88 (-)
$\mathrm{DMF_{millets}}$	Dry matter fraction (millets)	0.88 (-)

Supplementary Table 8 – Methane emission factors for rice water regimes (kg CH_4 ha^{-1}).

Water regime	EF (kg CH_4 ha^{-1})
Continuous flooded	159.74
Single aeration	66.20
Multiple aeration	19.30
Upland	0.00
Deepwater	190.00
Drought prone	66.84
Flood prone	189.00

Supplementary Table 9 – Emission factors used for estimating greenhouse gas emissions from open-field burning of crop residues. Values are expressed per tonne of dry matter residue burned. CO_2 from burning of biomass is optional and often excluded from AFOLU GHG inventories following IPCC guidelines.

**Gas**	**Emission Factor (EF)**	**Unit**	**Source**
CH_4	2.70	kg CH_4 t^{-1} residue	[21]
N_2O	0.07	kg N_2O t^{-1} residue	[21]
CO_2 (optional)	1515	kg CO_2 t^{-1} residue	[22]

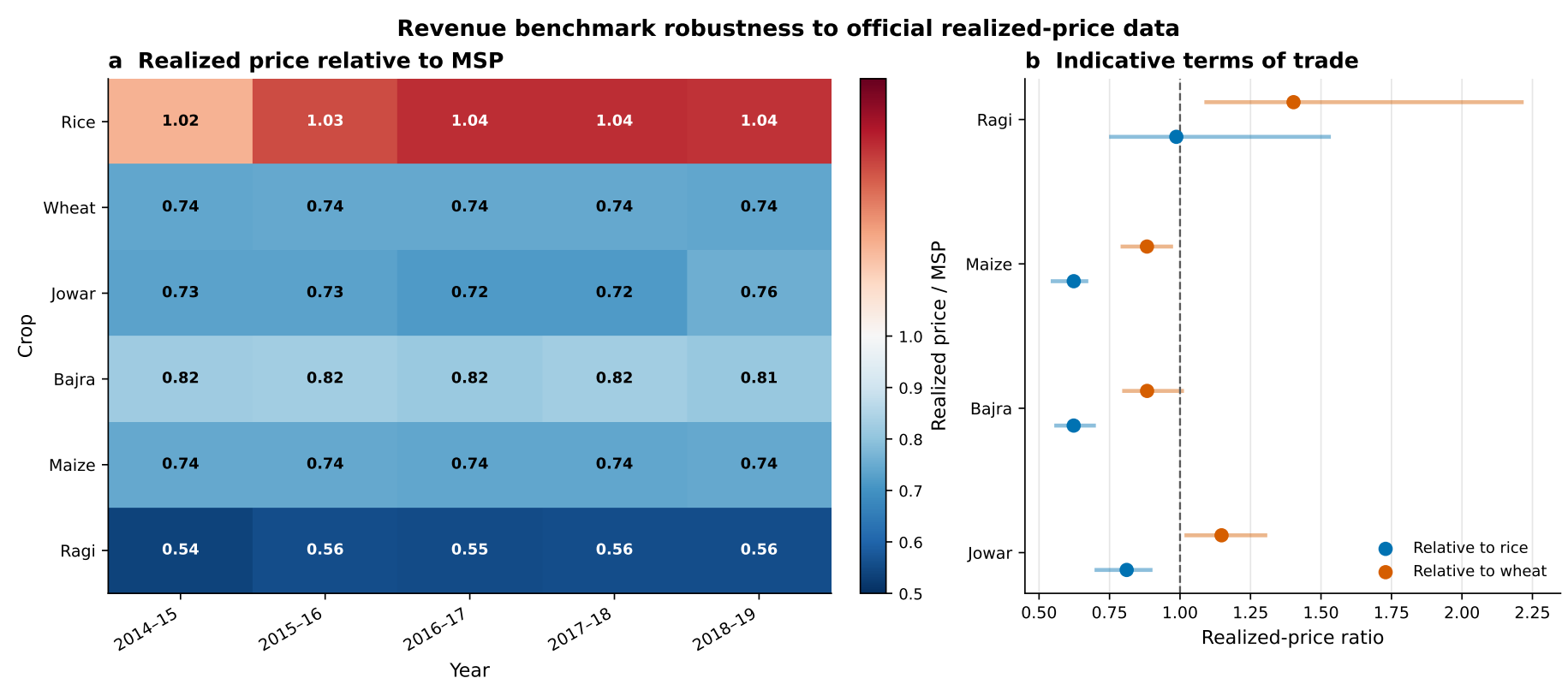

Supplementary Figure 16 – Realized-price diagnostics used to construct the primary 2017–18 revenue benchmark. The primary revenue benchmark replaces the district crop-price term with matched state-year realized prices wherever official state-crop values are available and uses the corresponding all-India crop-level realized price only for the residual unmatched state-crop combinations. **(a)** Median state-level ratio of realized unit price to national MSP for the six focal cereals during 2014–15 to 2018–19. Values above 1 indicate a realized-price benchmark above MSP, whereas values below 1 indicate a realized-price benchmark below MSP. Among the coarse cereals, departures from MSP are heterogeneous rather than uniformly favorable. **(b)** Indicative realized-price terms of trade of alternative cereals relative to rice and wheat, summarized from the all-India realized-price benchmark. Points indicate the mean ratio across 2013–14 to 2017–18 and horizontal line segments show the minimum-to-maximum range across years. Together these diagnostics summarize how the primary realized-price benchmark departs from a uniform MSP benchmark while retaining full modeled cereal coverage through direct state-crop matches and limited all-India fills.

Supplementary Methods 1. Fixed-allocation bootstrap of the main Pareto frontier

The Pareto frontier shown in main Fig. 2a is based on the primary revenue benchmark and the prepared 2017 coefficient field. To examine how local coefficient uncertainty affects that trade-off, we propagated uncertainty through the deterministic optimized allocation at each α, rather than solving a new optimization problem for every perturbation. In each of 500 bootstrap iterations, district crop-specific consumptive water demand and net nutrient application coefficients were perturbed around their 2017 values using sign-symmetric residual draws from the bootstrap pools, while the optimized crop allocation and the state-level calorie and benchmarked-income constraints were held fixed. This produces an uncertainty envelope around the main frontier while keeping the underlying allocation unchanged. The resulting ensemble preserves the same monotonic trade-off and endpoint ordering: the water-based endpoint remains the lowest-water solution, and the nitrogen-based endpoint remains the lowest-nitrogen solution. Supplementary Fig. S19 shows the deterministic frontier, the fixed-allocation bootstrap ensemble, the corresponding alpha-wise envelope, and the endpoint bootstrap summaries.

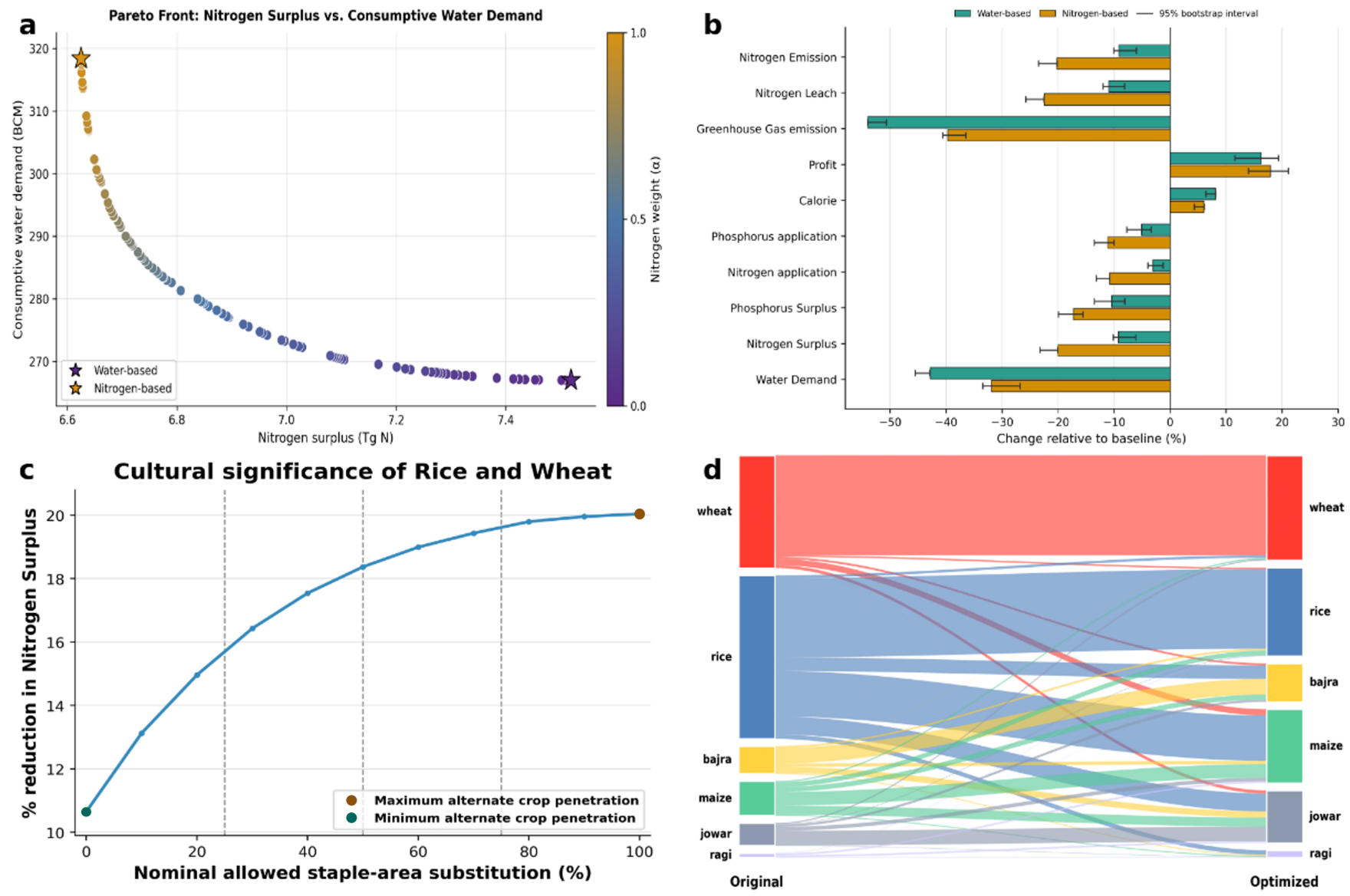

Supplementary Figure 17 – District-MSP benchmark comparison for the Figure 2 result block. These panels are provided for direct comparison with the realized-price-benchmarked main manuscript. Under the district-MSP benchmark, the qualitative ranking between water-focused and nitrogen-focused restructuring is preserved, but the absolute endpoint gains are larger than in the primary realized-price benchmark. **(a)** Pareto-front analysis detailing the trade-offs between nitrogen surplus and total water demand for the combined rabi and kharif system. Each data point represents an optimal solution obtained by balancing different levels of water- and nitrogen-minimization objectives under the district-MSP benchmark. **(b)** Percentage change in socio-environmental objectives under water-based and nitrogen-based restructuring across the combined rabi and kharif seasons. The objectives include AGHG emissions, nitrogen and phosphorus application and surplus, nitrogen leaching and emissions, calorie production, and benchmarked cereal net return. Colored bars show the national aggregate percentage change. Black whiskers show 95% bootstrap intervals from n=500 bootstrap resamples, propagated district-input uncertainty for each displayed metric under each endpoint strategy. **(c)** Sensitivity of combined nitrogen-surplus reduction to progressively relaxed state-level rice and wheat retention constraints for the combined rabi and kharif system. **(d)** Total cropland area transformation before and after nitrogen-surplus minimization under the district-MSP benchmark.

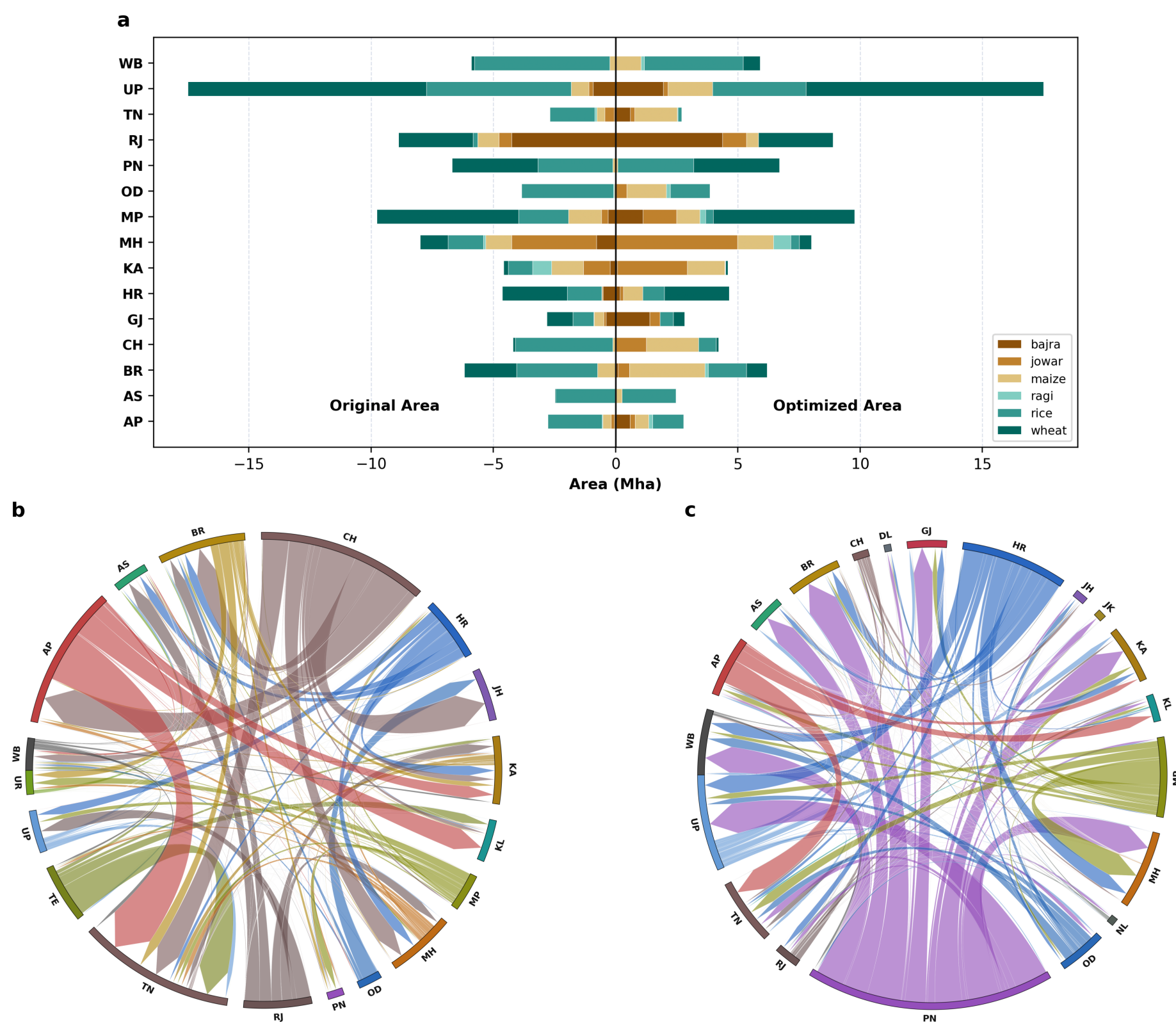

Supplementary Figure 18 – District-MSP benchmark comparison for the Figure 3 result block. These panels provide the corresponding trade and area composites under the district-MSP benchmark used for Supplementary Fig. S17. Relative to the primary realized-price benchmark, the district-MSP comparison yields stronger expansion of alternative-cereal trade while preserving the qualitative restructuring pattern. **(a)** Comparison of original and optimized cropland areas at the state level under the nitrogen-focused district-MSP solution. **(b)** Restructured interstate trade of combined alternative cereals, including maize, ragi, bajra, and jowar, expressed in kilocalories after optimization, with newly introduced links where staple-calorie deficits emerge on state pairs without baseline alternative-cereal trade. **(c)** Restructured interstate trade of combined rice and wheat, expressed in kilocalories after optimization under the same district-MSP benchmark. In panels **(b)** and **(c)**, arrows indicate flow from exporting to importing states, link colour denotes the exporting state, and link width is proportional to optimized trade volume.

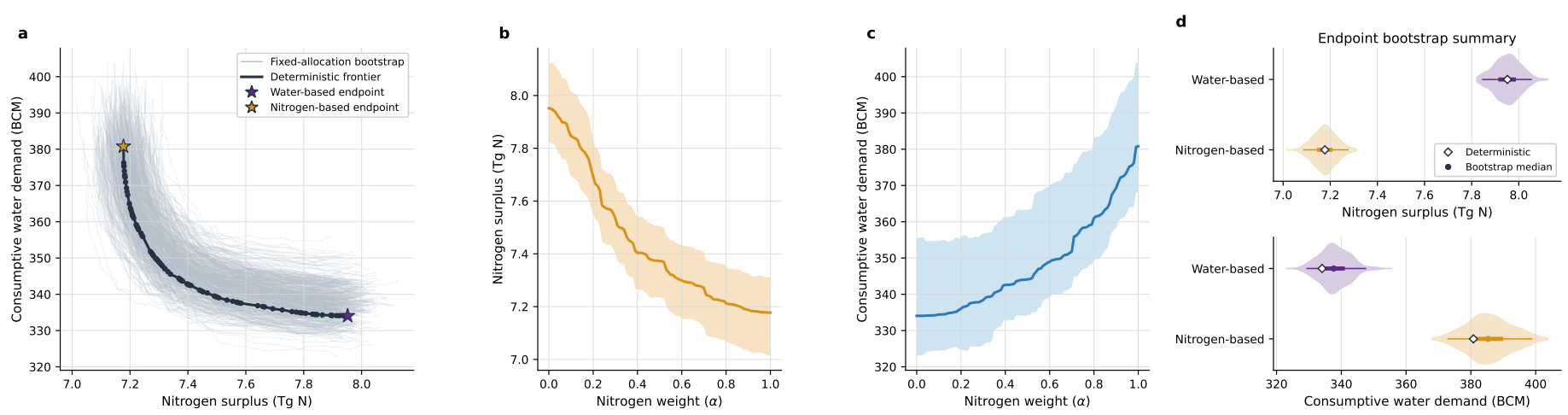

Supplementary Figure 19 – Bootstrap robustness of the main Pareto frontier shown in Fig. 2a. (a) The deterministic Pareto frontier under the primary revenue benchmark is overlaid on 500 fixed-allocation bootstrap evaluations obtained by perturbing district crop-specific consumptive water-demand and net-nitrogen application coefficients around their 2017 values using sign-symmetric residual draws. The deterministic optimized allocation at each α is held fixed during these evaluations, so the ensemble captures coefficient-propagation uncertainty around the reported frontier rather than a family of newly optimized frontiers. Star symbols denote the water-based ($\alpha = 0$) and nitrogen-based ($\alpha = 1$) endpoints. **(b)** Deterministic nitrogen surplus along the frontier (solid line) and the corresponding minimum-to-maximum span across the deterministic frontier and fixed-allocation bootstrap ensemble at each α (shaded band). **(c)** Deterministic consumptive water demand along the frontier (solid line) and the corresponding minimum-to-maximum span defined in the same way. **(d)** Bootstrap endpoint summaries for nitrogen surplus and consumptive water demand at the water-based and nitrogen-based endpoints. Violin shapes show the fixed-allocation bootstrap distributions, thick horizontal segments show the interquartile range, thin horizontal segments show the 95% interval (n=500), circles mark the bootstrap median, and diamonds mark the deterministic endpoint values. Across the frontier, coefficient perturbations widen the local trade-off but do not alter the monotonic frontier shape or the qualitative ordering of the water-based and nitrogen-based endpoints.

Supplementary Discussion 1. Seasonal interpretation of the annual reallocation summary

Main Fig. 2d combines the nitrogen-focused kharif and rabi reallocations under the primary revenue benchmark. Supplementary Fig. S20 shows the same result season by season and highlights district-seasons in which rice declines while wheat increases. No baseline kharif wheat area is present in the primary dataset, so the apparent rice-to-wheat transition in the annual panel does not reflect a within-season kharif substitution. Instead, it appears only after the kharif and rabi solutions are aggregated into a single annual summary. The same-season rice-loss/wheat-gain pattern is limited to rabi districts that already cultivate rabi rice in the baseline. By contrast, the dominant seasonal reallocations remain rice to maize, jowar, bajra, and ragi in kharif, and wheat to maize, jowar, and bajra in rabi.

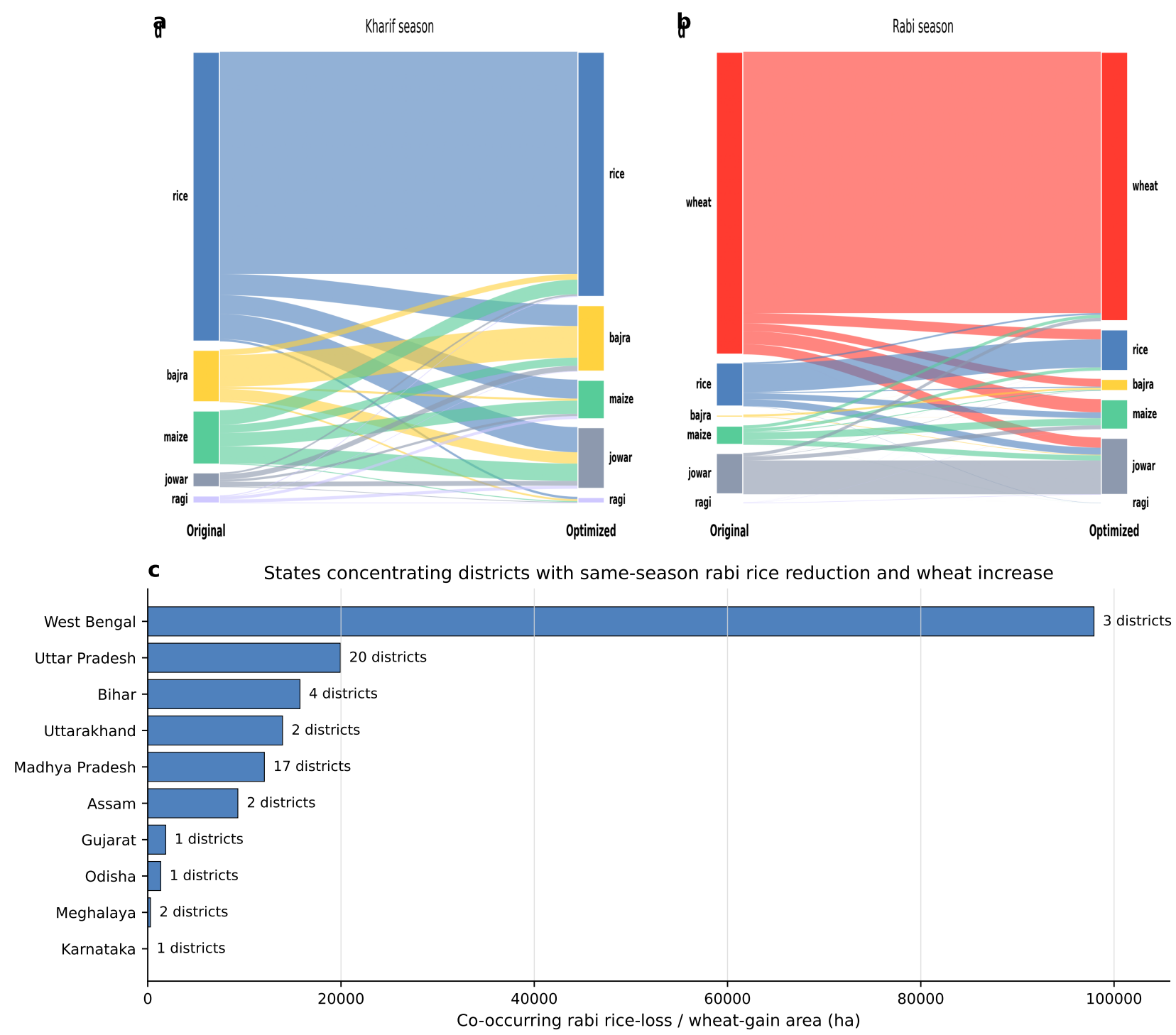

Supplementary Figure 20 – Seasonal structure of the nitrogen-focused area reallocation shown in Fig. 2d. **(a)** Kharif-season crop-reallocation flow summary. **(b)** Rabi-season crop-reallocation flow summary. Panels **(a)** and **(b)** use the same within-district-season allocation rule as the combined annual panel. **(c)** States containing district-seasons in which rabi rice decreases while rabi wheat increases. Because the primary dataset contains no baseline kharif wheat area, the apparent annual rice-to-wheat crossover in the combined panel reflects aggregation across seasons rather than a kharif within-season substitution.

Supplementary Methods 2. Production-cost benchmark interpretation

The production-cost term is derived from the published state-level crop-specific *cost of production* series reported in *Agricultural Statistics at a Glance 2017*. This series is merged by crop and state and then applied uniformly across districts within each state. In the source table, cost of production is reported in Rupee quintal^{-1} on the DES C2 basis, while cost of cultivation is reported separately on A2+FL and C2 bases in Rupee hectare^{-1}. The production-cost term used in the optimization should therefore be interpreted as a state-level C2 cost-of-production benchmark rather than a district-level reconstruction of farm costs. Because the DES C2 concept includes imputed owned-resource components, the benchmark is broader than a cash-cost measure such as Cost A1 or A2+FL.

For clarity, Supplementary Table 10 summarizes the standard DES cost concepts most relevant to this interpretation. These concepts distinguish paid-out expenses from the imputed costs of owned land, owned capital, and family labour. In that framework, the implemented benchmark should not be read as a narrow cash-cost measure such as Cost A1 or A2+FL. It is a C2-based state-level production-cost benchmark applied uniformly to districts within each state-crop pair. The formal definitions used in Indian agricultural cost accounting are given in the DES manual [23]. Supplementary Table 11 summarizes how this cost benchmark and the two revenue benchmarks enter the optimization.

Supplementary Table 10 – Standard DES agricultural cost concepts used to interpret the production-cost benchmark in the optimization model.

**Concept**	**Interpretation**
Cost A1	All paid-out expenses in cash and kind, including seed, fertilizer, manure, pesticides, hired labour, machinery charges, irrigation charges, land revenue, depreciation, and interest on working capital.
Cost A2	Cost A1 plus rent paid for leased-in land.
A2+FL	Cost A2 plus imputed value of unpaid family labour.
Cost B1	Cost A1 plus interest on owned fixed capital, excluding land.
Cost B2	Cost B1 plus rental value of owned land and rent paid for leased-in land.
Cost C1	Cost B1 plus imputed value of family labour.
Cost C2	Cost B2 plus imputed value of family labour.
Cost C3	Cost C2 plus a managerial margin; not used in the present benchmark.

Supplementary Table 11 – Operational treatment of income-benchmark inputs in the optimization model. These definitions clarify how the production-cost benchmark sits within the standard DES framework and how revenue benchmarks are implemented in the optimization.

Input	Operational treatment
Production-cost benchmark	Source series: *Agricultural Statistics at a Glance 2017*. Native resolution: crop × state. Native unit: Rupee quintal^{-1}, reported on the DES C2 cost-of-production basis. It is distinct from the per-hectare cultivation-cost entries reported elsewhere in the source tables. In the optimization, this series is merged by crop and state to district observations, so the same state-level C2 value is applied to all districts within a state. Total district production cost is computed as 10× production in tonnes × the benchmark.
Primary revenue benchmark	Source series: MoSPI state-year current-price value of output + DES APY state-year production. Native resolution: crop × state × year for directly matched realized prices; crop × India for the all-India realized-price fill used only where a usable direct state-crop value was unavailable. In the optimization, matched state-year realized prices are applied uniformly to districts within a state-crop pair, and the small residual unmatched area uses the corresponding all-India crop-level realized price. Total district sales revenue is computed as 10× production in tonnes × the benchmark price.
District-MSP comparison benchmark	Source series: Government of India MSP statements. Native resolution: crop × India. Native unit: Rupee quintal^{-1}. In the comparison benchmark, MSP is applied uniformly across districts for a given crop. Total district sales revenue is computed as 10× production in tonnes × MSP.

Supplementary Methods 3. BNF input term in the nitrogen balance

To clarify the nitrogen-balance inputs used in the cereals-only optimization, Supplementary Table 12 reports the BNF term carried into the 2017 baseline model run. In the implemented workflow, the annual FAOSTAT cropland nutrient-budget BNF rate was merged by year and therefore applied uniformly across the six focal cereals. This term should therefore be interpreted as an exogenous cropland nutrient-budget input in the nitrogen balance, rather than as crop-specific symbiotic fixation by cereal plants or as district-level biofertilizer use.

Supplementary Table 12 – Implemented biological nitrogen fixation (BNF) input term used in the 2017 baseline optimization. The value represents the annual FAOSTAT cropland nutrient-budget estimate merged by year and applied as a uniform background N input across the six-cereal optimization system. It should not be interpreted as crop-specific nodulating fixation by cereals.

Input term	Value
Uniform cropland BNF input	31.8908 kg N ha^{-1} yr^{-1}

References

1. Narula, G. *indian-district-boundaries* GitHub repository. Licensed under the MIT License. Copyright (c) 2020 Guneet Narula. 2020. https://github.com/guneetnarula/indian-district-boundaries.
2. Government of India. *Crops APY Report Web* https://data.desagri.gov.in/website/crops-apy-report-web. Accessed: 2024-07-16. 2024.
3. Government of India. *Agricultural Statistics at a Glance 2017* https://desagri.gov.in/wp-content/uploads/2021/04/Agricultural-Statistics-at-a-Glance-2017.pdf. Accessed: 2024-07-16. 2017.
4. Government of India. *MSP Statements* https://farmer.gov.in/mspstatements.aspx. Accessed: 2024-07-16. 2024.
5. Allowances, R. D. Nutrient requirements and recommended dietary allowances for Indians. *ICMR-National Institute of Nutrition: Hyderabad, India* (2009).
6. Longvah, T., Ananthan, R., Bhaskarachary, K. & Venkaiah, K. *Indian Food Composition Tables* https://www.nin.res.in/ebooks/IFCT2017.pdf (National Institute of Nutrition, Indian Council of Medical Research, Hyderabad, India, 2017).
7. Government of India. *DESAGRI. Plot-wise Summary Data* https://desagri.gov.in/document-report-category/plot-wise-summary-data/. Accessed: 2024-07-16. 2024.
8. *Input Datasets for Model Intercomparison Projects: N Deposition* https://esgf-node.llnl.gov/search/input4mips/. Accessed: 2024-04-04. 2024.
9. Food and Agriculture Organization of the United Nations. *FAOSTAT Statistical Database* https://www.fao.org/faostat/en/. Accessed: 2026-04-21. 2026.
10. Dhingra, S., Singh, D. & Mehta, R. *Methodology Note: National & State Level Greenhouse Gas Estimates, 2005–2015 — Agriculture, Forestry and Other Land Use Sector (AFOLU)* Vasudha Foundation and GHG Platform India, New Delhi. Methodology note, Version 3.0, Phase III. 2019. https://www.ghgplatform-india.org/wp-content/uploads/2023/03/AFOLU-Methodology-Note-GHG-Phase-III.pdf.
11. Kampman, D. A., Hoekstra, A. Y. & Krol, M. S. *The water footprint of India* Value of Water Research Report Series (Unesco-IHE Institute for Water Education, Delft, The Netherlands, 2008), 1–152. https://research.utwente.nl/en/publications/the-water-footprint-of-india/.
12. Ministry of Commerce and Industry. *Directorate General of Commercial Intelligence and Statistics* http://www.dgciskol.gov.in/. [Online; accessed 30-September-2022]. 2021.

13. Beusen, A., Van Beek, L., Bouwman, A., Mogollón, J. & Middelburg, J. Coupling global models for hydrology and nutrient loading to simulate nitrogen and phosphorus retention in surface water–description of IMAGE–GNM and analysis of performance. *Geoscientific model development* **8,** 4045–4067 (2015).

14. Ministry of Statistics and Programme Implementation, Government of India. *Statistical Report on Value of Output from Agriculture and Allied Sectors* `https://www.mospi.gov.in/publications-reports/innerpage/431`. Accessed: 2026-04-18. 2025.

15. Unified Portal for Agricultural Statistics, Government of India. *UPAg: Unified Portal for Agricultural Statistics* `https://upag.gov.in/`. Accessed: 2026-04-18. 2026.

16. Ludemann, C. I. *et al.* A global FAOSTAT reference database of cropland nutrient budgets and nutrient use efficiency (1961–2020): nitrogen, phosphorus and potassium. *Earth System Science Data* **16,** 525–541 (2024).

17. DeFries, R. *et al.* Impact of historical changes in coarse cereals consumption in India on micronutrient intake and anemia prevalence. *Food and nutrition bulletin* **39,** 377–392 (2018).

18. Damerau, K. *et al.* India has natural resource capacity to achieve nutrition security, reduce health risks and improve environmental sustainability. *Nature Food* **1,** 631–639 (2020).

19. Sutton, M. A. *et al. The Indian nitrogen challenge in a global perspective* In *The Indian Nitrogen Assessment: Sources of Reactive Nitrogen, Environmental and Climate Effects, Management Options, and Policies*, pp. 9–28. Elsevier. 2017.

20. Jain, N., Bhatia, A. & Pathak, H. Emission of Air Pollutants from Crop Residue Burning in India. *Aerosol and Air Quality Research* **14,** 422–430 (2014).

21. Andreae, M. O. & Merlet, P. Emission of trace gases and aerosols from biomass burning. *Global Biogeochemical Cycles* **15,** 955–966 (2001).

22. Intergovernmental Panel on Climate Change. *2006 IPCC Guidelines for National Greenhouse Gas Inventories* (Institute for Global Environmental Strategies, Hayama, Japan, 2006).

23. Directorate of Economics and Statistics, Ministry of Agriculture, Government of India. *Manual on Cost of Cultivation Surveys* `https://desagri.gov.in/wp-content/uploads/2021/06/manual_cost_cultivation_surveys_23july08_0.pdf`. Accessed: 2026-04-16. 2008.
