# Supplementary material for: Quantifying environmental co-benefits of nitrogen-based crop restructuring and its implications on India’s interstate trade network
Source: Nat Commun. 2026 Aug 4;17:7778. doi: 10.1038/s41467-026-75905-w (PMC13438110; doi:10.1038/s41467-026-75905-w)
Supplement: Supplementary file 3 [file 41467_2026_75905_MOESM3_ESM.pdf]

Peer Review File

Quantifying Environmental Co-Benefits of Nitrogen-Based Crop Restructuring and Its Implications on India's Interstate Trade Network

Corresponding Author: Dr Udit Bhatia

This file contains all reviewer reports in order by version, followed by all author rebuttals in order by version.

Version 0:

Reviewer comments:

Reviewer #2

(Remarks to the Author)
Reviewer Report
Title of Manuscript: Quantifying Environmental Co-Benefits of Nitrogen-Based Crop Restructuring and Its Implication on Trade Network System
Noteworthy Results:
The manuscript presents several noteworthy results. The nitrogen-focused crop restructuring strategy significantly reduces nitrogen surplus by 16–24% and water use by 20–40%. Additionally, it improves farmer income, calorie production, and mitigates greenhouse gas emissions by 28% (113 Mt CO2 eq). The strategy is shown to be five times more efficient in delivering cross-benefits compared to water-focused restructuring strategies, resulting in a decrease in socio-environmental costs associated with nitrogen pollution by approximately $1.97 billion USD. Furthermore, the restructuring leads to a 4.3 times increase in the volume of alternative crop trade in the interstate trade network.
Significance to the Field:
The work is highly significant to the field of agricultural sustainability and related fields such as environmental science and economics. It provides new insights into the benefits of nitrogen management in crop restructuring, which could influence future agricultural policies and practices. Compared to the established literature, this work offers a more comprehensive analysis of the environmental and economic co-benefits of nitrogen-focused strategies, highlighting its superiority over water-focused approaches.
Originality:
The manuscript appears to be original in its approach and findings. It uniquely combines nutrient management with trade network implications, which is not extensively covered in existing literature. If there are similar works, relevant references should be provided to contextualize the novelty of this study.
Support for Conclusions and Claims:
The conclusions and claims made in the manuscript are well-supported by the data presented. The evidence provided is robust, though additional data on long-term impacts and regional variations could further strengthen the claims.
Flaws in Data Analysis, Interpretation, and Conclusions:
Described method for calculating the net input of nitrogen (N) and phosphorus (P) application rates is comprehensive, there are potential shortfalls to consider. The accuracy of the results heavily depends on the reliability and consistency of the data sources, and any inaccuracies or inconsistencies could lead to erroneous calculations. Additionally, the method may not fully account for regional variations in agricultural practices, soil types, and climatic conditions, which can significantly influence N and P application rates and their effectiveness. Temporal changes in agricultural practices and environmental conditions might not be adequately captured, potentially leading to outdated or less relevant results. Estimating contributions from biological fixation and atmospheric deposition can be challenging and may introduce uncertainties. Furthermore, farmers' survey data might have limitations such as recall bias, incomplete responses, or sampling errors, affecting the overall accuracy of the input data. Addressing these potential shortfalls would involve ensuring high-quality, up-to-date data, considering regional and temporal variations, and carefully estimating contributions from all sources.
Exchange rate and deflation methodology (from the base year) has not mentioned
Manuscript has not organised properly (research gap, limitations of the study are missingand after introduction directly it went to results)
Other than the above listed there are no flaws in the data analysis, interpretation, and conclusions that prohibit publication.

However, some areas could benefit from further clarification. These revisions would enhance the manuscript's clarity and reliability.
Methodology:
The methodology used in the study is sound and meets the expected standards in the field. The authors have employed appropriate techniques to analyze the environmental and economic impacts of nitrogen-based crop restructuring. However, a more detailed description of the data sources and analytical methods would be beneficial for reproducibility.
Reproducibility:
The manuscript provides sufficient detail in the methods section to allow for reproducibility. The descriptions of the nitrogen and water use calculations, as well as the trade network analysis, are clear and comprehensive. Any additional details that could further aid reproducibility include specific data sources and any assumptions made during the analysis.
Overall Recommendation:
The manuscript is a valuable contribution to the field and, with minor revisions, should be suitable for publication. The authors are encouraged to address the highlighted issues to improve the manuscript's overall quality.

Reviewer #3

(Remarks to the Author)
The manuscript by Goyal et al. quantifies the environmental co-benefits of nitrogen-based crop restructuring and its implication on trade network for India. The manuscript is well-written and self-explanatory. However, although the title does not mention the geographical area studied and the abstract remotely mentioned global extrapolation, albeit by default, it is essentially a country-specific analysis. In that backdrop, the global context is missing as the data is not extrapolated to global budget.

Considering the subject matter of this manuscript, the element of novelty is missing as several such studies have been conducted worldwide with focussed global analysis including India scenario as well. Due to specific analysis based on data originating from India, as has been done in this manuscript, the global appeal of the manuscript gets restricted. This is evident from the references cited in the manuscript and literature available. In addition, nitrogen impacts need to be considered along with water relations and there is an element of synergy among the two factors influencing crop productivity. Although, the authors did refer to such relationship, they did not make attempt for such analysis and exclusively focussed on N relations only.

The analysis based on a linear algorithm, originally envisaged several objectives including crop restructuring, environmental trade-off and inter-state trade, and should have involved multi-nodal analytical algorithms. N-pollution and global health analysis has also been a subject of much research in China, Germany, Netherlands and the Climate analysis group of FAO, with several interdisciplinary models having been developed (See the publications of Dr. Baojing Gu). Thus, going by the objectives stated by the authors, this analysis gap is evident.

I have a few specific inquiries:
Abstract: Line 14: Delete ‘into their strategies’.
Keywords: Add ‘system efficiency’.
Introduction:
Line 1 : Replace ‘in’ with ‘for’
Line 9 : Change to “emphasis on high-yielding crop cultivars”…………..
Line 17 : Suggest replace ‘rice and wheat’ with ‘cereals’
Line 18-20: Change to “mainly N-fertilizers leading……………… and over use of water resources……”
Line 23 : Change to ‘all the while’
Line 50 : Surplus of Nitrogen: not all nitrogen has environmental impact, it is only reactive or combined-N that is important. Introduce the concept of reactive-N (Nr) here.
Line 53 : Change to ‘……environmental and biological…..’
Line 55 : Change to Nr in connection with the change suggested at line 50
Line 59 : Ref, 39: Quite an volume of literature is available on this topic. Authors may refer to Lassaletta et al.
Line 73 : ‘minimization’ : do you mean ‘reduction’?
Line 75 : AGHG probably authors like to mention ‘agricultural greenhouse gas’’. This abbreviation is missing from the list of ‘Abbreviations’
Line 99 : ‘eccentric’ , do you mean ‘centric’ or ‘dependent’?
Results:
Line 127-128: This statement may not be true. Maize, especially the double cross hybrids are quite high yielder so also the modern varieties of finger millet. Maize in fact has very high yield potential and as far as India is concerned it has a CAGR of 1.34 per cent for the decade 2021-2031 and consumption CAGR is 1.82 per cent during the same period. However, 60 per cent of the produce is used as animal feed and only 13% is used as food.
Lines 152-153: This figure of water reduction by only crop replacement requires further clarification. Used of moder rice growing using water reduction technologies, especially for the latest improved varieties of ‘Basmati’ rice has resulted into reduction of water use while maintaining the yield or even having higher yield resulting into high water use efficiency. This has resulted into India emerging as the largest exporter of rice.
Fig. 2: Virtual export of water and Nitrogen through agricultural export (both intra and inter-country scenarios) has been studied and some information is available. These should be brought in for ‘Discussion’ and analyzing further reduction through crop replacement during suggestion for ‘Trade network restructuring’.
Line 186: N-centric?

Line 235 : Cultural consideration should also consider socio-economic perspective.
Line 239 : Replace 'quick' with 'easy'
Discussion:
Uncertainty analysis and its mitigation should be analyzed quantitatively and integrated in the model. Indeed, it is mentioned as 'constraints (line 554) but requires clarification.
Methods:
Line 477 : Nsurplus calculation must include N-leakage from the system. Mention is made but introduction of the factor in the equation is required.
Line 516 : 'Agricultural Statistics at a Glance'
Line 526 : For the international audience all values requires to be converted into USD.
Line 580 : The 'limitations' should reflect in the model.
Line 687 : It is better to use Indian data reflected in the BUR-3 report published by the Ministry of Environment (MoEFCCC), Govt. of India. Now India already has Tier III dataset which could refine the estimate and incorporated in the model.
Line 700 : Nitrogen loss cannot be a default value for a large country (subcontinent size) like India.
General: How the data was retrofitted in the map of India, which model?

Reviewer #4

(Remarks to the Author)
The paper is methodologically sound and substantiate the case as to why nutrient-centric agricultural restructuring could make sense in India. The use of multi-objective optimization model to evaluate trade-offs and co-benefits of minimizing nitrogen surplus and water use was able to explain the implications for sustainability, food security, and interstate trade. It's also notable that the authors considered the socio-economic and cultural context in their methodology.

Here are my comments on the paper:
1. The authors used IMAGE-GNM emission and leaching factors. However, these are global-scale model outputs and may not accurately reflect India's heterogeneity in soils, rainfall, and management. I suggest to add a brief discussion on this limitation and suggest more localized empirical data or models that could enhance future versions.
2. The model included valuable constraint on minimum rice/wheat cultivation due to cultural importance, but adoption barriers like market access, taste preferences, processing infrastructure, and farmer skill adaptation were not fully addressed. Maybe add a brief discussion regarding these practical adoption constraints and how they could be incorporated in future modeling
3. Post-optimality sensitivity analysis was undertaken and real-world constraints (i.e. minimum caloric output, no expansion beyond historical crop areas, and farmer profit stability) ground the model in realistic agronomic and economic conditions. However, empirical/external validation was lacking. While this is understandable given the scale and ambition of the work, the policy relevance of the study would be significantly strengthened by empirical comparisons or even qualitative alignment with existing field programs and observed trends.
4. Figure labels and legends are too small and hard to read. Increase size of plots and text font to make them more legible. Consider breaking up multiple plots (i.e. Fig. 2) if appropriate. "Pareto" was misspelled in Fig. S2. The color of the bar (profit) for Fig. S3b confused me. My sense was red color denote an increase in percentage. I also suggest dropping the "%" on the labels for the tradeoffs on the y axis on S3 since it was already stated on the x axis label.

Overall, it is an excellent paper offering quantitative evidence for how nitrogen surplus management can lead to broad environmental and economic co-benefits, especially in the Indian context. I recommend to accept this paper with minor revisions to address the above points.

Version 1:

Reviewer comments:

Reviewer #3

(Remarks to the Author)
Authors have positively responded to the comments of the Revieweres and made required modifications/changes in the text and also in the supplementary. The article now reads well.

(Remarks on code availability)

Reviewer #4

(Remarks to the Author)
I have carefully reviewed the authors' revised manuscript and their detailed responses to my comments. I am satisfied that all of my concerns have been thoroughly addressed, and the revisions have significantly improved the clarity, rigor, and policy relevance of the paper. Overall, I find the manuscript suitable for publication in its current form.

(Remarks on code availability)

Reviewer #6

(Remarks to the Author)
Comments on “Quantifying environmental co-benefits of nitrogen-based crop restructuring and its implications on India’s interstate trade network”.

• Using multi-objective optimization model, the paper has developed a framework and demonstrated the superiority of nitrogen-focussed crop restructuring strategy in lowering nitrogen surplus along with producing water-saving over water-focussed restructuring, while maintaining the calorie production and farmers income. The model also uniquely integrates crop restructuring with inter-state trade flow for ensuring compensation of potential calorie deficits from crop restructuring through increased trade volume of alternative crops. Overall, the paper provides useful insights on leveraging the synergy between nutrient and water management and aligns with the national and global priorities on sustainable agriculture.

• Broadly, the model restructures the cropping pattern towards the cereal crops (Jowar, Bajra, Ragi, Maize) that minimizes environmental externalities without significantly impacting the food security and farmers income. While the theoretical framework is robust, the empirical assessment requires careful scrutiny of the certain parameters used and inferences drawn.

o In order to reflect realistic agricultural scenario, crop area optimization has been imposed with the constraints of maintaining calorie production and farmers income. The farmers income has been estimated at district-level by deducting production cost with sale revenue. The authors have used national-level minimum support price (MSP) for estimating sale revenue of crops at district level. Valuating production with administratively fixed MSP does not represent realistic agricultural market because MSP is primarily designed as a safety net mechanism rather than reflecting a true value of produce at competitive market. Further, it is characterized with limited access (only 15% of paddy farmers and 9.6% of wheat farmers availed MSP in 2018-19) with significant variation across crops and geographical regions. MSP-based procurements are biased towards crops like paddy and wheat, while the alternate crops in the study (maize, jowar, bajra and ragi) are largely excluded from MSP-based procurement. Using MSP for estimating income, therefore, masks inter-crop disparity in actual price realization and produces inconsistent revenue terms of trade between crops- a key factor driving farmers’ decision on crop choices. Consequently, the optimization results may not reflect real market scenario. A simple alternative to MSP could be ‘unit-prices’ for each crop – derived by dividing value of crop output with production. Beside representing actual average prices realized by farmers for crop produce, unit-prices can be estimated at state level and thus provide more variability in data than using single national-level MSP. The official data on value of output and production of crops is also readily available at https://www.mospi.gov.in/publications-reports/innerpage/431 and https://upag.gov.in/, respectively.

o Any inference on the crop reallocations obtained using MSP-based estimates on farmers income would be conditional on realization of MSP for each crop which is not a typical characteristic of Indian agricultural market. This refutes authors’ claim of incorporating realistic agricultural scenario in the model. Underline conditionalities of the results shall at least be explicitly discussed in the paper.

o Estimates on production cost used in the analysis need to be elaborated using standard terminology. It is not clear whether cost includes only paid-out expenses or imputed value of owned resources (family labour, own land, etc) as well. It is recommended to use standard cost concepts (eg Cost A1/Cost B1/Cost C1) for ensuring clarity and uniform comprehension. Standard cost concepts used in Indian context can be obtained at https://desagri.gov.in/wp-content/uploads/2021/06/manual_cost_cultivation_surveys_23july08_0.pdf

• Usually alternate crops like jowar, bajra, ragi and maize remain less remunerative than rice and wheat, disincentivizing farmers to diversify away from the latter crops. As the resultant crop reallocations are in favour of alternate crops (jowar, bajra, ragi and maize), it would be plausible to provide revenue terms of trade between competing crops as Supplementary information.

• It would be revealing if it is possible to provide seasonally disaggregated optimized crop reallocations. Although the crops like maize and rice are cultivated in both kharif and rabi seasons in few states, seasonal typology is generally maintained by other crops. Figure 2 shows reallocation of wheat area to alternate crops- jowar, bajra, and ragi, typically grown in different seasons. Similarly, some area from rice has been reallocated to wheat which is counter-intuitive as in majority of wheat (rabi) growing states, rice (kharif) is not a competing crop. A clear seasonally-disaggregated picture on crop substitution between competing crops would provide a better comprehension of the results. At least, districts witnessing such counter-intuitive reallocations shall be demarcated.

• As calorie content of selected crops is almost similar (see Supplementary table 4), imposing restriction of ‘calorie production’ in the model for the sake of food security becomes redundant. However, alternate crops viz. jowar, bajra, ragi, maize still offer advantage over rice and wheat in terms of overall nutritional and environmental effects. The authors shall emphasize nutritional security dimension of alternate crops apart from calorie production (food security). This will align the paper with the shifting policy discourse from food to nutritional security in India and globally.

• The scope of the analysis is limited to cereals crops and surprisingly excludes the nitrogen-friendly pulses/leguminous crops. The ability of pulses to fix atmoshpheric nitrogen naturally shall make these crops a preferred element of the alternate crops sub-set, particularly in the studies like this where nutrient management is a core investigation. Also, farmers’ decision

on crop choices are not mutually exclusive to crop groups (eg cereals, pulses, oilseeds, fruits, vegetables). Depending on factors like resources constraints, relative profitability, risk perception etc, farmers often grow a combination of different crops. The authors can discuss the importance of pulses in nitrogen-management and possibly mention the reasons for choosing only cereals crops over similar or even better alternatives like pulses. Future work plan on leveraging potential of pulses in nitrogen management can be outlined.

• It is not clear how biological nitrogen fixation (BNF) for cereals (non-leguminous) crops have been estimated as these crops do not nodulate and data on crop-specific application of biofertilizer at district/state level is not available in cost of cultivation surveys used in the study. The supplementary figure 1 and text (see line 555) mentions the example of legume crops which is excluded from the analyses). For the sake of clarity, it is desirable to provide a supplementary table on BNF estimates for selected crops, as given for other variables.

• The authors may provide source of State-specific fractions of residue brunt in Supplementary table 6. This would be useful for reader as such data is often not widely known.

Overall, the paper provides useful evidences and contributions to the existing literature and can be considered for publication after incorporating the necessary suggestions.

(Remarks on code availability)

Version 2:

Reviewer comments:

Reviewer #6

(Remarks to the Author)
The authors have positively responded to the suggestions and the revised manuscript seems an improvement over the earlier one.

(Remarks on code availability)

Open Access This Peer Review File is licensed under a Creative Commons Attribution 4.0 International License, which permits use, sharing, adaptation, distribution and reproduction in any medium or format, as long as you give appropriate credit to the original author(s) and the source, provide a link to the Creative Commons license, and indicate if changes were made.
In cases where reviewers are anonymous, credit should be given to 'Anonymous Referee' and the source.
The images or other third party material in this Peer Review File are included in the article's Creative Commons license, unless indicated otherwise in a credit line to the material. If material is not included in the article's Creative Commons

license and your intended use is not permitted by statutory regulation or exceeds the permitted use, you will need to obtain permission directly from the copyright holder.
To view a copy of this license, visit https://creativecommons.org/licenses/by/4.0/

Response To Reviewer #2

> **The manuscript presents several noteworthy results. The nitrogen-focused crop restructuring strategy significantly reduces nitrogen surplus by 16–24% and water use by 20–40%. Additionally, it improves farmer income, calorie production, and mitigates greenhouse gas emissions by 28% (113 Mt CO_2 eq). The strategy is shown to be five times more efficient in delivering cross-benefits compared to water-focused restructuring strategies, resulting in a decrease in socio-environmental costs associated with nitrogen pollution by approximately $1.97 billion USD. Furthermore, the restructuring leads to a 4.3-fold increase in the volume of alternative crop trade in the interstate trade network.**

Response: We thank the reviewer for explicitly recognizing and summarizing our key findings and contributions. We have ensured that these results are clearly articulated and emphasized in the revised manuscript.

> **The work is highly significant to the field of agricultural sustainability and related fields such as environmental science and economics. It provides new insights into the benefits of nitrogen management in crop restructuring, which could influence future agricultural policies and practices. Compared to the established literature, this work offers a more comprehensive analysis of the environmental and economic co-benefits of nitrogen-focused strategies, highlighting its superiority over water-focused approaches.**

Response: We greatly appreciate the reviewer's recognition of the significance and potential policy impact of our study. We have carefully reinforced this comparative advantage over water-focused strategies clearly in the revised manuscript, ensuring the implications for agricultural policies are explicitly highlighted. (see Lines 364 –368)

> **The manuscript appears to be original in its approach and findings. It uniquely combines nutrient management with trade network implications, which is not extensively covered in existing literature. If there are similar works, relevant references should be provided to contextualize the novelty of this study.**

Response: We sincerely thank the reviewer for highlighting the originality of our integrated nutrient-trade network approach. To address the reviewer's recommendation explicitly, we have now included additional relevant references from international literature, explicitly positioning our study's novelty within the context of existing global research. Specifically, we have added relevant citations and discussion clearly in the revised Introduction and Discussion sections. (see Lines 26–38)

> **The conclusions and claims made in the manuscript are well-supported**

by the data presented. The evidence provided is robust, though additional data on long-term impacts and regional variations could further strengthen the claims.

Response: We thank the reviewer for recognizing the robustness of our results. In line with the suggestion, we have revised the *Discussion* to explicitly acknowledge the value of longer time series and region-specific analyses for testing persistence and regional heterogeneity of impacts. Our analysis already leverages the most recent, spatially and temporally resolved datasets available (detailed in the Method part). For examaple, fertilizer and manure inputs are drawn from the Government of India *Cost of Cultivation Surveys*Government of India, 2017 and vary across states/districts and years. We now visualize this heterogeneity in a new Supplementary Figure S14 showing spatial distribution of the farmer survey across India and provide additional detail on data sources and uncertainty treatment in the method section. We also report expanded uncertainty assessments that propagate spatial/temporal variability in inputs through our estimates (see Methods line 812-830 and Supplementary Figures S7-S11). These clarifications and analyses have sharpen the scope and interpretation of our findings without altering the main conclusions.

Described method for calculating the net input of nitrogen (N) and phosphorus (P) application rates is comprehensive, there are potential shortfalls to consider. The accuracy of the results heavily depends on the reliability and consistency of the data sources, and any inaccuracies or inconsistencies could lead to erroneous calculations. Additionally, the method may not fully account for regional variations in agricultural practices, soil types, and climatic conditions, which can significantly influence N and P application rates and their effectiveness. Temporal changes in agricultural practices and environmental conditions might not be adequately captured, potentially leading to outdated or less relevant results. Estimating contributions from biological fixation and atmospheric deposition can be challenging and may introduce uncertainties. Furthermore, farmers' survey data might have limitations such as recall bias, incomplete responses, or sampling errors, affecting the overall accuracy of the input data. Addressing these potential shortfalls would involve ensuring high-quality, up-to-date data, considering regional and temporal variations, and carefully estimating contributions from all sources.

Response: We thank the reviewer for this insightful and thorough comment, highlighting several critical considerations. We fully acknowledge that the accuracy and robustness of our results depend substantially on the quality, consistency, and granularity of input data sources, as well as the methodologies employed in estimating nutrient application rates. In particular, we explicitly acknowledge the challenge of adequately capturing the substantial regional variations in agricultural practices, diverse soil types, and varying climatic conditions across India. These regional differences can significantly influence the efficiency and effectiveness of nitrogen (N) and phosphorus (P) applications, making generalizations inherently challenging. Moreover, temporal changes in agricultural practices such as shifts in fertilizer management, adoption of newer crop cultivars, changes in irrigation practices, or alterations due to evolving policy interventions, further complicate the accuracy of longitudinal nutrient application estimates. We have addressed these challenges in the revised manuscript. We also agree fully with the reviewer regarding the inherent uncertainties in estimating biological nitrogen fixation and atmospheric deposition, given the complexity and limited availability of granular data at district levels across India. In the revised

manuscript we explicitly acknowledge and quantify these uncertainties in the manuscript through a careful sensitivity analysis (Supplementary Figures S10-S11), clearly indicating how they influence our results and conclusions.

Regarding farmers' survey data, we recognize potential biases introduced through factors such as recall inaccuracies, incomplete or inconsistent responses, and sampling errors. We have articulated these limitations in the revised manuscript, explicitly highlighting these as potential sources of uncertainty and error. To further contextualize the reviewer's comment, we explicitly note that these challenges are particularly acute in the context of Indian agriculture, characterized by complexity arising from highly diverse crop choices, heterogeneous cropping systems, wide-ranging regional variations in climate and soils, differing state-specific agricultural subsidies, and region-specific minimum support price (MSP) structures. Recognizing that these complexities render accurate data collection and that robust generalizations across regions are inherently difficult, we emphasize the need for careful interpretation of modeling results. To this end, we have explicitly included comprehensive sensitivity analyses in our revised manuscript.(see Lines 389-400)

Overall, in response to this thoughtful critique, we have now provided an explicit and comprehensive discussion of these data limitations, clearly acknowledging the inherent challenges, transparently communicating their implications, and identifying avenues for future improvements in data collection and analytical methodologies. We sincerely appreciate the constructive feedback, as they has significantly strengthened the clarity, balance, and robustness of our manuscript.

Exchange rate and deflation methodology (from the base year) has not mentioned. Manuscript has not organised properly (research gap, limitations of the study are missing and after introduction directly it went to results).

Response: We thank the reviewer for this clarification. We have detailed our exchange rate and deflation methodology, indicating that monetary values are expressed in constant-2015 USD using CPI deflators and annual average exchange rates. This is now explicitly clarified in the Methods section (see Lines 864–871).

We acknowledge the comment on structure, however, the manuscript follows the Nature Portfolio author guidelines and includes all required reporting elements.

The methodology is sound but would benefit from a more detailed description of data sources and methods for reproducibility. Sufficient detail is provided, but specific data sources and assumptions should be clarified further.

Response: We thank the reviewer for recognizing the soundness of our methodology and agree that clearer documentation strengthens reproducibility. In the revision, we have expanded the *Methods* section to describe each dataset's provenance, spatial–temporal coverage, versions/DOIs, and all pre-processing and quality-control steps (see Supplementary Table 1; clarified assumptions and parameter choices with brief rationales and consolidated source information in Supplementary Tables 1–7, which now report definitions, units, resolution, study period, applied transformations and associated assumptions)(see Lines 877–884). By providing this information, along with the source codes and analysis datasets, we strengthen the transparency and reproducibility of our study and facilitate future replication and extension of our work.

The manuscript is a valuable contribution to the field and, with minor revisions, should be suitable for publication. The authors are encouraged to address the highlighted issues to improve the manuscript's overall quality.

Response: We sincerely appreciate the reviewer's strong endorsement and valuable recommendations. All highlighted concerns have been carefully and explicitly addressed, and we believe the revisions have significantly improved the overall quality, clarity, and rigor of the manuscript.

Response To Reviewer #3

We sincerely thank Reviewer 3 for their excellent and constructive critique, which has greatly improved the quality of our manuscript. We have carefully considered each of the comments and have made every effort to address them in full. In particular, we have incorporated India-specific Tier III emission estimates from the BUR-3 report (MoEFCCC), performed additional sensitivity and uncertainty analyses to strengthen the robustness of our findings, and revised the framing to clearly emphasize the novelty of our study in both the global and national context. Point-by-point responses to all comments, both major and minor, are presented below.

> **The manuscript by Goyal et al. quantifies the environmental co-benefits of nitrogen-based crop restructuring and its implications on the trade network for India. The manuscript is well-written and self-explanatory. However, although the title does not mention the geographical area studied and the abstract remotely mentioned global extrapolation, albeit by default, it is essentially a country-specific analysis. In that backdrop, the global context is missing as the data is not extrapolated to a global budget.**

Response: We thank the reviewer for this excellent comment. We agree that the global context of the work needed to be emphasized more clearly. In response, we have substantially revised the Introduction to highlight the broader applicability of our framework while presenting India as the demonstration case. India offers a particularly relevant example because it combines multiple socio-technical and environmental challenges: fertilizer-driven nutrient pollution, widespread pressure on water resources, large-scale agricultural subsidies and procurement systems, and the need to balance food security with environmental sustainability for a population that constitutes 17% of the global total. These factors not only make India globally significant in its own right, but also provide insights that are transferable to other regions facing similar pressures. We also appreciate the reviewer's point about extrapolation to a global budget. To avoid dilution through coarse generalization, which risks masking heterogeneity in agroecological and institutional settings, we have intentionally focused on India as a large and diverse domain.(see Lines 15-20) This allows us to retain analytical depth while still making clear that the framework is globally relevant and can be re-parameterised to other regions confronting coupled nutrient, water, and trade challenges. This framing is now explicitly reflected in the revised Introduction. We have updated the manuscript title to make the India-focused scope explicit: "Quantifying Environmental Co-Benefits of Nitrogen-Based Crop Restructuring and Its Implications on India's Interstate Trade Network."

Considering the subject matter of this manuscript, the element of novelty is missing as several such studies have been conducted worldwide with focused global analysis including India scenario as well. Due to specific analysis based on data originating from India, as has been done in this manuscript, the global appeal of the manuscript gets restricted. This is evident from the references cited in the manuscript and literature available.

Response: We greatly appreciate this critique and understand the perspective of the reviewer. We have carefully revised the manuscript to more clearly highlight the distinct contributions of our study, which can be summarized in three ways. First, we develop a dual objective optimization framework that simultaneously reduces nitrogen surplus and water demand, explicitly quantifying the cross-benefit frontier between them. Our results show that nitrogen-focused restructuring consistently achieves much larger co-benefits, about fivefold higher efficiency than water-focused strategies, while still sustaining calorie production and farmer incomes. Second, instead of treating trade flows as an external outcome, we embed interstate trade directly within the optimization framework, which is constrained by calorie and income requirements. This approach shows that nitrogen-based restructuring increases the capacity of alternative cereal trade more than four times. Our analysis therefore explores the linkages between nutrient management and trade network dynamics in a manner that has not been examined previously. Third, our framework integrates multiple environmental and social outcomes, including greenhouse gas emissions, nitrogen leaching, monetized damages from nitrogen pollution, and cultural constraints related to rice and wheat consumptions. Taken together, our work addresses a body of research that is still largely fragmented, with separate studies focusing either on nutrient management Gu et al., 2021, 2023; Ren et al., 2022, water conservation Chakraborti et al., 2023; Davis et al., 2017, 2018, 2019, environmental impacts Beyer et al., 2022, or trade restructuring **liu2024china**; **tang2017will**. By consolidating these dimensions within a single analysis framework, we provide an integrated perspective that extends the global literature and demonstrates how cross-objective benefits can be realised in practice.

To address the reviewer's remarks, we have also extensively revised the Introduction to ensure that the global applicability of our framework is communicated clearly, while at the same time emphasising the merit of using India as an exemplar. India's scale, diversity, and distinctive policy environment make it both a globally significant case in its own right and a valuable testbed for approaches that can be adapted to other regions. To ensure these contributions are visible to readers, we have added explicit statements at the end of the Introduction and reiterated them in the Discussion. We have also strengthened the literature review by including key international works as suggested by the reviewer . With these revisions, we aim to position our work as complementary to existing studies, while clearly articulating the precise gap it addresses and highlighting the essential novel aspects of our analysis.

"Recent global analyses highlight the promise of targeted crop switching and redistribution to mitigate irrigation demand X. Chen et al., 2014; Davis et al., 2017; Kuang et al., 2022; Shi et al., 2024; Wang et al., 2022; Wen and Chen, 2023; Xie et al., 2023, conserve fertilzer Ren et al., 2022; You et al., 2024, and enhance biodiversity Beyer et al., 2022 while maintaining or increasing production. Yet much of this literature treats objectives in isolation, providing limited insight into cross-objective trade-offs Breure et al., 2024 and co-benefits across water, nutrient, and climate outcomes, and it rarely embeds these strategies within national and international trade systems to assess their distributional, social, and environmental consequences. With its burgeoning population, India has been at the forefront of efforts to restructure its Green Revolution–era agricultural system; however,

studies in the Indian context have predominantly focused on minimizing water consumption and alleviating groundwater stress Chakraborti et al., 2023; Davis et al., 2018, 2019; Devineni et al., 2022; Wei et al., 2025, while the equally critical challenge of reducing agricultural nitrogen pollution remains comparatively underexplored."(see Lines: 26—38)

In addition, nitrogen impacts need to be considered along with water relations and there is an element of synergy among the two factors influencing crop productivity. Although, the authors did refer to such relationship, they did not make attempt for such analysis and exclusively focussed on N relations only.

Response: We thank the reviewer for drawing attention to the critical linkages between nitrogen and water. We respectfully clarify that our analysis does not exclusively focus on nitrogen; rather, we explicitly evaluated nitrogen–water interactions using a dual-objective optimization framework. In the revised manuscript, we highlight this aspect more clearly. Specifically, Figure 2a presents the pareto front that jointly minimizes nitrogen surplus and water demand, directly quantifying the trade-offs between the two. Our results show that nitrogen-focused restructuring yields substantial water savings in addition to lowering nitrogen surplus, while water-focused restructuring delivers comparatively weaker nitrogen (surplus) reductions. We further quantify this synergy through a cross-benefit ratio, demonstrating that nitrogen-first strategies are approximately five times more efficient in generating co-benefits than water-first strategies. To ensure that these results are more visible to readers, we have revised the Results part of the manuscript (Lines 158–161). We emphasis the nitrogen–water pareto analysis and have clarified in the Discussion that one of the key novelty of our analysis is in unifying nutrient and water management rather than treating them in isolation (Lines 363–368). We have also included appropriate references to the revised texts related to water-focused restructuring scenarios and have further elaborated on the corresponding trade network implications .(see Lines 72–75)

"While previous studies have focused on the redistribution of the Indian agricultural system with an emphasis on water-saving approachesChakraborti et al., 2023, our findings suggest that nitrogen surplus reduction not only reduces nutrient pollution but also unlocks substantial water savings and co-benefits"(see Lines: 363—368)

The analysis based on a linear algorithm, originally envisaged several objectives including crop restructuring, environmental trade-offs and inter-state trade, and should have involved multi-nodal analytical algorithms.

Response: This is a great point, and we fully agree that multi-nodal analytical frameworks are powerful tools for capturing complex, dynamic interactions in agricultural systems. At the same time, we note that our analysis already incorporates a multi-nodal structure, albeit within a tractable linear programming (LP) framework. We include a multitude of diverse information in our framework wherein district-level crop allocations are optimised subject to state-level calorie and income constraints, and interstate trade flows are endogenously updated to ensure importing states meet caloric requirements post-restructuring. This effectively encodes nodal coupling across multiple states and crops within the LP formulation. We chose this approach deliberately: it provides transparency, computational feasibility, and tractability when dealing with sub-national datasets with clear policy relevance aspects by producing interpretable trade-offs that can inform procurement and subsidy decisions. More elaborated multi-nodal or nonlinear frameworks would

require highly granular, harmonized datasets on farmer behavior, crop adoption dynamics, and inter-state trade frictions, which are currently unavailable at the national scale in India.

Restructuring the Indian agricultural system is an exceptionally complex task, marked by diverse crop preferences, socio-cultural factors influencing dietary choices, regionally varying agricultural subsidies, heterogeneous minimum support price (MSP) structures, and substantial policy variation across states. At the resolution of our study, i.e., district (sub-national) level data encompassing variables such as cropping area, production, yield, water footprint, synthetic fertilizer and manure application, atmospheric deposition, biological fixation, calorie production, agricultural GHG emissions, and MSP, implementing a multi-objective, non-linear optimization framework would necessitate solving an exceptionally large system of equations. Given this scale, and our focus on cropland restructuring, a linear optimization model provided a transparent, computationally tractable, and policy-relevant starting point without introducing additional algorithmic complexity. This approach is also consistent with previous literature Beyer et al., 2022; Wei et al., 2025, which applies linear optimization to comparable large-scale agricultural systems. We view this as a practical first step that can be extended in future work toward more advanced multi-nodal or non-linear analytical frameworks once computational and data-integration challenges are addressed.

To clarify further this aspect, we have revised the Methods to explicitly describe how nodal interactions are embedded in the LP formulation, and we added a note in the Discussion acknowledging that future work could extend our framework by incorporating dynamic feedback and behavioral mechanisms as improved datasets become available.

N-pollution and global health analysis has also been a subject of much research in China, Germany, Netherlands and the Climate Analysis Group of FAO, with several interdisciplinary models having been developed (see the publications of Dr. Baojing Gu). Thus, going by the objectives stated by the authors, this analysis gap is evident.

Response: We thank the reviewer for this insightful comment and clearly acknowledge the substantial body of global research on nitrogen pollution and health impacts, including the influential work of Dr. Baojing Gu and colleagues. Previous studies have been invaluable in shaping the understanding of nitrogen budgets and their environmental and health consequences, and we have duly cited them in our manuscript to situate our work within this broader literature.

However, these studies have developed models to primarily analyze the fate of nitrogen Houlton et al., 2019; Ren et al., 2022 in agricultural systems and often do not incorporate the trade-off and interaction of water with nutrients and their subsequent impacts on trade systems. Our study, however, was not intended to replicate global analyses but to address a specific gap to exemplify water-nutrient interactions and implications on inter-India trade systems. The first contribution of our work is the explicit integration of nitrogen and water objectives in a single optimisation framework. By analyzing them together through a dual-objective pareto approach, we show that nitrogen-focused restructuring not only reduces surplus but also delivers larger water savings than water-focused restructuring. The cross-benefit ratio further demonstrates that nitrogen-first strategies are about five times more effective in producing water savings compared to the reverse. This unified treatment of nitrogen and water goes beyond much of the existing literature, which tends to consider them separately Chakraborti et al., 2023; Davis et al., 2017, 2018; Ren et al., 2022.

The second contribution, central to our study, is the integration of crop restructuring with interstate trade flows under calorie and income constraints. This design ensures that trade flows are updated endogenously in response to restructuring, rather than treated as an external outcome. Our analysis reveal that nitrogen-based restructuring alters both the composition and the volume

of interstate trade, with alternative cereal trade increasing more than fourfold while preserving caloric sufficiency for importing states. To best of our knowledge, no previous study has brought nutrient management and trade network restructuring together in this way.

India also presents a uniquely important setting for such an analysis as discussed above. Beyond its global importance as a major producer and exporter of cereals, its agricultural system is shaped by distinct policy instruments such as procurement schemes, fertilizer subsidies, and minimum support prices. These institutional features play a critical role in farmer decision-making and cannot be meaningfully represented in global average models. A dedicated national analysis is therefore necessary to capture the combined effects of nitrogen management, water conservation, and trade restructuring within this context.

To make this positioning clearer, we have revised the manuscript at several places. The Introduction now ends with an explicit statement of novelty. The Discussion part includes a new paragraph situating India as a globally significant testbed and clarifying that our framework is transferable to other regions through re-parameterization rather than extrapolation. Finally, the Conclusion reiterates that our work fills a gap by linking nutrient management, water conservation, and trade restructuring in one unified analysis.(see Lines: 386–400)

> **Abstract line 14:** Delete “into their strategies”.

Response: Implemented as suggested: we removed the phrase ’into their strategies’ from the Abstract.

> **Keywords:** Add “system efficiency”.

Response: Done: we added “system efficiency”to the keyword list.

> **Line 1 : Replace “in” with “for” Line 9 : Change to “emphasis on high-yielding crop cultivars” Line 17 : Replace “rice and wheat” with “cereals” Lines 18–20 : Change to “mainly N-fertilizers leading . . . and overuse of water resources . . . ” Line 23 : Change to “all the while”**

Response: Thank you for these suggestions. We have implemented them in the revised manuscript. For example: Line 1 (“in” → “for”); Line 9 (“emphasis on high-yielding crop cultivars”); Line 22 (“rice and wheat” → “cereals”); Lines 23–25 (“mainly N-fertilizers leading . . . and overuse of water resources . . . ”); and Line 23 (“all the while”). These changes are reflected in the revised manuscript (now at Lines 2, 9, 22, 23-25, and 27-38).

> **Line 50 : Not all nitrogen has environmental impact; introduce reactive-N (Nr).**

Response: We thank the reviewer for this helpful clarification. We have now explicitly introduce *reactive nitrogen (Nr)* at its first mention (Line 65) to distinguish environmentally active nitrogen compounds from inert N_2, and we use *Nr* consistently thereafter when discussing environmental effects (e.g., surplus, leaching, and gaseous losses). Concretely, phrases such as “nitrogen losses” have been revised to “*reactive nitrogen (Nr) losses*” (e.g., “potential Nr losses to the environment”). We also added a definitional sentence: “*Throughout, ‘nitrogen’ refers to reactive nitrogen (Nr) — biologically, chemically, and radiatively active nitrogen containing compounds (e.g.,* NH_3/NH_4^+, NO_3^-, NO_x, N_2O*); molecular* N_2 *is excluded.*” Corresponding edits have been made at relevant places: main text, figure captions, and axes.

Line 53 : Change to "environmental and biological". Line 55 : Use Nr consistently. Line 59 : Ref 39—consider Lassaletta *et al.*

Response: Thank you. We have revised the wording as suggested (Lines 61,49,66), and included the citation to Lassaletta *et al.* (Ref. 44).

Line 73 : "minimization" → "reduction" Line 75 : AGHG should be "agricultural greenhouse gas"; add to Abbreviations. Line 99 : "eccentric" – perhaps "centric" or "dependent"?

Response: All terms have been updated as suggested, including"AGHG" being now defined in the Abbreviations list.

Line 127-128: This statement may not be true. Maize, especially the double-cross hybrids, are high yielding, as are modern finger millet varieties. Maize has high yield potential, but in India, 60% is used as animal feed and only 13% as food.

Response: We thank the reviewer for highlighting this important point. We fully agree that maize, particularly with the adoption of modern double-cross hybrids, possesses very high yield potential, and that improved varieties of finger millet have also demonstrated considerable productivity gains. Our original statement was not intended to suggest otherwise. Rather, our analysis is grounded in the observed baseline yields and nutrient use efficiencies from 2017 across Indian districts, which form the empirical basis for our restructure analysis. In this dataset, rice and wheat dominate caloric production in the Indo-Gangetic Plains, not because maize and/or millet lack biological potential, but because realized yields per unit nitrogen applied (nitrogen use efficiency) are higher for rice in the baseline allocation. To this end, we have revised the text (Lines 143–152) to make it clearer that our comparative analysis, supported by available statistics (ICRISAT/official database), focuses on yield per unit nitrogen input rather than absolute yield potential.

We also note the reviewer's valuable point on the end use of maize. While much of India's maize production is channeled towards animal feed, our analysis framework is structured to preserve caloric sufficiency for human consumption by imposing calorie and income constraints at the state level. This ensures that trade restructuring and crop diversification do not come at the expense of food security. We have revised the manuscript to explicitly clarify these distinctions: that our conclusions are based on observed nutrient efficiencies and that they should not be interpreted as disregarding the high yield potential of modern maize and millet hybrids; and human caloric sufficiency is safeguarded within the analysis framework (Lines 397–399).

"In addition, our analysis does not differentiate crop genetic varieties, which can vary in water- and nutrient-use efficiency and thereby affect projections of productivity and environmental impacts."

Lines 152–153: The reported water savings may be underestimated. Basmati rice varieties with improved water-saving technologies have achieved yield stability and enhanced water use efficiency.

Response: We thank the reviewer for this insightful point and agree that variety- and practice-specific water savings (e.g., improved Basmati cultivars under water-saving management) can exceed our system-level estimates. Our estimates deliberately reflect conservative, system-average conditions because nationally consistent, district-resolved data on cultivar mix and the adoption of water-saving technologies are not yet available. We have recognized these aspects in the *Discussion*

(revised Lines 392–394) that our water metric represents consumptive demand under representative practices. The reported savings can be viewed as first-order estimates and should be interpreted with caution, as they will vary with local policies and management. Wider adoption of water-saving technologies would likely shift the observed trade-off curve outward, amplifying water reductions without compromising yield stability. We now explicitly highlight these improved water-saving aspects and identify the integration of cultivar- and management-level adoption data as an important direction for future refinement. With respect to the current work, we emphasize that our comparative analysis of restructuring based on nutrient and water savings consistently applies the same water-demand assumptions for a given crop.

Fig. 2: Virtual export of water and nitrogen through agricultural export (both intra- and inter-country scenarios) has been studied. Please include relevant references in the Discussion and elaborate on further reduction via crop replacement and trade restructuring.

Response: We appreciate this insightful suggestion. The revised Discussion section (Lines 379–383) now explicitly incorporates relevant literature on virtual water and nitrogen trade, placing our findings within a broader context and discussing potential further reductions achievable through crop restructuring and trade network adjustments. References have been explicitly included (Lines 72–75).

" *Furthermore researchers have extensively quantified virtual water* **mekonnen2024trends***; Harris et al., 2020 and nutrient footprints X. Chen et al., 2023; Goyal et al., 2024; Oita et al., 2016 in within-country and cross-border trade flows, it has seldom evaluated how agricultural restructuring (e.g., crop reallocation) would reconfigure trade systems* **liu2024china***;* **mehla2023optimization"**

Line 186 : "N-centric"? Line 235 : Cultural consideration should include socio-economic perspective. Line 239 : Replace "quick" with "easy".

Response: Thanks. "*N-centric*" has been updated to "nitrogen-centric" (Line 112). Socio-economic dimensions have been integrated into the cultural discussion, and "quick" has been replaced with "easy".

Uncertainty analysis and its mitigation should be analyzed quantitatively and integrated in the model. It is currently mentioned only as 'constraints' (Line 554), which requires further clarification.

Response: We fully agree and have conducted an explicit quantitative sensitivity and uncertainty analysis on key parameters, including fertilizer use rates, nitrogen deposition, and biological nitrogen fixation. Results from this analysis are presented in the revised manuscript (see e.g., supplementary Figure S 7-11 and Methods section at Lines 873–890). We now explicitly account for uncertainties in key parameters propagated through our analytical framework, thereby addressing your valuable suggestion.

Line 477 : N-surplus must include N-leakage. Line 516 : Reference "*Agricultural Statistics at a Glance*".

Response: Thank you for these remarks. We have added a dedicated N_{leak} term to Eq. 6 and quantify N-leakage estimates with region-specific coefficients.(see supplementary Figure 15)

- **Data source:** "*Agricultural Statistics at a Glance*" is now cited explicitly (Line 518).

Line 526 : Convert all monetary values to USD for an international audience.

Response: All monetary quantities have been converted to *constant-2015 USD*. Values were first deflated to 2015 using CPI series and then converted to USD using the corresponding annual average exchange rates (Lines 852–859). Damage cost factors per kg of reactive nitrogen (Nr) follow Sutton et al. Sutton et al., 2017. A summary of 2015 Nr loss cost estimates (USD kg^{-1} N) is provided in Supplementary Table S5.

Line 580 : Limitations should reflect in the model.

Response: A dedicated "Limitations" subsection (Lines 386–413) outlines how data and modeling constraints were taken into account in optimization have been added in the revised manuscript.

We acknowledge the complexity of Indian agriculture and the limitations of our study. While our nutrient-centric approach is grounded in the best available national-scale data, greater spatial and temporal disaggregation of fertilizer, manure, and socio-economic information would allow more targeted interventions. The transition towards nutrient-focused restructuring must also consider local food demand, the structure of farm holdings and cooperatives, procurement policies, rationing systems, and minimum support prices (MSPs), all of which influence adoption feasibility. Several parameters used to identify optimal strategies remain uncertain due to climatic variability and regional differences in agricultural practices and policies. We addressed part of this uncertainty through bootstrapping and sensitivity analyses (see Methods; Supplementary Figure. S7–S11), but more detailed datasets would strengthen parameter robustness..... (lines 375–393)

Line 687 : It is better to use Indian data reflected in the BUR-3 report published by the Ministry of Environment (MoEFCCC), Govt. of India. Now India already has Tier III dataset which could refine the estimate and incorporated in the model.

Response: We thank the reviewer for this valuable suggestion. We have updated our AGHG calculations following the BUR-3 Tier III dataset for India (Lines 713–758). This revision enables the use of spatially explicit emission factors, incorporating greenhouse gas emissions from crop residue burning, methane emissions from rice cultivation, and emissions from manure and fertilizer management. The BUR-3 Tier III datasetDhingra et al., 2019 provides a methodological framework for emission factors for multiple pathways, which we integrated into our spatial model to generate crop-specific emission intensities at high resolution. This refinement captures heterogeneity in agricultural practices and climatic conditions across India, thereby reducing the aggregation bias inherent in earlier IPCC Tier I-based estimatesCarlson et al., 2017. We are grateful for this comment, as it has helped us improve our uniform Tier I approach to a more robust, spatially varied Tier III framework informed by the Indian BUR-3 report. We have added a Supplementary Table 7-9 listing all the factors and datasets used. Further to this we have added the supplementary Figures S12-S13.

Line 700 : Nitrogen loss cannot be a default value for a large country.

Response: This is a valid point. In the revised manuscript, we have clarified that nitrogen loss coefficients are derived from simulations using the well-recognized gridded IMAGE–GNM model

Beusen et al., 2015. Both the nitrogen leaching and N_2O emission factors are spatially explicit and geographically varying, rather than being applied as a single national default. We are aware of the uncertainties associated with applying a global model at this scale; however, IMAGE–GNM has been widely used for evaluating nitrogen fate worldwide and has also been applied in previous studies focusing specifically on India Elsayed et al., 2025. To better reflect India's heterogeneity, these coefficients were explicitly regionalized, and the spatial variability of nitrogen loss factors is now presented in Supplementary Figure S15.

Moreover, the nitrogen leaching and emission coefficients derived from the global IMAGE–GNM model are constrained by coarse spatial resolution and generic parameterization, which may not fully capture the heterogeneity of Indian agroecosystems. (line 399–402)

General : How was the data retro-fitted onto the map of India?

Response: Crop data were rasterized using Python geospatial libraries to ensure accurate alignment with district-level boundaries. We once again thank the reviewer for their thoughtful and constructive critique that helped us to improve our manuscript. The revisions prompted by your comments have significantly strengthened the scientific rigor, transparency, and broader relevance of our work.

Response To Reviewer #4

We sincerely thank Reviewer 4 for their insightful comments, which have significantly enhanced the clarity and impact of our manuscript. Below, we provide detailed line-by-line responses to each of the comments raised.

General Assessment

> **The paper is methodologically sound and substantiates the case as to why nutrient-centric agricultural restructuring could make sense in India. The use of a multi-objective optimization model to evaluate trade-offs and co-benefits of minimizing nitrogen surplus and water use was able to explain the implications for sustainability, food security, and interstate trade. It's also notable that the authors considered the socio-economic and cultural context in their methodology.**

Response: We thank the reviewer for this encouraging assessment and are pleased that the methodological rigor, policy relevance, and consideration of socio-economic context in our study were explicitly recognized.

A. Model Assumptions And Data Sources

> **The authors used IMAGE-GNM emission and leaching factors. However, these are global-scale model outputs and may not accurately reflect India's heterogeneity in soils, rainfall, and management. I suggest to add a brief discussion on this limitation and suggest more localized empirical data or models that could enhance future versions.**

Response: We appreciate this important observation. In the revised manuscript, we have added a discussion acknowledging that the global-scale emission and leaching factors derived from IMAGE-GNM may not fully capture the heterogeneity of Indian soils, climatic variability, and localized management practices. Both the nitrogen leaching and N_2O emission factors are spatially explicit and geographically varying, reflecting underlying landscape heterogeneity (e.g., soil, vegetation and hydroclimatic conditions; see Supplementary Figure S15). Furthermore, more recently Elsayed *et al.* (2025)Elsayed et al., 2025 evaluated soil nitrogen budgets and fates in Indian agriculture using the IMAGE model, providing a basis for our current approach. Nevertheless, we agree that future research could be strengthened by incorporating region-specific empirical datasets and/or applying localized agro-environmental models, which would better capture India's diverse agricultural contexts and provide improved nitrogen (leaching and emission) estimates. We have included these forward-looking aspects in the revised manuscript (at Lines: 399–402).

> **The model included valuable constraints on minimum rice/wheat culti-**

vation due to cultural importance, but adoption barriers like market access, taste preferences, processing infrastructure, and farmer skill adaptation were not fully addressed. Maybe add a brief discussion regarding these practical adoption constraints and how they could be incorporated in future modeling.

Response: We thank the reviewer for this relevant suggestion. In the revised discussion, we have added consideration of practical adoption constraints, explicitly addressing market access limitations, consumer taste preferences, processing infrastructure gaps, and challenges related to farmer skill adaptation. We also outline potential approaches for incorporating these socio-economic and infrastructural factors into future modeling frameworks, underscoring their importance for enhancing the practical applicability and realism of agricultural restructuring scenarios (Lines 405–413).

Future research could extend our framework by integrating state-specific procurement incentives, emerging subsidies, and dynamic pricing—particularly for millets—in domestic and international markets...

Post-optimality sensitivity analysis was undertaken and real-world constraints (i.e., minimum caloric output, no expansion beyond historical crop areas, and farmer profit stability) ground the model in realistic agronomic and economic conditions. However, empirical/external validation was lacking. While this is understandable given the scale and ambition of the work, the policy relevance of the study would be significantly strengthened by empirical comparisons or even qualitative alignment with existing field programs and observed trends.

Response: We fully concur with the reviewer's point regarding empirical validation. Given the scale and complexity of the analysis, direct empirical validation poses substantial challenges. Nonetheless, we have expanded the discussion to include qualitative comparisons with existing agricultural programs and documented trends within India (Lines 394–397). This addition aims to bridge the model results with real-world observations and enhances the policy relevance of our findings.

Embedding the approach in participatory, on-farm validation and iterative policy design can accelerate an equitable transition away from resource-intensive production systems.

Reactive-N (Nr) is defined at first mention (Line 147) and used consistently; "AGHG" is now spelled out and added to the Abbreviations list; "eccentric" is replaced with "crop-centric".

Response: We thank the reviewer for these helpful editorial suggestions. All terminology corrections have been implemented as suggested by the reviewer.

Figure labels and legends are too small and hard to read. Increase size

of plots and text font to make them more legible. Consider breaking up multiple plots (i.e., Fig. 2) if appropriate. "Pareto" was misspelled in Fig. S2. The color of the bar (profit) for Fig. S3b confused me. My sense was red color denoted an increase in percentage. I also suggest dropping the "%" on the labels for the trade-offs on the y-axis on S3 since it was already stated on the x-axis label.

Response: We appreciate these detailed suggestions on enhancing the readability and clarity of presented figures. We have increased the font size for all figure labels and legends, corrected the spelling of 'Pareto' in Supplementary Fig. S2, and adjusted the color scheme in Fig. S3b for clarity. Additionally, we have removed redundant '%' symbols from the labels on the y-axis of Fig. S3, ensuring consistency and avoiding potential confusion. Once again, we sincerely thank Reviewer #4 for their thorough and constructive feedback, which has undoubtedly improved the manuscript.

Final Recommendation

Overall, it is an excellent paper offering quantitative evidence for how nitrogen surplus management can lead to broad environmental and economic co-benefits, especially in the Indian context. I recommend to accept this paper with minor revisions to address the above points.

Response: We are grateful for the reviewer's positive evaluation and encouraging assessment. We are glad that the quantitative evidence and policy relevance of our work were recognized. We have carefully addressed all the suggested minor revisions in the revised manuscript.

References

Beusen, A., Van Beek, L., Bouwman, A., Mogollón, J., & Middelburg, J. (2015). Coupling global models for hydrology and nutrient loading to simulate nitrogen and phosphorus retention in surface water–description of image–gnm and analysis of performance. *Geoscientific model development*, *8*(12), 4045–4067.

Beyer, R. M., Hua, F., Martin, P. A., Manica, A., & Rademacher, T. (2022). Relocating croplands could drastically reduce the environmental impacts of global food production. *Communications Earth & Environment*, *3*(1), 49.

Breure, T. S., Estrada-Carmona, N., Petsakos, A., Gotor, E., Jansen, B., & Groot, J. C. (2024). A systematic review of the methodology of trade-off analysis in agriculture. *Nature Food*, 1–10.

Carlson, K. M., Gerber, J. S., Mueller, N. D., Herrero, M., MacDonald, G. K., Brauman, K. A., Havlik, P., O'Connell, C. S., Johnson, J. A., Saatchi, S., et al. (2017). Greenhouse gas emissions intensity of global croplands. *Nature Climate Change*, *7*(1), 63–68.

Chakraborti, R., Davis, K. F., DeFries, R., Rao, N. D., Joseph, J., & Ghosh, S. (2023). Crop switching for water sustainability in india's food bowl yields co-benefits for food security and farmers' profits. *Nature Water*, *1*(10), 864–878.

Chen, X., Cui, Z., Fan, M., Vitousek, P., Zhao, M., Ma, W., Wang, Z., Zhang, W., Yan, X., Yang, J., et al. (2014). Producing more grain with lower environmental costs. *Nature*, *514*(7523), 486–489.

Chen, X., Hou, Y., Kastner, T., Liu, L., Zhang, Y., Yin, T., Li, M., Malik, A., Li, M., Thorp, K. R., et al. (2023). Physical and virtual nutrient flows in global telecoupled agricultural trade networks. *Nature Communications*, *14*(1), 2391.

Davis, K. F., Chhatre, A., Rao, N. D., Singh, D., Ghosh-Jerath, S., Mridul, A., Poblete-Cazenave, M., Pradhan, N., & DeFries, R. (2019). Assessing the sustainability of post-green revolution cereals in india. *Proceedings of the National Academy of Sciences*, *116*(50), 25034–25041.

Davis, K. F., Chiarelli, D. D., Rulli, M. C., Chhatre, A., Richter, B., Singh, D., & DeFries, R. (2018). Alternative cereals can improve water use and nutrient supply in india. *Science advances*, *4*(7), eaao1108.

Davis, K. F., Rulli, M. C., Seveso, A., & D'Odorico, P. (2017). Increased food production and reduced water use through optimized crop distribution. *Nature Geoscience*, *10*(12), 919–924.

Devineni, N., Perveen, S., & Lall, U. (2022). Solving groundwater depletion in india while achieving food security. *Nature Communications*, *13*(1), 3374.

Dhingra, S., Singh, D., & Mehta, R. (2019, September). *Methodology note: National & state level greenhouse gas estimates, 2005–2015 — agriculture, forestry and other land use sector (afolu)* (Methodology note) (Phase III). Vasudha Foundation; GHG Platform India. New Delhi. Retrieved August 11, 2025, from https://www.ghgplatform-india.org/wp-content/uploads/2023/03/AFOLU-Methodology-Note-GHG-Phase-III.pdf

Elsayed, H., Beusen, A., Prusty, A. K., & Bouwman, L. (2025). Long-term variations (1970–2020) and spatial patterns of nitrogen and phosphorus soil budgets and fates in indian agriculture. *Nutrient Cycling in Agroecosystems*, *130*(1), 17–32.

Government of India. (2017). Agricultural statistics at a glance 2017 [Accessed: 2024-07-16].

Goyal, S. S., Dave, R., Kumar, R., & Bhatia, U. (2024). Indian interstate trade exacerbates nutrient pollution in food production hubs. *Communications Earth & Environment*, *5*(1), 9.

Gu, B., van Grinsven, H. J., Lam, S. K., Oenema, O., Sutton, M. A., Mosier, A., & Chen, D. (2021). A credit system to solve agricultural nitrogen pollution. *The Innovation*, *2*(1).

Gu, B., Zhang, X., Lam, S. K., Yu, Y., Van Grinsven, H. J., Zhang, S., Wang, X., Bodirsky, B. L., Wang, S., Duan, J., et al. (2023). Cost-effective mitigation of nitrogen pollution from global croplands. *Nature*, *613*(7942), 77–84.

Harris, F., Dalin, C., Cuevas, S., Lakshmikantha, N., Adhya, T., Joy, E. J., Scheelbeek, P. F., Kayatz, B., Nicholas, O., Shankar, B., et al. (2020). Trading water: Virtual water flows through interstate cereal trade in india. *Environmental Research Letters*, *15*(12), 125005.

Houlton, B. Z., Almaraz, M., Aneja, V., Austin, A. T., Bai, E., Cassman, K. G., Compton, J. E., Davidson, E. A., Erisman, J. W., Galloway, J. N., et al. (2019). A world of cobenefits: Solving the global nitrogen challenge. *Earth's future*, *7*(8), 865–872.

Kuang, W., Liu, J., Tian, H., Shi, H., Dong, J., Song, C., Li, X., Du, G., Hou, Y., Lu, D., et al. (2022). Cropland redistribution to marginal lands undermines environmental sustainability. *National science review*, *9*(1), nwab091.

Oita, A., Malik, A., Kanemoto, K., Geschke, A., Nishijima, S., & Lenzen, M. (2016). Substantial nitrogen pollution embedded in international trade. *Nature Geoscience*, *9*(2), 111–115.

Ren, K., Xu, M., Li, R., Zheng, L., Liu, S., Reis, S., Wang, H., Lu, C., Zhang, W., Gao, H., et al. (2022). Optimizing nitrogen fertilizer use for more grain and less pollution. *Journal of Cleaner Production*, *360*, 132180.

Shi, W., Wang, M., Tao, F., Xu, X., Deng, X., Liu, L., Kong, X., Zuo, L., Lei, M., Shi, X., & Wang, X. (2024). Wheat redistribution in huang-huai-hai, china, could reduce groundwater depletion and environmental footprints without compromising production. *Communications Earth & Environment*, *5*(1), 380. https://doi.org/10.1038/s43247-024-01547-9

Sutton, M., Drewer, J., Moring, A., Adhya, T., Ahmed, A., Bhatia, A., Brownlie, W., Dragosits, U., Ghude, S., Hillier, J., et al. (2017). The indian nitrogen challenge in a global perspective. In *The indian nitrogen assessment* (pp. 9–28). Elsevier.

Wang, Z., Yin, Y., Wang, Y., Tian, X., Ying, H., Zhang, Q., Xue, Y., Oenema, O., Li, S., Zhou, F., et al. (2022). Integrating crop redistribution and improved management towards meeting china's food demand with lower environmental costs. *Nature Food*, *3*(12), 1031–1039.

Wei, D., Castro, L. G., Chhatre, A., Tuninetti, M., & Davis, K. F. (2025). Swapping rice for alternative cereals can reduce climate-induced production losses and increase farmer incomes in india. *Nature Communications*, *16*(1), 2108.

Wen, M., & Chen, L. (2023). Global food crop redistribution reduces water footprint without compromising species diversity. *Journal of Cleaner Production*, *383*, 135437.

Xie, W., Zhu, A., Ali, T., Zhang, Z., Chen, X., Wu, F., Huang, J., & Davis, K. F. (2023). Crop switching can enhance environmental sustainability and farmer incomes in china. *Nature*, 1–6.

You, L., Ros, G. H., Chen, Y., Zhang, F., & de Vries, W. (2024). Optimized agricultural management reduces global cropland nitrogen losses to air and water. *Nature Food*, *5*(12), 995–1004.

Point-by-point response to reviewers

We thank Reviewer #3 and Reviewer #4 for their positive feedback and for the time they invested in evaluating the manuscript. We thank Reviewer #6 for the detailed and constructive comments. We have fully incorporated these comments, and they have further strengthened the manuscript.

Point-by-point responses to all reviewers are provided below. Reviewer comments are reproduced verbatim in black, our responses are provided in blue, and manuscript touchpoints are summarized in red. Line numbers cited below refer to the current clean revised manuscript and revised supplementary information.

Response to Reviewer #3

Comment

Authors have positively responded to the comments of the Revieweres and made required modifications/changes in the text and also in the supplementary. The article now reads well.

Response

We thank Reviewer #3 for their overall positive assessment of our revisions. We appreciate the reviewer's time and effort in reviewing our manuscript.

Response to Reviewer #4

Comment

I have carefully reviewed the authors' revised manuscript and their detailed responses to my comments. I am satisfied that all of my concerns have been thoroughly addressed, and the revisions have significantly improved the clarity, rigor, and policy relevance of the paper. Overall, I find the manuscript suitable for publication in its current form.

Response

We thank Reviewer #4 for their overall positive assessment of our revisions. We appreciate the reviewer's time and effort in reviewing our manuscript.

Response to Reviewer #6

Response

We thank Reviewer #6 for the careful and in-depth review. These comments substantially improved the manuscript. In response, we incorporated the additional official datasets suggested by the reviewer wherever they could be used directly, updated the primary revenue-benchmark analysis accordingly, clarified the scope of the calorie constraint, added seasonal and uncertainty analyses, and made the remaining benchmark limitations explicit. We respond point by point below.

Comment

Comments on "Quantifying environmental co-benefits of nitrogen-based crop restructuring and its implications on India's interstate trade network".

- Using multi-objective optimization model, the paper has developed a framework and demonstrated the superiority of nitrogen-focussed crop restructuring strategy in lowering nitrogen surplus along with producing water-saving over water-focussed restructuring, while maintaining the calorie production and farmers income. The model also uniquely integrates crop restructuring with inter-state trade flow for ensuring compensation of potential calorie deficits from crop restructuring through increased trade volume of alternative crops. Overall, the paper provides useful insights on leveraging the synergy between nutrient and water management and aligns with the national and global priorities on sustainable agriculture.

Response

We thank the reviewer for this overall positive assessment of our work and for recognizing both the optimization framework and the integration of crop restructuring with interstate trade. The detailed revisions described below were undertaken to further sharpen the basis, scope, and presentation of these core findings.

Comment 1

Comment

To reflect a realistic agricultural scenario, crop area optimization has been implemented with constraints on calorie production and farmers' income. The farmer's income has been estimated at the district level by deducting production costs from sale revenue. The authors have used national-level minimum support price (MSP) for estimating sale revenue of crops at district level. Valuating production with administratively fixed MSP does not represent realistic agricultural market because MSP is primarily designed as a safety net mechanism rather than reflecting a true value of produce at competitive market. Further, it is characterized with limited access (only 15% of paddy farmers and 9.6% of wheat farmers availed MSP in 2018-19) with significant variation across crops and geographical regions. MSP-based procurements are biased towards crops like paddy and wheat, while the alternate crops in the study (maize, jowar, bajra and ragi) are largely excluded from MSP-based procurement. Using MSP for estimating income, therefore, masks inter-crop disparity in actual price realization and produces inconsistent revenue terms of trade between crops- a key factor driving farmers' decision on crop choices. Consequently, the optimization results may not reflect real market scenario. A simple alternative to MSP could be 'unit-prices' for each crop – derived by dividing value of crop output with production. Beside representing actual average prices realized by farmers for crop produce, unit-prices can be estimated at state level and thus provide more variability in data than using single national-level MSP.

Response

We thank the reviewer for this clear and well-structured clarification, which has significantly strengthened our analysis by grounding the work more firmly in real-world conditions. In response, we have revised our work, considering now the revenue benchmark using state/crop-specific official value-of-output and production statistics. Specifically, we combined state-wise current-price value-of-output data with historical state-year production totals for the six focal cereals. On the production side, the revised pipeline already used a historical state-year denominator aggregated from the DES APY public report route, so the production term already retained state and year variability rather than relying on a single national (MSP-based) benchmark.

The revised manuscript now uses this combined information directly in the primary revenue benchmark where the matched data support it: for state-crop combinations with matched 2017–18 realized unit prices, the sales-revenue term uses the corresponding official realized price. This incorporates observed state and crop heterogeneity without implying false district-level precision.

The new analysis shows three things. First, realized-price benchmarks – when aggregated at a national scale – do not map one-to-one onto MSP. While we use state–crop-specific revenue information in our optimization analysis, this step was undertaken as a preliminary check to assess the consistency between the two price estimates. Second, indicative realized-price terms of trade among the coarse cereals are heterogeneous rather than uniformly favorable. Third, the realized-price dataset has a material scale mismatch with the district-level optimization and uneven crop coverage, particularly for jowar, bajra, and ragi in some states. For that reason, the revised core specification uses a primary revenue benchmark that combines direct realized state-year prices, where available, with transparent all-India realized-price fills when the official state-crop series is incomplete. We discuss this in the revised manuscript explicitly to avoid overstating the precision of our analysis and findings.

We also revised the main figures (Figure 2 and Figure 3) using results based on the new 2017–18 revenue benchmark. In the revised work, we also aligned the combined annual Pareto construction in Fig. 2a with the written seasonal formulation by summing the approved rabi and kharif objective totals at matched α values for annual interpretation. This new adjustment changes the absolute annual placement of the frontier – compared to earlier MSP-based results, but it does not alter the comparative ordering between water-focused and nitrogen-focused restructuring. The resulting endpoint gains are more conservative than under the MSP comparison case, but the comparative ranking between water-focused and nitrogen-focused restructuring remains unchanged. These additional experiments, therefore, add a more realistic market-based check while reinforcing the same central comparative insight. To further ascertain this aspect, the MSP results are now retained as comparison figures in Supplementary Figs. S17–S18, while Supplementary Fig. S16 summarizes the realized-price diagnostics that motivated the revised benchmark.

Changes in manuscript: Main manuscript lines 74, 168, 176, 191–196, 217, 297–315, 338–340, and 644–648; Supplementary Information lines 511–545 and Supplementary Figs. S16–S19.

Comment 2

Comment

Any inference on the crop reallocations obtained using MSP-based estimates on farmers income would be conditional on realization of MSP for each crop which is not a typical characteristic of Indian agricultural market. This refutes authors' claim of incorporating realistic agricultural scenario in the model. Underline conditionalities of the results shall at least be explicitly discussed in the paper.

Response

We thank the reviewer for this remark. This aspect is now addressed directly when we redid the

revenue analysis in response to the above Comment 1. Once the primary benchmark was rebuilt using the official realized value-of-output data together with the historical state-year production denominator, the interpretation of the income constraint was also tightened to match what the data support. The revised manuscript now uses that primary 2017–18 revenue benchmark in the main analysis, while retaining the MSP formulation as a transparent comparison case in the Supplementary Information.

We then tightened the language in the manuscript accordingly. In the framing paragraph of the main text, we now describe the model as imposing explicit calorie-adequacy and income-preservation constraints. In the Results, the net-return changes are described as outcomes under the primary 2017–18 revenue benchmark, and we state explicitly that these gains should be interpreted as benchmarked income preservation and modest improvement rather than literal district-specific realized remuneration. In the Methods, we define the sales-revenue price term as a state-crop benchmark assembled from matched realized prices and all-India realized-price fills for the residual unmatched combinations. In the Discussion, we now state any remaining limitations of our analysis, including the state-year scale mismatch, and refer readers to Supplementary Figs. S16–S18. In this way, the wording in the revised manuscript now better aligns with the results obtained from the official databases incorporated in response to Comment 1.

Changes in manuscript: Main manuscript lines 107, 168, 176, 191–196, 217, and 297–315; Supplementary Information lines 511–534 and Supplementary Figs. S16–S18.

Comment 3

Comment

Estimates on production cost used in the analysis need to be elaborated using standard terminology. It is not clear whether cost includes only paid-out expenses or imputed value of owned resources (family labour, own land, etc) as well. It is recommended to use standard cost concepts (eg Cost A1/Cost B1/Cost C1) for ensuring clarity and uniform comprehension.

Response

We have clarified this in the revised manuscript. The production-cost term used in the model is not a district-level reconstruction of separate material, labour, and land components as the previous wording may have implied. Instead, we used the published state-level crop-specific cost-of-production series reported in *Agricultural Statistics at a Glance 2017*[**?**]. In that source table, the cost-of-production column used in the model is reported in Rupee quintal^{-1} on the DES C2 basis, while cost of cultivation is separately reported on A2+FL and C2 bases in Rupee hectare^{-1}. We therefore revised the Methods to describe the production-cost term in exactly those terms and edited the corresponding equation and units to match the reported terminologies. We also checked the archived implementation directly: the coefficient multiplies production after tonne-to-quintal conversion, confirming that the executed term is output-based cost of production rather than a per-hectare cultivation-cost coefficient.

In the revised manuscript, we now state explicitly that this term should be interpreted as a state-level C2 cost-of-production benchmark applied uniformly to districts within each state-crop pair. Thus, it is not a narrow paid-out-cost measure alone; through the DES C2 definition, it includes imputed owned-resource components such as the rental value of owned land and the imputed value of family labour. At the same time, it should not be interpreted as a district-level microdata estimate of farm costs. To improve clarity, we added a supplementary table summarizing the standard DES concepts (Cost A1, Cost A2, A2+FL, Cost B1, Cost B2, Cost C1, and Cost C2) using the DES manual cited by the reviewer. We also added a second supplementary table that makes the aggregation explicit by listing the native source resolution, native units, and exact treatment of the

production-cost and revenue benchmarks within the optimization.
Changes in manuscript: Main manuscript lines 297–315 and 217; Supplementary Information lines 564–607 and Supplementary Tables 10–11.

Comment 4

Comment

Usually alternate crops like jowar, bajra, ragi and maize remain less remunerative than rice and wheat, disincentivizing farmers to diversify away from the latter crops. As the resultant crop reallocations are in favour of alternate crops (jowar, bajra, ragi and maize), it would be plausible to provide revenue terms of trade between competing crops as Supplementary information.

Response

We agree and have added this in the revised supplementary material. Supplementary Fig. S16b now reports indicative realized-price terms of trade of the alternative cereals relative to rice and wheat, summarized from the official realized-price benchmark. This lets readers assess directly whether the relative price signals are uniformly favorable or heterogeneous across the alternative cereals. The figure shows the latter, which is why the primary manuscript uses matched realized prices where available but retains a transparent all-India realized-price fill for the small number of uncovered state-crop combinations.
Changes in manuscript: Supplementary Information lines 511–516 and Supplementary Fig. S16.

Comment 5

Comment

It would be revealing if it is possible to provide seasonally disaggregated optimized crop reallocations. Although the crops like maize and rice are cultivated in both kharif and rabi seasons in few states, seasonal typology is generally maintained by other crops. Figure 2 shows reallocation of wheat area to alternate crops- jowar, bajra, and ragi, typically grown in different seasons. Similarly, some area from rice has been reallocated to wheat which is counter-intuitive as in majority of wheat (rabi) growing states, rice (kharif) is not a competing crop. A clear seasonally-disaggregated picture on crop substitution between competing crops would provide a better comprehension of the results. At least, districts witnessing such counter-intuitive reallocations shall be demarcated.

Response

We agree that the annual crop-reallocation flow summary in Fig. 2d is easier to interpret when its seasonal structure is shown explicitly. The optimization itself is already seasonal: kharif and rabi are solved independently and the annual panel aggregates those two primary seasonal solutions. We therefore added a dedicated seasonal breakdown in the Supplementary Information as Supplementary Fig. S20.

In that new figure, we now show separate kharif and rabi crop-reallocation flow summaries derived from the primary nitrogen-focused optimized area table, together with a direct identification of district-seasons in which rice decreases while wheat increases. This seasonal disaggregation clarifies the issue raised by the reviewer. In the primary solution table, there is no baseline kharif wheat area. Accordingly, the apparent annual rice-to-wheat crossover in the combined panel does not represent a kharif same-season substitution. The limited same-season rice-loss / wheat-gain adjustment occurs only in rabi districts that already cultivate rabi rice in the baseline system. These districts are concentrated primarily in West Bengal, with smaller contributions from a limited set of other rabi rice-growing districts.

This addition allows us to keep the main annual panel as a compact annual summary while making the seasonal interpretation explicit in the Supplementary Information.

Changes in manuscript: Main manuscript line 217; Supplementary Information lines 551–559 and Supplementary Fig. S20.

Comment 6

Comment

As calorie content of selected crops is almost similar (see Supplementary table 4), imposing restriction of 'calorie production' in the model for the sake of food security becomes redundant. However, alternate crops viz. jowar, bajra, ragi, maize still offer advantage over rice and wheat in terms of overall nutritional and environmental effects. The authors shall emphasize nutritional security dimension of alternate crops apart from calorie production (food security). This will align the paper with the shifting policy discourse from food to nutritional security in India and globally.

Response

Thanks for reframing this excellent point. In response, we revised the manuscript so that the calorie constraint is interpreted narrowly as a conservative energy-availability floor, not as a direct optimization of full food security or broader nutritional adequacy. This change has been made consistently in the Methods, Results, Discussion, and trade-network interpretation.

To address the second part of the comment, we added a supplementary nutritional comparison of the six focal cereals. This table shows that the cereals are broadly similar in calorie density but differ materially in protein and micronutrient content, especially iron and calcium. We now state explicitly that diversification toward coarse cereals may confer broader nutritional and environmental co-benefits.

Changes in manuscript: Main manuscript lines 168, 174, 191, 213, 226–227, 328, 338, and 379–390; Supplementary Information lines 380–398.

Comment 7

Comment

The scope of the analysis is limited to cereals crops and surprisingly excludes the nitrogen-friendly pulses/leguminous crops. The ability of pulses to fix atmoshpheric nitrogen naturally shall make these crops a preferred element of the alternate crops sub-set, particularly in the studies like this where nutrient management is a core investigation. Also, farmers' decision on crop choices are not mutually exclusive to crop groups (eg cereals, pulses, oilseeds, fruits, vegetables). Depending on factors like resources constraints, relative profitability, risk perception etc, farmers often grow a combination of different crops. The authors can discuss the importance of pulses in nitrogen-management and possibly mention the reasons for choosing only cereals crops over similar or even better alternatives like pulses. Future work plan on leveraging potential of pulses in nitrogen management can be outlined.

Response

Thanks for raising this point. In response, we strengthened the Discussion and stated this scope boundary explicitly, together with the rationale for the cereal-only focus used in the present analysis.

Specifically, we now explain that the study focuses on six major cereals because both the optimization and the interstate trade-network reconstruction could be parameterized consistently for the dominant traded cereal system using harmonized district-season data on area, yield, input use, prices, calories, and trade. We also state clearly that the present results should therefore be

interpreted as evidence for cereal-to-cereal restructuring within India's major traded cereal system, especially the replacement of rice and wheat with lower-footprint alternative cereals, rather than as an all-crop optimization. In the same Discussion paragraph, we now note that pulses and other legumes remain important for nitrogen management and dietary diversification, and that extending the framework to mixed cereal–pulse systems is an important next step that will require harmonized district-season data for additional crop groups.

Changes in manuscript: Main manuscript lines 107 and 215.

Comment 8

Comment

It is not clear how biological nitrogen fixation (BNF) for cereals (non-leguminous) crops have been estimated as these crops do not nodulate and data on crop-specific application of biofertilizer at district/state level is not available in cost of cultivation surveys used in the study. The supplementary figure 1 and text mentions the example of legume crops which is excluded from the analyses). For the sake of clarity, it is desirable to provide a supplementary table on BNF estimates for selected crops, as given for other variables.

Response

In this revision, we used this comment as an opportunity to make the treatment of BNF explicit and unambiguous for the cereals-only framework used in the study.

The revised manuscript now clarifies that the BNF term was not estimated as a crop-specific symbiotic fixation rate for the focal cereals. Instead, we used an established approach based on the FAOSTAT Cropland Nutrient Budget BNF rate, merged by year into the district-crop database and carried into the nitrogen balance as an exogenous cropland input term. Because the focal crops are cereals, this term should not be interpreted as nodulating biological fixation by the cereal plants themselves or as district-level biofertilizer application. Regarding nitrogen surplus balancing across Indian districts, we refer to our previously published work (Goyal et al., 2024), in which we provide a detailed description of nitrogen balances across districts, accounting for different crops and budget components (e.g., fertilizer inputs, manure, nitrogen uptake, and biological nitrogen fixation).

To take the reviewer point more explicit for this paper itself, we removed the generic legume example from the Methods, revised the description of N_{BNF} in the nutrient-surplus equations, and added Supplementary Table 12 reporting the implemented BNF term for each selected cereal. In the 2017 baseline optimization used in this study, the same annual FAOSTAT cropland BNF term is applied across the six focal cereals, and the implemented value is now reported explicitly in the Supplementary Information.

Goyal, S.S., Kumar, R. & Bhatia, U. Assessing temporal dynamics of nitrogen surplus in Indian agriculture: district scale data from 1966 to 2017. Sci Data 11, 1191 (2024). https://doi.org/10.1038/s41597-024-04023-3

Changes in manuscript: Main manuscript lines 245 and 263–270; Supplementary Information lines 611–629 and Supplementary Table 12.

Comment 9

Comment

The authors may provide source of State-specific fractions of residue brunt in Supplementary table 6. This would be useful for reader as such data is often not widely known.

Response

We have now made the source attribution explicit in both the Methods and the Supplementary Information text. Specifically, Supplementary Table 6 now states that the state-specific rice and wheat residue-burning fractions follow the revised residue-burning inventory reported by Jain et al. (2014), and we also state that the remaining cereal-state combinations use the default value of 0.10 adopted in that inventory. The corresponding Methods paragraph on crop-residue burning now refer to the same source directly and points readers to Supplementary Table 6 for the listed state-level information.

Changes in manuscript: Main manuscript line 534; Supplementary Information lines 425–440 and Supplementary Table 6.

Response

Taken together, Reviewer #6's comments improved the manuscript substantially. They gave us the opportunity to make the benchmark construction, assumptions, and supporting analyses more explicit, demonstrate the robustness of the core findings through additional analyses, and make the income, calorie, and scope-related claims clearer and unambiguous. Importantly, the additional analyses introduced in response to these comments, including the realized-price primary benchmark, the district-MSP comparison analysis, and seasonal disaggregation, all support the same core comparative conclusion. The central environmental and restructuring insights, therefore, remain intact, and they are now presented with a stronger basis and clearer interpretation.
